# Meal analysis for understanding eating behavior: meal- and participant-specific predictors for the variance in energy and macronutrient intake

**DOI:** 10.1186/s12937-019-0440-8

**Published:** 2019-03-07

**Authors:** Carolina Schwedhelm, Khalid Iqbal, Lukas Schwingshackl, George O. Agogo, Heiner Boeing, Sven Knüppel

**Affiliations:** 1 Department of Epidemiology, German Institute of Human Nutrition Potsdam-Rehbruecke (DIfE), Arthur-Scheunert-Allee 114-116, 14558 Nuthetal, Germany; 2 NutriAct – Competence Cluster Nutrition Research, Berlin-Potsdam, Germany; 3 Institute for Evidence in Medicine, Faculty of Medicine and Medical Center - University of Freiburg, Freiburg, Germany; 4 Department of Internal Medicine, Yale School of Medicine, New Haven, CT USA; 5 Department of Nutrition and Gerontology, German Institute of Human Nutrition Potsdam-Rehbruecke (DIfE), Arthur-Scheunert-Allee 114-116, 14558 Nuthetal, Germany

**Keywords:** Sources of variation, Dietary intake, Meal-based analysis, Multilevel analysis, EPIC Potsdam study

## Abstract

**Background:**

Meals differ in their nutritional content. This variation has not been fully addressed despite its potential contribution in understanding eating behavior. The aim of this study was to investigate the between-meal and between-individual variance in energy and macronutrient intake as a measure of variation in intake and the meal type-specific relative importance of predictors of these intake variations.

**Methods:**

Energy and macronutrient intake were derived from three 24 h dietary recalls in an EPIC-Potsdam sub-cohort of 814 German adults. Intra-class correlation was calculated for participants and meal type. Predictors of intake were assessed using meal type-specific multilevel regression models in a structural equation modeling framework at intake and participant levels using the Pratt Index. The importance of the predictor energy misreporting was assessed in sensitivity analyses on 682 participants. 95% confidence intervals were calculated based on 1000 bootstrap samples.

**Results:**

Differences between meal types explain a large proportion of the variation in intake (intra-class correlation: 39% for energy, 25% for carbohydrates, 47% for protein, and 33% for fat). Between-participant variation in intake was much lower, with a maximum of 3% for carbohydrate and fat. Place of meal was the most important intake-level predictor of energy and macronutrient intake (Pratt Index of up to 65%). Week/weekend day was important in the breakfast meal, and prior interval (hours passed since last meal) was important for the afternoon snack and dinner. On the participant level, sex was the most important predictor, with Pratt Index of up to 95 and 59% in the main and in the sensitivity analysis, respectively. Energy misreporting was especially important at the afternoon snack, accounting for up to 69% of the explained variance.

**Conclusions:**

The meal type explains the highest variation in energy and macronutrient intakes. We identified key predictors of variation in the intake and in the participant levels. These findings suggest that successful dietary modification efforts should focus on improving specific meals.

**Electronic supplementary material:**

The online version of this article (10.1186/s12937-019-0440-8) contains supplementary material, which is available to authorized users.

## Background

Research in nutritional epidemiology is increasingly focusing on meals [1, 2], which provide the structure of eating behavior. Eating behavior is explained through a complex interaction of biological, psychological, sociocultural, and contextual factors [3]. Eating behavior is known to vary within population subgroups, such as by sex, age groups, and socioeconomic status [4, 5]. For instance, age, sex, self-efficacy, and environment (home, work, and church) are shown to be associated with fat intake [3]. However, there is limited knowledge on how dietary intake across meals relates to individual and meal-level factors [6]. Studying meals and their surrounding factors might contribute towards understanding of overall dietary intake and eating behavior [7]. Moreover, dietary advice on meals could be an intervention on changing dietary intake [6, 8].

Diet is composed of foods consumed in different amounts across meals, days, and by different individuals, resulting in intake variations at different levels. Hitherto, the within-individual variation has been investigated across days to determine the minimum number of dietary records needed to precisely calculate the usual diet [5]. Variance components are used to calculate usual/habitual intake, which takes into account between-individual and within-individual variation. Using this approach, the day-to-day variation is identified as a source of measurement error [9].

In such analyses, there is no within-meal variation because intakes are averaged out to estimate usual/habitual intake. Ignoring meal type as another level of variation in the model underestimates the total variation in dietary intake. This variation might help to understand dietary intake better.

The aim of this study was to investigate the contribution of the meal type and individuals in explaining energy and macronutrient intake variation. We identified important sources of variation and predictors of energy and macronutrient intake.

## Methods

### Study design

Data from a validation sub-study of the EPIC-Potsdam cohort (2010–2012) was used. Participants of the EPIC-Potsdam study who were still actively taking part in follow-up interviews were eligible to join the study. Details on the study design of the EPIC-Potsdam study are available elsewhere [10, 11]. For the validation sub-study, individuals were invited based on a random age and sex stratified sample of the eligible EPIC-Potsdam study participants. Recruitment started in August 2010 through 2012. All participants gave informed consent and the study was approved by the Ethics Committee of the Medical Association of the State of Brandenburg [12].

One study participant was excluded from the analysis due to dementia. Therefore, the analyses were based on a sample of 814 men and women (Additional file 1: Figure S1). This study is reported according to the Strengthening the Reporting of Observational Studies in Epidemiology-nutritional epidemiology (STROBE-nut) checklist [13] (Additional file 2).

### Dietary assessment

Participants provided up to three 24 h dietary recalls (24hDR) (mean = 3). The first 24hDR was recorded during the first study center visit by a trained interviewer. The following two 24hDR were administered over the telephone on randomly chosen days by trained interviewers. All records were collected using the standardized computerized 24hDR program EPIC-Soft [14] (renamed GloboDiet in 2014) within 4–24 months (mean = 7 months). Food intake was documented in grams for every eating occasion (11 eating occasions per day) and was converted into nutrients using the German nutrient database ‘Bundeslebensmittel-schlüssel’ (BLS, version 3.01). The full list of the 11 eating occasions with corresponding mean meal times and standard deviations is available in Additional file 1: Table S1. Consistent with our previous publication, four participant-identified meals were retained for the main meal analysis: breakfast, lunch, afternoon snack, and dinner [15].

### Measurement of other study variables

Sociodemographic and lifestyle data were collected through self-reported questionnaires during the first study center visit. Body mass index (BMI) was calculated as the ratio of weight in kg to height squared in meters. Body weight and height were measured in the study center following standardized protocols consistent with WHO guidelines [16]. Energy expenditure was measured with a combined heart rate and uniaxial movement sensor (Actiheart, CamNtech, Cambridge, UK) [17], which was worn at the chest continuously during 7 consecutive days. These data are available for 682 of the 814 study participants. Total energy expenditure (TEE) was calculated from the Actiheart-device as the sum of activity energy expenditure, diet-induced thermogenesis (as 10% of TEE), and resting energy expenditure (from the Schoefield Equations) [18, 19].

### Statistical methods

Energy intake was measured in kilocalories (kcal) per meal and macronutrients in grams per meal. For each outcome variable, we excluded zero values from analysis and log transformed the non-zero values to achieve a normal distribution. The zero values were mostly from energy-free beverages such as water (with 0 kcal and 0 g for all macronutrients) and sweetened beverages, including coffee with sugar (with 0 g of fat and protein). Due to their nature and low occurrence, exclusion of zero values was unlikely to bias the data. The frequency of the excluded zero values was 251 (2.8%) for energy, 242 (2.7%) for carbohydrates, 305 (3.3%) for protein, and 449 (4.9%) for fat. The hierarchical structure of the data is as follows: participant (level 3), meal type (level 2), and the intake level (level 1). We fit multilevel regression models with random intercepts for participant and meal type, allowing these to vary in dietary intake. Participants with 1 recall (*n* = 3) contributed to the inter-individual variation (level 3) but not the intra-individual variation (levels 1 and 2).

The intra-class correlation coefficients (ICC) were calculated in the intercept-only model to obtain the proportion of variance in each level, where variance is a measure of variation [20, 21]. Details on ICC calculation are available in Additional file 1: Box 1.

We then added the following relevant covariates: *sex*, *age*, *BMI*, *physical activity*, *education level*, *current occupation*, *smoking status*, *duration of prior interval*, *place of meal*, *special day*, *season*, and *week/weekend day* to the multilevel regression model to measure their relative importance in explaining the variation in the outcome variables for each level in in a structural equation modeling (SEM) framework. This approach allows modeling of complex relationships between variables and their ordering into the different levels of the multilevel regression analysis, providing level-specific covariance and correlation matrices, whereas conventional multilevel models or hierarchical linear models (HLM) do not allow this break-down [22, 23]. Details on the selection and description of covariates are available in Additional file 1: Box 2. Intake-level covariates were added to the first level (specific meal on a specific day) and participant-level covariates were added to the highest level (participant level). Since no covariates are specific to meal type (i.e., same for all breakfast meals, all lunch meals, etc.), two-level models stratified by meal type were fitted (level 1: intake level; level 2: participant level).

We used the methods described by Liu et al. [23] for calculating the Pratt Index (PI), which represents the proportion of R^2^ explained by each explanatory variable, ordering predictors in terms of their importance in a multiple regression analysis. A detailed description of the calculation of the PI is available in Additional file 1: Box 3. Results can be interpreted as the meal type-specific relative importance of predictors in the intake and participant levels, respectively. A detailed description of the main models can be found in Additional file 1: Box 4. Bootstrap confidence intervals (95%CI) were calculated for the following parameters: standardized regression coefficient (beta-weight), correlations, R^2^, as well as the PI by taking the 2.5 and 97.5% percentiles from the resulting estimates from 1000 bootstrap samples [24]. Bootstrap samples were drawn by selecting participants with replacement (method described in detail in Additional file 1: Box 5). Statistical software SAS, version 9.4, and SAS Enterprise Guide, version 6.1 (SAS Institute, Cary, NC) was used for statistical analysis. Multilevel regression analyses were done using MPlus Version 7 (Muthén & Muthén, Los Angeles, CA, USA). Additionally, R was used for automation of MPlus model estimation for bootstrap confidence intervals [25].

In sensitivity analyses, we adjusted for *energy misreporting* for 682 participants with data on energy expenditure, adding *energy misreporting* as a categorical variable (indicating underreporting, overreporting, or plausible reporting) to the participant level / between model. *Energy misreporting* was calculated and used by *Gottschald* et al. [26] based on a cutoff of ±1 SD for the energy intake (EI) to TEE ratio according to sex, age, and BMI stratified estimates of variation published by Huang et al. [27] using usual energy intake calculated with the NCI method [9, 28]. A ratio of EI/TEE < 0.81 indicates underreporting and a ratio of > 1.19 is indicative of overreporting [26, 27].

## Results

The study participants were, on average, 65.5 years old (ranging from 47 to 81), had a mean BMI of 27.5 kg/m^2^, and on average did 22.6 h of physical activity per week. Further, 10.3% of participants were current smokers. Most men were former smokers (57.2%) and most women were never smokers (60.8%). Participants who had a university degree (44.2%) were more than those without a vocational training (32.8%) or those who had a technical college degree (23%). More men than women had a university degree (54.5% vs 33.7%). Most participants did not have a current occupation (62%). Underreporting of energy intake (EI/TEE < 0.81) was more common in women than in men and it was present in 39.6% of all participants (Table 1). Participants’ mean energy and macronutrient intakes by day and by meal-type are shown in Table 2 for men, women, and for all participants. In general, intakes of all dietary variables were lower among women than among men. The meal with the highest energy intake was dinner for men and lunch for women. Carbohydrate and protein intake were highest during lunch, while fat intake was highest during dinner (both in men and women).

**Table 1:** Selected baseline socio-demographic and lifestyle characteristics of the studied population sample

Characteristics	Total	Men	Women
*n* (%)	814 (100)	411 (50.5)	403 (49.5)
Age, y	65.5 ± 8.4^a^	66.4 ± 8.0	64.5 ± 8.7
BMI, kg/m^2^	27.5 ± 4.4	27.7 ± 3.9	27.4 ± 4.8
Hours of physical activity/week^b^	22.6 ± 14.7	20.7 ± 14.0	24.7 ± 15.0
Smoking status (%)			
Never smoker	377 (46.3)	132 (32.1)	245 (60.8)
Former smoker	353 (43.4)	235 (57.2)	118 (29.3)
Current smoker	84 (10.3)	44 (10.7)	40 (9.9)
Education (%)			
No vocational training / current training	267 (32.8)	124 (30.2)	143 (35.5)
Technical college	187 (23.0)	63 (15.3)	124 (30.8)
University	360 (44.2)	224 (54.5)	136 (33.7)
Occupation (%)			
Full time (> 35 h/week)	248 (30.5)	141 (34.3)	107 (26.7)
Part time/hourly (< 35 h/week)	61 (7.5)	18 (4.4)	43 (10.7)
No job/retired	505 (62.0)	252 (61.3)	253 (62.8)
Energy misreporting (%)^c^			
EI/TEE < 0.81	270 (39.6)	118 (34.0)	152 (45.4)
0.81 < EI/TEE < 1.19	359 (52.6)	187 (53.9)	172 (51.3)
EI/TEE > 1.19	53 (7.8)	42 (12.1)	11 (3.3)

^a^Mean ± SD (all such values)

^b^self-reported. Includes the following activities done in the past 12 months: sports, gardening, physical work, housework, cycling

^c^*n* = 682; *EI* energy intake, *TEE* total energy expenditure

**Table 2:** Mean participants’ dietary intake

Intake variable	Day^a^ (*n* = 814)	Breakfast (n = 814)	Lunch (*n* = 808)	Afternoon snack (*n* = 804)	Dinner (n = 814)
Energy, kcal					
All	2058 ± 593^b^	451 ± 199	528 ± 224	263 ± 191	524 ± 222
Men	2341 ± 600	521 ± 213	583.3 ± 249	292 ± 208	609 ± 230
Women	1770 ± 422	380 ± 154	471 ± 177	232 ± 167	438 ± 175
Carbohydrate, g					
All	204.1 ± 62.2	50.1 ± 22.8	46.9 ± 24.5	30.6 ± 21.4	40.6 ± 19.9
Men	226.6 ± 66.9	56.2 ± 25.5	51.6 ± 28.0	33.7 ± 22.3	46.4 ± 22.3
Women	181.2 ± 47.0	44.0 ± 17.8	42.2 ± 19.1	27.5 ± 10.0	34.6 ± 14.9
Protein, g					
All	74.5 ± 23.8	14.5 ± 8.2	26.1 ± 15.2	6.4 ± 6.9	22.2 ± 11.6
Men	84.0 ± 24.6	16.8 ± 9.1	29.1 ± 16.8	7.0 ± 7.9	25.4 ± 11.8
Women	64.8 ± 18.4	12.2 ± 6.3	23.1 ± 12.6	5.8 ± 5.8	19.0 ± 10.5
Fat, g					
All	93.0 ± 33.0	21.1 ± 12.6	24.7 ± 12.8	12.0 ± 10.4	27.4 ± 13.9
Men	106.5 ± 33.8	25.1 ± 13.8	27.2 ± 14.1	13.4 ± 11.5	32.1 ± 14.4
Women	79.1 ± 25.7	16.9 ± 9.7	22.2 ± 10.7	10.5 ± 9.0	22.7 ± 11.6

^a^All 11 eating occasions

^b^Mean ± SD

**Fig. 1:**
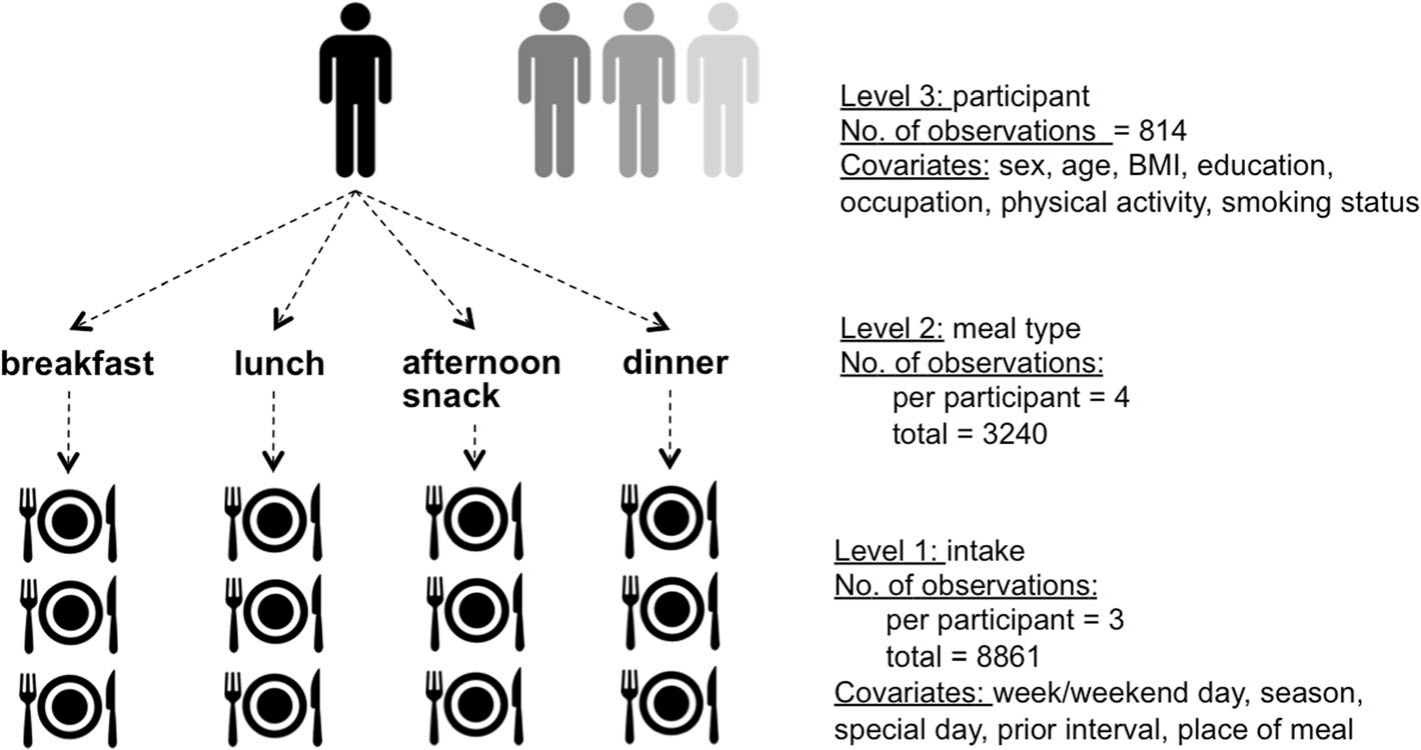
Hierarchical structure of the data

Structured by individual intakes (level 1) clustered by meal type (level 2), which are then clustered by participant (level 3), our data shows a 3 level-hierarchical structure (Fig. 1). The details on the total number of observations and observations per meal and participant are in Additional file 1: Table S2.

### Proportions of variance between participants and between meals

**Fig. 2:**
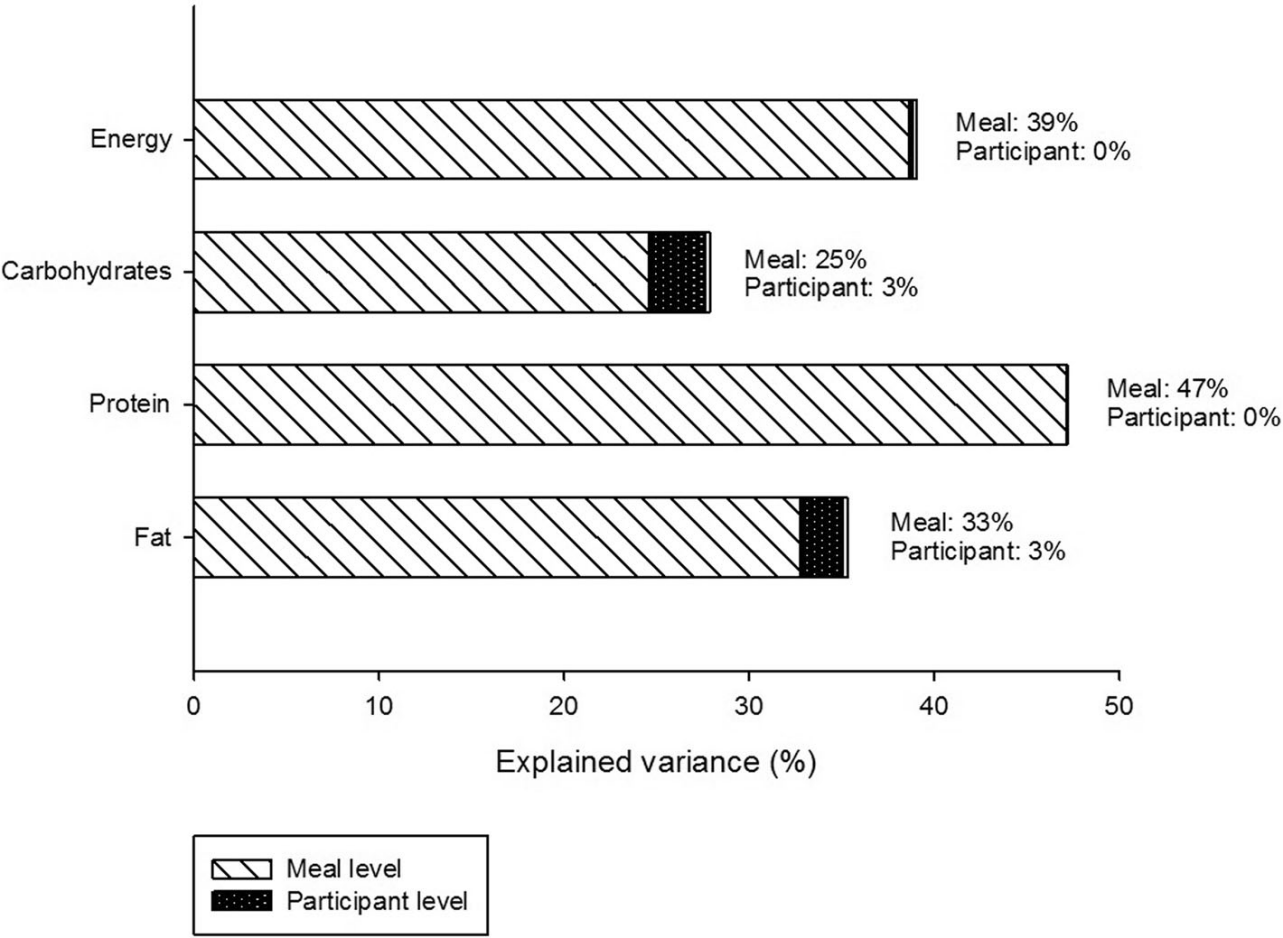
Percent explained variance for energy and macronutrient intake by meal and participant levels

Overall, large proportions of the variance were explained by differences between meal types for all dietary variables. For energy intake, variance explained in the meal level was 39%. For macronutrients, this was by decreasing order as follows: 47, 33, and 25% for protein, fat, and carbohydrates, respectively. In contrast, variance explained by differences across participants was very low: 0% for energy and protein intake and 3% for carbohydrate and fat intake (Fig. 2). The remaining, non-explained variance (adding up to 100%) was inherent to the first level, corresponding to differences between intake occasions in general (intake level; specific meal on a specific meal).

### Predictors of the explained variance in energy and macronutrient intake by meal type

Table 3 shows the relative importance of intake-level and participant-level covariates to the explained variance in energy intake and Table 4 shows the results for energy intake while also adjusting for energy misreporting (sensitivity analysis). Due to the large amount of tables, results on the macronutrients can be found in the Additional file 1: Tables S3 and S4 (for the results overview (PI) of main and sensitivity analyses, respectively). These results are still presented and discussed within the text of this manuscript. Additional file 1: Tables S5, S6, S7, S8, S9, S10, S11 and S12 show the detailed results of the random intercept multilevel regression analysis and corresponding PI for the main analysis (Additional file 1: Tables S5, S6, S7 and S8) and sensitivity analysis (Additional file 1: Tables S9, S10, S11 and S12).

**Table 3:** Relative importance of predictors of energy intake (kcal/meal)^a^

Covariates^b^	Breakfast			Lunch			Afternoon snack			Dinner		
	Beta-weight (95%CI)^c^	Correlation (95%CI)	Pratt Index (95%CI)	Beta-weight (95%CI)	Correlation (95%CI)	Pratt Index (95%CI)	Beta-weight (95%CI)	Correlation (95%CI)	Pratt Index (95%CI)	Beta-weight (95%CI)	Correlation (95%CI)	Pratt Index (95%CI)
Intake-level covariates												
Week/weekend day (y/n)	0.11 (0.06;0.17)	0.12 (0.07;0.18)	**27%**^d^ (9;49)	0.04 (− 0.01;0.08)	0.05 (0.00;0.10)	6% (0;25)	0.10 (0.05;0.15)	0.13 (0.08;0.18)	**11%** (0.03;0.23)	−0.04 (− 0.09;0.01)	− 0.03 (− 0.07;0.02)	3% (0;17)
Season (winter/ summer)	−0.04 (− 0.086;0.01)	−0.04 (− 0.09;0.01)	3% (0;17)	0.01 (− 0.04;0.06)	0.00 (− 0.05;0.05)	0% (0;10)	0.04 (− 0.01;0.08)	0.03 (− 0.02;0.08)	1% (0;5)	0.04 (− 0.01;0.09)	0.04 (− 0.01;0.09)	5% (0;19)
Special day (y/n)	0.01 (− 0.06;0.07)	0.05 (− 0.01;0.11)	1% (− 2;14)	0.04 (− 0.01;0.09)	0.05 (− 0.00;0.10)	7% (0;29)	0.05 (− 0.00;0.10)	0.10 (0.04;0.15)	5% (0;12)	0.06 (0.01;0.11)	0.08 (0.04;0.13)	**15%** (1;36)
Prior interval (hours)	− 0.02 (− 0.08;0.04)	− 0.01 (− 0.08;0.05)	1% (0;14)	0.06 (0.01;0.10)	0.07 (0.03;0.11)	**14%** (1;41)	0.17 (0.12;0.21)	0.21 (0.15;0.25)	**29%** (16;43)	0.07 (0.03;0.12)	0.09 (0.04;0.13)	**20%** (3;40)
Place of meal (ref: home)												
work	−0.14 (− 0.27;-0.02)	− 0.15 (− 0.28;-0.02)	**41%** (1;72)	−0.14 (− 0.22;-0.06)	−0.151 (− 0.225;-0.073)	**75%** (23;90)	−0.21 (− 0.28;-0.14)	−0.26 (− 0.32;-0.19)	**45%** (24;61)	−0.08 (− 0.17;-0.01)	−0.09 (− 0.17;− 0.01)	**21%** (0;55)
restaurant	0.10 (0.06;0.15)	0.11 (0.07;0.16)	**24%** (6;50)	-0.01 (−0.06;0.05)	0.03 (−0.03;0.08)	0% (− 1;14)	0.02 (− 0.04;0.07)	0.04 (− 0.02;0.09)	1% (0;6)	0.10 (0.06;0.15)	0.12 (0.07;0.16)	**36%** (12;57)
other	0.04 (−0.00;0.09)	0.04 (−0.00;0.09)	4% (0;017)	−0.02 (− 0.07;0.04)	0.01 (− 0.04;0.06)	0% (− 1;12)	0.08 (0.03;0.13)	0.13 (0.08;0.18)	9% (2;20)	− 0.01 (− 0.08;0.06)	0.00 (− 0.07;0.07)	0% (0;17)
R-squared (95%CI)	0.05 (0.03;0.11)			0.03 (0.01;0.06)			0.12 (0.09;0.16)			0.03 (0.02;0.06)		
Participant-level covariates												
BMI (kg/m^2^)	0.00 (− 0.10;0.10)	−0.03 (− 0.13;0.07)	0% (− 1;5)	0.08 (− 0.06;0.23)	−0.01 (− 0.14;0.12)	0% (− 1;6)	0.09 (− 0.04;0.22)	−0.04 (− 0.17;0.09)	0% (− 2;8)	0.18 (0.07;0.29)	0.09 (− 0.02;0.20)	4% (0;12)
Age (years)	0.08 (− 0.05;0.21)	0.21 (0.12;0.29)	7% (−3;19)	0.02 (− 0.19;0.19)	0.26 (0.10;0.40)	1% (−6;16)	− 0.10 (− 0.28;0.09)	0.02 (− 0.11;0.16)	0% (− 6;10)	−0.22 (− 0.36;-0.07)	−0.05 (− 0.15;0.06)	2% (− 2;10)
Sex (M/W)	− 0.31 (− 0.41;-0.22)	−0.35 (− 0.43;-0.28)	**44%** (23;59)	−0.38 (− 0.57;-0.23)	−0.38 (− 0.56;-0.25)	**35%** (14;51)	−0.23 (− 0.36;-0.11)	−0.23 (− 0.35;-0.12)	**23%** (5;39)	− 0.41 (− 0.50;-0.33)	−0.48 (− 0.57;-0.40)	**48%** (32;59)
Education level (ref. current/no training)												
technical college	−0.01 (− 0.10;0.08)	−0.07 (− 0.15;0.020)	0% (− 1;6)	− 0.03 (− 0.16;0.11)	−0.05 (− 0.20;0.09)	0% (− 1;5)	0.08 (− 0.06;0.21)	−0.01 (− 0.14;0.11)	0% (− 2;8)	0.03 (− 0.07;0.14)	−0.10 (− 0.20;-0.00)	0% (− 2;3)
university	− 0.01 (− 0.11;0.08)	0.06 (− 0.03;0.14)	0% (− 1;4)	− 0.08 (− 0.23;0.06)	−0.02 (− 0.15;0.13)	0% (−1;6)	0.04 (− 0.10;0.18)	0.08 (− 0.05;0.20)	1% (− 1;11)	0.08 (− 0.04;0.19)	0.15 (0.05;0.26)	3% (− 1;11)
Occupation (ref. no job/ retired)^e^												
full time	0.05 (− 0.11;0.20)	− 0.05 (− 0.15;0.06)	0% (− 3;6)	−0.033 (− 0.233;0.177)	−0.166 (− 0.359;0.013)	1% (− 3;14)	0.05 (− 0.16;0.26)	0.02 (− 0.14;0.17)	1% (− 2;13)	− 0.00 (− 0.15;0.14)	0.07 (− 0.04;0.18)	0% (− 2;4)
part time/hourly	−0.08 (− 0.19;0.04)	− 0.17 (− 0.28;-0.05)	5% (− 1;17)	0.017 (− 0.15;0.175)	− 0.061 (− 0.225;0.095)	0% (− 1;7)	0.03 (− 0.08;0.15)	− 0.00 (− 0.12;0.10)	0% (− 1;6)	0.05 (− 0.05;0.15)	0.02 (− 0.09;0.12)	0% (0;4)
Physical activity (h/week)	0.05 (− 0.03;0.13)	0.05 (− 0.03;0.12)	1% (0;6)	0.145 (0.013;0.280)	0.162 (0.041;0.290)	6% (0;14)	0.02 (− 0.09;0.12)	− 0.01 (− 0.12;0.10)	0% (0;5)	0.05 (− 0.04;0.13)	− 0.04 (− 0.13;0.06)	0% (− 1;2)
Smoking status (ref. never smoker)												
current smoker	0.27 (0.06;0.45)	0.04 (− 0.05;0.13)	4% (−4;17)	0.186 (− 0.064;0.449)	0.076 (− 0.057;0.202)	3% (− 1;15)	0.18 (− 0.03;0.40)	0.12 (0.00;0.24)	9% (− 1;29)	0.03 (− 0.17;0.23)	− 0.17 (− 0.27;-0.07)	0% (− 7;9)
former smoker	0.20 (0.01;0.40)	0.06 (− 0.03;0.15)	5% (− 1;18)	0.067 (− 0.187;0.351)	− 0.014 (− 0.143;0.130)	0% (− 2;7)	0.02 (− 0.21;0.24)	− 0.08 (− 0.20;0.04)	0% (− 6;12)	0.09 (− 0.10;0.28)	0.18 (0.07;0.28)	4% (− 3;15)
Energy misreporting												
EI/TEE < 0.81	− 0.15 (− 0.25;-0.07)	− 0.26 (− 0.35;-0.18)	**16%** (5;31)	−0.372 (− 0.539;-0.227)	−0.462 (− 0.603;-0.343)	**41%** (18;58)	−0.37 (− 0.52;-0.25)	−0.38 (− 0.53;-0.26)	**61%** (30;74)	−0.30 (− 0.42;-0.19)	−0.31 (− 0.41;-0.21)	**23%** (10;35)
EI/TEE > 1.19	0.18 (0.11;0.25)	0.27 (0.20;0.33)	**19%** (8;30)	0.159 (0.058;0.278)	0.311 (0.212;0.440)	**12%** (3;22)	0.10 (−0.03;0.23)	0.19 (0.05;0.32)	8% (−1;24)	0.24 (0.16;0.31)	0.31 (0.23;0.38)	**18%** (9;27)
R-squared (95%CI)	0.25 (0.20;0.36)			0.42 (0.31;0.78)			0.23 (0.16;0.44)			0.41 (0.34;0.56)		

^a^Pratt Index, in % contribution to the variance explained by the model (R^2^). Might not add up to 100% due to rounding errors from parameter estimates

^b^for dichotomous variables, the information shown is for the underlined category (reference category not underlined)

^c^all 95% confidence intervals (95%CI) – for beta-weights, correlations, r-squared, and Pratt Index – are bootstrap confidence intervals based on 1000 samples

^d^bold numbers indicate covariates accounting for > 10% of the explained variance

^e^full time: > 35 h/week; part time/hourly: < 35 h/week

**Table 4:** Relative importance of predictors of energy intake (kcal/meal); sensitivity analysis adjusting for energy misreporting^a,b^

Covariates^c^	Breakfast			Lunch			Afternoon snack			Dinner		
	Beta-weight (95%CI)^d^	Correlation (95%CI)	Pratt Index (95%CI)	Beta-weight (95%CI)	Correlation (95%CI)	Pratt Index (95%CI)	Beta-weight (95%CI)	Correlation (95%CI)	Pratt Index (95%CI)	Beta-weight (95%CI)	Correlation (95%CI)	Pratt Index (95%CI)
Intake-level covariates												
Week/weekend day (y/n)	0.11 (0.06;0.17)	0.12 (0.07;0.18)	**27%**^e^ (9;49)	0.04 (− 0.01;0.08)	0.05 (0.00;0.10)	6% (0;25)	0.10 (0.05;0.15)	0.13 (0.08;0.18)	**11%** (3;23)	−0.04 (− 0.09;0.01)	−0.03 (− 0.07;0.02)	3% (0;17)
Season (winter/ summer)	−0.04 (− 0.086;0.01)	−0.04 (− 0.09;0.01)	3% (0;17)	0.01 (− 0.04;0.06)	0.00 (− 0.05;0.05)	0% (0;10)	0.04 (− 0.01;0.08)	0.03 (− 0.02;0.08)	1% (0;5)	0.04 (− 0.01;0.09)	0.04 (− 0.01;0.09)	5% (0;19)
Special day (y/n)	0.01 (− 0.06;0.07)	0.05 (− 0.01;0.11)	1% (− 2;14)	0.04 (− 0.01;0.09)	0.05 (− 0.00;0.10)	7% (0;29)	0.05 (− 0.00;0.10)	0.10 (0.04;0.15)	5% (0;12)	0.06 (0.01;0.11)	0.08 (0.04;0.13)	**15%** (1;36)
Prior interval (hours)	− 0.02 (− 0.08;0.04)	−0.01 (− 0.08;0.05)	1% (0;14)	0.06 (0.01;0.10)	0.07 (0.03;0.11)	**14%** (1;41)	0.17 (0.12;0.21)	0.21 (0.15;0.25)	**29%** (16;43)	0.07 (0.03;0.12)	0.09 (0.04;0.13)	**20%** (3;40)
Place of meal (ref: home)												
work	−0.14 (−0.27;-0.02)	−0.15 (− 0.28;-0.02)	**41%** (1;72)	−0.14 (− 0.22;-0.06)	−0.151 (− 0.225;-0.073)	**75%** (23;90)	−0.21 (− 0.28;-0.14)	−0.26 (− 0.32;-0.19)	**45%** (24;61)	−0.08 (− 0.17;-0.01)	−0.09 (− 0.17;− 0.01)	**21%** (0;55)
restaurant	0.10 (0.06;0.15)	0.11 (0.07;0.16)	**24%** (6;50)	-0.01 (−0.06;0.05)	0.03 (−0.03;0.08)	0% (− 1;14)	0.02 (− 0.04;0.07)	0.04 (− 0.02;0.09)	1% (0;6)	0.10 (0.06;0.15)	0.12 (0.07;0.16)	**36%** (12;57)
other	0.04 (−0.00;0.09)	0.04 (−0.00;0.09)	4% (0;017)	−0.02 (− 0.07;0.04)	0.01 (− 0.04;0.06)	0% (− 1;12)	0.08 (0.03;0.13)	0.13 (0.08;0.18)	9% (2;20)	− 0.01 (− 0.08;0.06)	0.00 (− 0.07;0.07)	0% (0;17)
R-squared (95%CI)	0.05 (0.03;0.11)			0.03 (0.01;0.06)			0.12 (0.09;0.16)			0.03 (0.02;0.06)		
Participant-level covariates												
BMI (kg/m^2^)	0.00 (− 0.10;0.10)	− 0.03 (− 0.13;0.07)	0% (− 1;5)	0.08 (− 0.06;0.23)	−0.01 (− 0.14;0.12)	0% (− 1;6)	0.09 (− 0.04;0.22)	−0.04 (− 0.17;0.09)	0% (− 2;8)	0.18 (0.07;0.29)	0.09 (− 0.02;0.20)	4% (0;12)
Age (years)	0.08 (− 0.05;0.21)	0.21 (0.12;0.29)	7% (− 3;19)	0.02 (− 0.19;0.19)	0.26 (0.10;0.40)	1% (− 6;16)	− 0.10 (− 0.28;0.09)	0.02 (− 0.11;0.16)	0% (− 6;10)	− 0.22 (− 0.36;-0.07)	−0.05 (− 0.15;0.06)	2% (− 2;10)
Sex (M/W)	− 0.31 (− 0.41;-0.22)	−0.35 (− 0.43;-0.28)	**44%** (23;59)	−0.38 (− 0.57;-0.23)	−0.38 (− 0.56;-0.25)	**35%** (14;51)	−0.23 (− 0.36;-0.11)	−0.23 (− 0.35;-0.12)	**23%** (5;39)	−0.41 (− 0.50;-0.33)	−0.48 (− 0.57;-0.40)	**48%** (32;59)
Education level (ref. current/no training)												
technical college	−0.01 (− 0.10;0.08)	−0.07 (− 0.15;0.020)	0% (− 1;6)	−0.03 (− 0.16;0.11)	−0.05 (− 0.20;0.09)	0% (− 1;5)	0.08 (− 0.06;0.21)	−0.01 (− 0.14;0.11)	0% (− 2;8)	0.03 (− 0.07;0.14)	−0.10 (− 0.20;-0.00)	0% (− 2;3)
university	− 0.01 (− 0.11;0.08)	0.06 (− 0.03;0.14)	0% (− 1;4)	− 0.08 (− 0.23;0.06)	−0.02 (− 0.15;0.13)	0% (− 1;6)	0.04 (− 0.10;0.18)	0.08 (− 0.05;0.20)	1% (− 1;11)	0.08 (− 0.04;0.19)	0.15 (0.05;0.26)	3% (− 1;11)
Occupation (ref. no job/ retired)^f^												
full time	0.05 (− 0.11;0.20)	− 0.05 (− 0.15;0.06)	0% (− 3;6)	−0.033 (− 0.233;0.177)	−0.166 (− 0.359;0.013)	1% (− 3;14)	0.05 (− 0.16;0.26)	0.02 (− 0.14;0.17)	1% (− 2;13)	−0.00 (− 0.15;0.14)	0.07 (− 0.04;0.18)	0% (− 2;4)
part time/hourly	−0.08 (− 0.19;0.04)	−0.17 (− 0.28;-0.05)	5% (− 1;17)	0.017 (− 0.15;0.175)	−0.061 (− 0.225;0.095)	0% (−1;7)	0.03 (− 0.08;0.15)	−0.00 (− 0.12;0.10)	0% (− 1;6)	0.05 (− 0.05;0.15)	0.02 (− 0.09;0.12)	0% (0;4)
Physical activity (h/week)	0.05 (− 0.03;0.13)	0.05 (− 0.03;0.12)	1% (0;6)	0.145 (0.013;0.280)	0.162 (0.041;0.290)	6% (0;14)	0.02 (− 0.09;0.12)	− 0.01 (− 0.12;0.10)	0% (0;5)	0.05 (− 0.04;0.13)	−0.04 (− 0.13;0.06)	0% (−1;2)
Smoking status (ref. never smoker)												
current smoker	0.27 (0.06;0.45)	0.04 (−0.05;0.13)	4% (−4;17)	0.186 (−0.064;0.449)	0.076 (− 0.057;0.202)	3% (−1;15)	0.18 (− 0.03;0.40)	0.12 (0.00;0.24)	9% (−1;29)	0.03 (− 0.17;0.23)	−0.17 (− 0.27;-0.07)	0% (− 7;9)
former smoker	0.20 (0.01;0.40)	0.06 (− 0.03;0.15)	5% (− 1;18)	0.067 (− 0.187;0.351)	−0.014 (− 0.143;0.130)	0% (− 2;7)	0.02 (− 0.21;0.24)	−0.08 (− 0.20;0.04)	0% (−6;12)	0.09 (− 0.10;0.28)	0.18 (0.07;0.28)	4% (− 3;15)
Energy misreporting												
EI/TEE < 0.81	−0.15 (− 0.25;-0.07)	−0.26 (− 0.35;-0.18)	**16%** (5;31)	−0.372 (− 0.539;-0.227)	−0.462 (− 0.603;-0.343)	**41%** (18;58)	−0.37 (− 0.52;-0.25)	−0.38 (− 0.53;-0.26)	**61%** (30;74)	−0.30 (− 0.42;-0.19)	−0.31 (− 0.41;-0.21)	**23%** (10;35)
EI/TEE > 1.19	0.18 (0.11;0.25)	0.27 (0.20;0.33)	**19%** (8;30)	0.159 (0.058;0.278)	0.311 (0.212;0.440)	**12%** (3;22)	0.10 (−0.03;0.23)	0.19 (0.05;0.32)	8% (−1;24)	0.24 (0.16;0.31)	0.31 (0.23;0.38)	**18%** (9;27)
R-squared (95%CI)	0.25 (0.20;0.36)			0.42 (0.31;0.78)			0.23 (0.16;0.44)			0.41 (0.34;0.56)		

^a^n = 682 participants with activity sensor data

^b^Pratt Index, in % contribution to the variance explained by the model (R^2^). Might not add up to 100% due to rounding errors from parameter estimates

^c^for dichotomous variables, the information shown is for the underlined category (reference category not underlined)

^d^all 95% confidence intervals (95%CI) – for beta-weights, correlations, r-squared, and Pratt Index – are bootstrap confidence intervals based on 1000 samples

^e^bold numbers indicate covariates accounting for > 10% of the explained variance

^f^full time: > 35 h/week; part time/hourly: < 35 h/week

#### Energy

##### Intake-level predictors

The workplace as the *place of meal* was the most important predictor for energy intake, predicting a lower intake than at home during breakfast, lunch, and afternoon snack, accounting for 45, 60, and 43% of the explained variance, respectively (Table 3), although bootstrap confidence intervals were broad, indicating a higher degree of uncertainty. At dinner, restaurant as the *place of meal* was the most important intake-level predictor, predicting a higher intake and accounting for 43% of the explained variance. *Week/weekend day* seems to be an important predictor of the explained variance with a higher intake during weekends at breakfast, accounting for 24% of the explained variance but was less important during the meals later in the day (10% at lunch, 12% at the afternoon snack, and 1% at dinner). *Duration of prior interval* was an important predictor at the afternoon snack (27%) and dinner (17%), predicting higher energy intake. *Special day* accounted for 10 and 16% of the intake-level explained variance for energy intake at lunch and dinner, respectively, predicting a higher energy intake. *Season* did not account for much of the explained variance of energy intake in any of the meals (0–4%). The model fit (total standardized variance explained by the model) was as follows: R^2^_breakfast_ = 0.044, R^2^_lunch_ = 0.023, R^2^_afternoon snack_ = 0.106, and R^2^_dinner_ = 0.030 (Table 3).

##### Participant-level predictors

*Sex* was consistently the main predictor of the explained variance for all meal types, predicting a lower intake in women and having the lowest relative importance for breakfast with 64% and highest for dinner with 90% (Table 3). Current *smoking* accounted for 25% of the participant level explained variance for energy intake at the afternoon snack and predicted a higher intake in current smokers versus never smokers. Age accounted for 17 and 12% of the explained variance at breakfast and at lunch, respectively, predicting a higher intake at a higher age. Neither *education level*, *current occupation*, nor *physical activity* were important predictors of energy intake in the participant-level. The model fit was R^2^_breakfast_ = 0.179, R^2^_lunch_ = 0.276, R^2^_afternoon snack_ = 0.072, and R^2^_dinner_ = 0.282 (Table 3).

##### Sensitivity analysis

The sensitivity analysis with the models adjusted for under- (EI/TEE < 0.81) and over-reporting (EI/TEE > 1.19) of energy are shown in Table 4. Results in the intake level were not different from the main results, except for a 15% increase in the explained variance for work place at lunch (inverse association), but as in the main analysis, bootstrap confidence intervals were broad for this parameter estimate. At the participant level, *energy misreporting* accounted for 35 to 69% of the explained variance, being lowest at breakfast and highest at afternoon snack; bootstrap confidence intervals for were in general narrow, indicating little uncertainty for the relative importance of these covariates. Because of the importance of *energy misreporting*, the proportions of explained variance by the other factors were reduced; sex accounted for 23% at afternoon snack (lower intake by women). The importance of current smoking at afternoon snack also dropped from 25 to 9%. In general, the participant-level model fits were greater in the sensitivity analysis compared to the main results: R^2^_breakfast_ = 0.250, R^2^_lunch_ = 0.415, R^2^_afternoon snack_ = 0.231, and R^2^_dinner_ = 0.410 (Table 4).

#### Carbohydrates

##### Intake-level predictors

For carbohydrates, *place of meal* (workplace) was the intake-level covariate accounting for most of the explained variance for breakfast, lunch, and afternoon snack with 65, 34, and 40%, respectively, predicting a lower carbohydrate intake at work than at home (Additional file 1: Table S3), although characterized by broad bootstrap confidence intervals. The other *places of meal* accounted for a low amount of the explained variance in comparison (restaurant: 13 and 12% at breakfast and lunch, respectively; other: 11% at afternoon snack). *Prior interval* was the most important intake-level covariate for dinner, accounting for 50% (but with a broad bootstrap confidence interval (95% bootstrap CI) of 5–76%) of the explained variance and was the second most important covariate for afternoon snack, accounting for 30% of the explained variance (positive associations). However, *prior interval* was not relevant for breakfast or lunch. *Special day* was an important predictor of carbohydrate intake at dinner, accounting for 29% (95% bootstrap CI: 1;60) of the explained variance in this level and predicting a higher carbohydrate intake on special days; it also accounted for 9 and 5% of the explained variance at lunch and afternoon snack, respectively. *Season* accounted for 22% (95% bootstrap CI: 0;62) of the explained variance at lunch and for 10% at breakfast (lower carbohydrate intake in the summer). Whether the intake took place on a *weekday or weekend day* accounted for 13% at breakfast, 12% at lunch, and 11% at afternoon snack and was predicted to be greater in the weekend; at lunch, there was higher uncertainty about the parameter estimate PI (95% bootstrap CI: 0;54). The models had fits of R^2^_breakfast_ = 0.021, R^2^_lunch_ = 0.005, R^2^_afternoon snack_ = 0.065, and R^2^_dinner_ = 0.010 (Additional file 1: Table S3).

##### Participant-level predictors

*Sex* was the most important predictor of carbohydrate intake, accounting 41, 65, 68, and 95% of the explained variance at breakfast, lunch, afternoon snack, and dinner, respectively (lower intake by women) (Additional file 1: Table S3). At lunch and afternoon snack, bootstrap confidence intervals were broad, indicating less uncertainty about the parameter estimates for PI. *Age* accounted for 14 and 12% of the explained variance at breakfast and lunch, respectively, predicting a higher intake at higher ages. *BMI* accounted only for 8% of the explained variance at breakfast and 4% at lunch. Neither *education level*, nor *physical activity* were important predictors of carbohydrate intake at any of the meals. *Current occupation* accounted for 8% of the explained variance for part time/hourly jobs at breakfast, but did not account for much of the variance in the other meals or categories. Finally, current *smoking* was an important predictor at breakfast, lunch, and afternoon snack, accounting for 21, 13, and 33% of the explained variance for carbohydrate intake in the participant level and predicting a higher carbohydrate intake in current smokers than in never smokers, although the broad bootstrap confidence interval for current smoking at afternoon snack indicates less certainty for this estimate. Model fits were R^2^_breakfast_ = 0.172, R^2^_lunch_ = 0.253, R^2^_afternoon snack_ = 0.067, and R^2^_dinner_ = 0.203 (Additional file 1: Table S3).

##### Sensitivity analysis

In the intake level, most results remained substantially unchanged. However, at lunch, the relative importance of *week/weekend day* decreased to 2% and that of *place of meal* increased for all categories (43% for work, 20% for restaurant, and 9% for other) (Additional file 1: Table S4). The importance of the participant-level covariates decreased proportionally with the high impact of *energy misreporting* on the explained variance in this level; energy misreporting accounted for 27–65% of the explained variance, being lowest at breakfast and highest at afternoon snack. However, uncertainty in the parameter estimate PI was high for energy underreporting (EI/TEE < 0.81) at afternoon snack (95% bootstrap CI: 12;71). *Sex* remained a very important predictor, accounting for 28% of the explained variance at breakfast, 38% at lunch, 26% at afternoon snack, and 46% at dinner. The importance of *age* and *BMI* was reduced. Current *smoking* remained an important predictor at breakfast (20%). The participant-level model fits were improved compared to the main analysis: R^2^_breakfast_ = 0.223, R^2^_lunch_ = 0.382, R^2^_afternoon snack_ = 0.201, and R^2^_dinner_ = 0.310 (Additional file 1: Table S4).

#### Protein

##### Intake-level predictors

Restaurant as a *place of meal* was the most important predictor of the explained variance in protein intake in this level at breakfast and dinner, accounting for 40 and 51% of the explained variance, respectively and predicting a higher protein intake in restaurants than at home (Additional file 1: Table S3) (but with higher uncertainty at dinner, with 95% bootstrap CI: 15;71). The workplace was the most important predictor at lunch and afternoon snack, accounting for 59 and 41% of the explained variance, respectively and predicting a lower intake than at home (but with higher uncertainty at lunch, with 95% bootstrap CI: 28;79). *Week/weekend day* accounted for 35% of the explained variance at breakfast and 17% at lunch (higher intake for the weekend), but not much at afternoon snack or dinner. *Special day* accounted for 15% of the explained variance for protein intake at dinner and for 10% at afternoon snack (higher protein intake on special days). *Season* did not account for an important part of the explained variance for any of the meal types. The *prior interval* was an important predictor at afternoon snack and dinner, accounting for 34 and 13% of the explained variance, respectively (positive association). The model fits were as follows: R^2^_breakfast_ = 0.048, R^2^_lunch_ = 0.042, R^2^_afternoon snack_ = 0.074, and R^2^_dinner_ = 0.023 (Additional file 1: Table S3).

##### Participant-level predictors

*Sex* was the most important predictor at breakfast, lunch, and dinner, accounting for 71% of the explained variance at breakfast, 74% at lunch, and 68% at dinner, predicting lower intake by women than by men (Additional file 1: Table S3) but was characterized by broader bootstrap confidence intervals, especially at breakfast, lunch, and afternoon snack. At the afternoon snack, *sex* accounted for 32% of the explained variance and was second in importance to the full time *current occupation*, which accounted for 53% of the explained variance (higher protein intake by full-time employed than retired/not employed) but characterized also by a broad bootstrap confidence interval (95% bootstrap CI: -4;78). A full time *current occupation* accounted also for 9% of the explained variance at lunch, but was unimportant in the other meals and categories. University level education accounted for 9% of the explained variance at dinner but *education level* was unimportant for the other meals, as was also the case for *physical activity*, which did not have an important impact on any of the meals. *BMI* was an important predictor for explained variance in protein intake at dinner but not at the other meals, accounting for 19% of the explained variance (positive association). *Age* and *smoking status* accounted each for 6–9% at breakfast, lunch, and afternoon snack. Variance explained by the participant level part of the models (model fit) was R^2^_breakfast_ = 0.102, R^2^_lunch_ = 0.212, R^2^_afternoon snack_ = 0.033, and R^2^_dinner_ = 0.253 (Additional file 1: Table S3).

##### Sensitivity analysis

At lunch, workplace increased 10% and other place decreased 11%. At dinner, *season* increased 6% while restaurant as the *place of meal* decreased 12% in the relative importance as predictors of protein intake (Additional file 1: Table S4). However, most of the changes were seen in the participant level, where *energy misreporting* was added as a covariate; reporting of energy intake below the total energy expenditure (EI/TEE < 0.081, indicative of underreporting) accounted for an important proportion of the variance and was greatest at lunch, followed by afternoon snack (40 and 34%, respectively). However, at these two meals, bootstrap confidence intervals for this parameter were broad (95% bootstrap CI 13;60 and 3;58, for lunch and afternoon snack, respectively). In general, *energy misreporting* (over- and underreporting) accounted for 30% at breakfast, 56% at lunch, 51% at afternoon snack, and 32% at dinner. Proportionally, the importance of the other participant-level covariates decreased; *sex* remained an important predictor but decreased in importance by 18% at breakfast, 44% at lunch, 23% at afternoon snack, and 27% at dinner. *BMI* remained an important predictor for protein intake at dinner, accounting for 15% of the explained participant-level variance. *Age* and current smoking, however, decreased in importance, especially at breakfast and lunch. A full time *current occupation* was still an important predictor of protein intake at afternoon snack, accounting for 22% of the explained variance. Participant-level model fits were better than those of the main analysis, with R^2^_breakfast_ = 0.127, R^2^_lunch_ = 0.362, R^2^_afternoon snack_ = 0.118, and R^2^_dinner_ = 0.345 (Additional file 1: Table S4).

#### Fat

##### Intake-level predictors

Restaurant as the *place of meal* was the most important predictor of explained variance in fat intake at breakfast, with 37%, and the second most important predictor at dinner, with 27% and being higher for restaurant meals than home meals (Additional file 1: Table S3). Workplace was the most important predictor at lunch, afternoon snack, and dinner, accounting for 38, 45, and 39%, respectively, predicting lower fat intake at work than at home. Other meal place accounted for 22 and 10% of the explained variance at lunch and afternoon snack, respectively, predicting a lower fat intake at lunch but higher fat intake at the afternoon snack, respectively. Similar to the other macronutrients, uncertainty in the parameter estimates for PI was higher for meals at work and at restaurants (at dinner only), indicated by broad bootstrap confidence intervals. *Week/weekend day* accounted for 32% of the intake-level explained variance in fat intake at breakfast and for 18% at lunch (higher intake for weekend). *Prior interval* accounted for 25% of the explained variance at afternoon snack (positive association). *Special day* accounted for 9% of the explained variance at lunch, afternoon snack, and dinner. *Season* was an important predictor of fat intake only at dinner, accounting for 10% of the explained variance, predicting a higher intake in the summer. The model fits for the intake level part of the models were R^2^_breakfast_ = 0.046, R^2^_lunch_ = 0.022, R^2^_afternoon snack_ = 0.083, and R^2^_dinner_ = 0.014 (Additional file 1: Table S3).

##### Participant-level predictors

*Sex* was the most important participant level predictor of fat intake, accounting for 86% of the explained variance at breakfast, 54% at lunch, 63% at afternoon snack, and 80% at dinner (lower fat intake by women) (Additional file 1: Table S3). Like in the case of the other macronutrients, bootstrap confidence intervals were broader for *sex* at lunch and afternoon snack (95% bootstrap CI: 18:71 and 9;77, respectively). All other covariates were relatively unimportant with the exceptions of *age* at lunch, accounting for 36% of the explained variance (positive association) and former *smoking* at the afternoon snack, accounting for 29% of the explained variance (lower fat intake by former smokers than never smokers), although bootstrap confidence interval for the latter was broad. The fit for the participant level part of these models were R^2^_breakfast_ = 0.120, R^2^_lunch_ = 0.220, R^2^_afternoon snack_ = 0.063, and R^2^_dinner_ = 0.220 (Additional file 1: Table S3).

##### Sensitivity analysis

Results were mostly consistent in the intake level, with slight changes at lunch: the relative importance of *week/weekend day* and *prior interval* increased by 9% each, while it decreased by 16% for other *place of meal* (Additional file 1: Table S4). As for the participant level part of the model, *energy misreporting* accounted for 38–59% of the explained variance, being smallest at breakfast and greatest at afternoon snack. Reporting lower energy intake than the energy expenditure (EI/TEE < 0.81) was greatest at lunch, accounting for 42% of the explained variance in this level, but with a broad bootstrap confidence interval (95% bootstrap CI: 15;61), indicating a higher degree of uncertainty in this estimate. The importance of the other participant-level covariates decreased proportionally, with *sex* now accounting for 59, 21, 26, and 37% of the explained variance in fat intake at breakfast, lunch, afternoon snack, and dinner, respectively. *Age* was still an important predictor at lunch, accounting for 11% of the explained variance and current *smoking* was also still an important predictor at afternoon snack, accounting for 12% of the explained variance in fat intake. The participant-level model fits were improved in the sensitivity analysis: R^2^_breakfast_ = 0.157, R^2^_lunch_ = 0.356, R^2^_afternoon snack_ = 0.167, and R^2^_dinner_ = 0.380 (Additional file 1: Table S4).

## Discussion

This study showed that meal type (breakfast, lunch, afternoon snack, and dinner), together with specific intake occasions, is a very important source of variation in energy and macronutrient intake. In comparison to these sources, the variation between participants in respect to meals in general is very small and it concentrates either on individual preferences regarding carbohydrates or fat. The most important predictors of variance at the first intake level with respect to energy and macronutrient intake were *place of meal*, *week/weekend day,* and *prior interval* and at the participant level *sex*, but also other variables played a role, sometimes concentrating on a certain meal and a macronutrient. However, predictors could only explain, on average, a small part of the variation within the type of meal and broad confidence intervals indicated uncertainty about the importance of the covariate *place of meal*. Also, *energy misreporting* seems to play an important role in predicting variance in meal type, especially for afternoon snacks in respect to energy and carbohydrate intake. The results suggest that the context of a meal determines energy and macronutrient intake; therefore, efforts to change energy and macronutrient intake should consider such contexts.

In the past few decades, the interest in diet has been mostly concentrating on usual/habitual diet, which in principle is averaging out the differences in the eating occasions or meals. However, eating occasions and meals are the building blocks of dietary intake and they vary considerably within a day and across days. Therefore, it was not surprising to find large variation of energy and macronutrient intake across the meals. Our results are specific to our study population and the cultural context, but can be discussed and compared to results from other studies looking into meal-specific or intake-level factors that potentially affect dietary intake. For example, de Castro and colleagues [29] looked into context and psychological variables at meals and found number of people and hunger to be related to meal size. In our study, no information about the number of people or the hunger level of participants was available; however, duration of prior interval is highly correlated with hunger. Our results showed that *prior interval* was a predictor of energy and carbohydrate intake in the afternoon snack and dinner but not in breakfast and lunch, which is consistent with the results of de Castro et al., as they reported larger meal sizes with longer after-meal intervals in the afternoon and evening. Therefore, a late breakfast and a long interval until lunch together with a short time until afternoon snack and an early dinner could potentially result in reduced dietary intake and successful weight loss. A recent randomized study on type 2 diabetes patients found that two larger meals (breakfast and lunch) were a better approach for body weight and type 2 diabetes control than 6 small meals per day [30]. As for the influence of the place of meal on dietary intake, a systematic review states that eating out of home is associated with higher total energy intake and higher contribution of fat [4]. This was also true in our results for meals at restaurants, which we observed as well for protein intake. The predictor meal at work, however, pointed in our study to a lower energy and macronutrient intake.

In our study, the day of the week (*weekday* vs. *weekend day*) contributed to the explained variance for energy and all three macronutrients at breakfast and lunch (higher intake on weekends), but less so for afternoon snack and dinner. A study examining differences in nutrient intake and dietary quality in a Canadian population found overall a higher intake of energy in weekends traceable to higher consumption of fat and alcohol and a lower intake of carbohydrates and proteins [31]. Such discrepancies with our results might be due to differences in the population and cultural context. Regarding the *season*, we observed a contribution to the explained variance for carbohydrate intake at breakfast and lunch and for fat intake at dinner. The comparable small contribution of season could be the effect of a high socioeconomic level of this population. The contribution might be higher in a more heterogeneous population including more participants of lower socioeconomic levels. de Castro et al. observed a peak in daily intake (11–14% higher) in autumn than in the other seasons [29]. Other studies point to a higher intake in winter: a recent systematic review and meta-analysis on seasonality of food groups and total energy intake found winter to be associated with a higher energy intake [32]. Similarly, another study on an adult polish population found food energy density to be greatest in autumn/winter and lowest in spring/summer [33].

As for participant-level predictors, a study investigating the impact of different environments on fat intake among rural residents in the U. S found that age (participants aged 40–70), sex, and self-efficacy for healthy eating were associated with fat intake while education was not related [3], however, this study made no distinction between meals. Our results were generally consistent in that *sex* was an important predictor, as well as *age* at selected meals, and in that *education level* was not related to dietary intake. A study on personality and situation predictors of consistent eating patterns (and therefore lower variability of intake) looked at person-related and environment-related variables and also found that place of meal and time of meal (evening) were predictors of eating consistency, along with physical activity and self-control [34]. In our study, however, *physical activity* did not play an important role in predicting dietary intake. While we found a higher intake of carbohydrates in current *smokers* vs never smokers (at breakfast, lunch, and afternoon snack), a previous study across all EPIC study centers (10 European countries) observed a lower carbohydrate consumption in some study centers [35], but not for the EPIC-Potsdam population, of which our study population is a sub-cohort. Finally, we were able to show in the sensitivity analyses that during the afternoon snacks, differences between individuals’ energy and macronutrient intake reporting were greatly due to energy underreporting. A recent study in the same study population found that energy misreporting had a great impact on intake of cakes and cookies, a food group often consumed by Germans during the afternoon time [26]. Besides conscious underreporting in this meal, afternoon snack might be a challenging meal to report accurately (affecting both over- and under-reporting), for example due to fewer attentional resources for a conscientious intake [34].

Our study has several strengths. We were able to show the importance of the different types of meal in terms of the variability in energy and macronutrient intake, as well as the relative importance of some intake-level (within-person) and participant-level (between-person) predictors. For the latter, we used the Pratt Index (PI), a method to find the importance of covariates in a level-specific hierarchical model in terms of the explained variance. The order of importance of the covariates is not the same as it would be by looking at the beta-weight (effect estimate), correlation, or t-test alone, as the PI weighs the effects of the covariates by the explained variance, summing to 1 (additive property). The additive property and simplicity in interpretation makes PI a convenient tool for variable importance in contrast to alternate methods like beta-weights and partial correlation, which lack proportionality and additive properties [36–38]: This method is easily applied on *MPlus* in a SEM framework and cannot be used with other techniques commonly applied, such as hierarchical linear model analysis, where there is no R^2^ measure that can be partitioned additively and into within- and between-level covariance matrices [23]. While the PI may have its limitations, such as being only applicable to models with random intercepts (but not slopes) [23], and that like other statistical tools is prone to bias and other sources of error, we found this method to describe the relative importance of covariates in the most comprehensive way. In addition, it must be noted that PI shows only statistical importance of the variable in the model; therefore, researchers should consider the importance of the variables within context [37, 38]. Another strength of this study is the availability of multiple 24hDR on random days. An advantage of short-term dietary assessment methods such as the 24hDR is that they provide more detailed information about types and amounts of food consumed and they are typically meal-specific, allowing meal-based analyses. These methods imply a larger within-person variation of dietary estimates [8]. A minimum of two 24hDRs are needed to separate the within and between variability and 3–4 records to achieve modest precision of dietary intake [5, 9]. However, the administration of 4–6 24hDRs are recommended for a more precise estimation, especially in the case of episodically consumed foods [39].

Our study also has a few limitations. First, a general limitation in the field of nutritional epidemiology is measurement error; although EPIC-SOFT (renamed GloboDiet in 2014) is a validated and standardized tool, the 24hDR, like any dietary assessment method, is not free of error [40]. An effort for addressing this problem is assessing diet using validated and standardized methods such as the EPIC-SOFT program used for the present study. Another limitation is that we restricted our analyses to the four meals with peaks of consumption. Only for the calculation of prior interval were all 11 eating occasions considered. Therefore, all snacks and smaller meals were not considered in the intakes. Also, the model fit (R^2^) for the calculation of the relative importance of predictors was low, even if many covariates were included. This phenomenon has been addressed before [29]. In real-world settings, the variance is often very large in comparison to that in the laboratory, where some factors that influence dietary intake are not present due to standardization. Therefore, in the case of variance in meals, many factors, which are not yet fully understood, seem to play a role. Another limitation is that uncertainty about parameter estimates was at times very large, especially for place of meal, since the frequency of out-of-home meals was often low. We included known factors influencing meal intake in our models. Other predictors that were not available in our dataset, such as number of people present during the meal and personality traits, could have improved the models. Chronic disease and medication use were also not included in our models, as health status would be a complicated factor to study in the context of a generally healthy study population and cross-sectional study design.

## Conclusions

In conclusion, our study shows that a great proportion of the variance in energy and macronutrient intake is due to differences between meal types. The study further provides a deeper insight into the predictors of dietary intake for each type of meal. These findings suggest that meals could be an important intervention target in dietary modification. Further studies are required to validate these results and ascertain important predictors of both participant- and intake-level variation in dietary intake that could be used for dietary modification.

## Additional files

Additional file 1: **Box 1.** Additional statistical methods: *ICC* calculation. **Box 2.** Additional statistical methods: *Selection and description of covariates*. **Box 3.** Additional statistical methods: *R*^*2*^*and Pratt Index*. **Box 4.** Additional statistical methods: *Main models*. **Box 5.** Additional statistical methods: *95% bootstrap confidence intervals.* **Figure S1.** Flow diagram of participants of the validation sub-study within the EPIC-Potsdam cohort. **Table S1.** Eating occasions with participant-identified labels used to record food intake in the 24hDR. **Table S2.** Number of 24hDR and meals, total and by participant. **Table S3.** Relative importance of predictors of macronutrient intake (g/meal), Pratt Index overview. **Table S4.** Relative importance of predictors of macronutrient intake (g/meal), Pratt Index overview; sensitivity analysis adjusting for energy misreporting. **Table S5.** Random intercept Multilevel Regression Analysis and Corresponding Pratt for energy intake (kcal/meal). **Table S6.** Random intercept Multilevel Regression Analysis and Corresponding Pratt for carbohydrate intake (g/meal). **Table S7.** Random intercept Multilevel Regression Analysis and Corresponding Pratt for protein intake (g/meal). **Table S8.** Random intercept Multilevel Regression Analysis and Corresponding Pratt for fat intake (g/meal). **Table S9.** Random intercept Multilevel Regression Analysis and Corresponding Pratt for energy intake (kcal/meal); sensitivity analysis adjusting for energy misreporting. **Table S10.** Random intercept Multilevel Regression Analysis and Corresponding Pratt for carbohydrate intake (g/meal); sensitivity analysis adjusting for energy misreporting. **Table S11.** Random intercept Multilevel Regression Analysis and Corresponding Pratt for protein intake (g/meal); sensitivity analysis adjusting for energy misreporting. **Table S12.** Random intercept Multilevel Regression Analysis and Corresponding Pratt for fat intake (g/meal); sensitivity analysis adjusting for energy misreporting (DOCX 414 kb)

Additional file 2: STROBE-nut checklist (DOCX 36 kb)

## Abbreviations

24hDR: 24-h dietary recall
ICC: intra-class correlation coefficient
PI: Pratt Index
SEM: structural equation modeling
