# Correction to: Meal analysis for understanding eating behavior: meal- and participant-specific predictors for the variance in energy and macronutrient intake

**DOI:** 10.1186/s12937-019-0453-3

**Published:** 2019-04-25

**Authors:** Carolina Schwedhelm, Khalid Iqbal, Lukas Schwingshackl, George O. Agogo, Heiner Boeing, Sven Knüppel

**Affiliations:** 1 Department of Epidemiology, German Institute of Human Nutrition Potsdam-Rehbruecke (DIfE), Arthur-Scheunert-Allee 114-116, 14558 Nuthetal, Germany; 2 NutriAct – Competence Cluster Nutrition Research, Berlin-Potsdam, Germany; 3 Institute for Evidence in Medicine, Faculty of Medicine and Medical Center, University of Freiburg, Freiburg, Germany; 4 Department of Internal Medicine, Yale School of Medicine, New Haven, CT USA; 5 Department of Nutrition and Gerontology, German Institute of Human Nutrition Potsdam-Rehbruecke (DIfE), Arthur-Scheunert-Allee 114-116, 14558 Nuthetal, Germany

**Correction to: Nutr J**

**https://doi.org/10.1186/s12937-019-0440-8**

Following publication of the original article [1], the authors reported an error in Table 3. The correct Table 3 is provided below.

**Table 3:** Relative importance of predictors of energy intake (kcal/meal)^a^

Covariates^b^	Breakfast			Lunch			Afternoon snack			Dinner		
	Beta-weight (95%CI)^c^	Correlation (95%CI)	Pratt Index (95%CI)	Beta-weight (95%CI)	Correlation (95%CI)	Pratt Index (95%CI)	Beta-weight (95%CI)	Correlation (95%CI)	Pratt Index (95%CI)	Beta-weight (95%CI)	Correlation (95%CI)	Pratt Index (95%CI)
Intake-level covariates												
Week/weekend day (y/n)	0.10 (0.04;0.14)	0.11 (0.06;0.16)	**24%**^d^(5;46)	0.04 (− 0.01;0.09)	0.06 (0.01;0.10)	**10%** (0;33)	0.10 (0.05;0.15)	0.13 (0.08;0.17)	**12%** (4;22)	−0.02 (− 0.07;0.02)	−0.02 (− 0.06;0.03)	1% (0;12)
Season (winter/ summer)	−0.04 (− 0.08;0.01)	−0.04 (− 0.08;0.00)	3% (0;16)	0.01 (− 0.04;0.05)	− 0.01 (− 0.05;0.04)	0% (0;11)	0.03 (− 0.02;0.07)	0.02 (− 0.02;0.07)	1% (0;4)	0.03 (− 0.01;0.07)	0.03 (− 0.01;0.08)	4% (0;16)
Special day (y/n)	0.00 (− 0.06;0.06)	0.03 (− 0.03;0.09)	0% (− 1;10)	0.04 (0.00;0.09)	0.05 (0.00;0.010)	**10%** (0;34)	0.07 (0.02;0.12)	0.11 (0.06;0.16)	7% (1;16)	0.06 (0.01;0.10)	0.09 (0.04;0.13)	**16%** (1;38)
Prior interval (hours)	−0.02 (− 0.08;0.04)	− 0.01 (− 0.07;0.05)	1% (0;12)	0.04 (0.00;0.08)	0.05 (0.01;0.09)	**10%** (0;34)	0.15 (0.11;0.120)	0.19 (0.14;0.23)	**27%** (15;40)	0.07 (0.02;0.11)	0.08 (0.04;0.12)	**17%** (3;36)
Place of meal (ref: home)												
Work	−0.14 (− 0.24;-0.04)	− 0.14 (− 0.25;-0.04)	**45%** (4;73)	−0.12 (− 0.19;-0.04)	−0.12 (− 0.19;-0.04)	**60%** (10;85)	−0.19 (− 0.25;-0.13)	−0.24 (− 0.30;-0.18)	**43%** (24;60)	− 0.07 (− 0.14;-0.00)	−0.08 (− 0.15;-0.01)	**18%** (0;48)
Restaurant	0.11 (0.06;0.15)	0.11 (0.07;0.15)	**27%** (8;54)	0.02 (− 0.03;0.07)	0.05 (0.00;0.09)	4% (1;29)	0.02 (−0.03;0.07)	0.04 (−0.01;0.09)	1% (0;5)	0.11 (0.06;0.15)	0.12 (0.08;0.16)	**43%** (17;64)
Other	0.01 (−0.06;0.07)	0.01 (− 0.06;0.07)	0% (0;13)	−0.05 (− 0.10;0.00)	−0.03 (− 0.08;0.02)	6% (0;31)	0.08 (0.03;0.13)	0.13 (0.08;0.18)	**10%** (3;20)	0.01 (− 0.05;0.07)	0.02 (− 0.04;0.08)	1% (0;17)
R-squared (95%CI)	0.04 (0.03;0.09)			0.02 (0.01;0.05)			0.11 (0.08;0.14)			0.03 (0.02;0.05)		
Participant-level covariates												
BMI (kg/m^2^)	−0.08 (− 0.18;0.00)	−0.07 (− 0.16;0.02)	3% (0;14)	− 0.07 (− 0.21;0.06)	−0.04 (− 0.19;0.10)	1% (0;12)	− 0.03 (− 0.17;0.08)	− 0.04 (− 0.18;0.07)	2% (0;24)	0.08 (− 0.02;0.17)	0.09 (− 0.01;0.18)	2% (0;9)
Age (years)	0.15 (0.02;0.26)	0.21 (0.13;0.30)	**17%** (2;33)	0.12 (− 0.07;0.30)	0.28 (0.13;0.43)	**12%** (−4;33)	− 0.01 (− 0.18;0.18)	0.04 (− 0.09;0.17)	0% (− 7;24)	− 0.08 (− 0.22;0.07)	−0.03 (− 0.13;0.09)	1% (− 1;8)
Sex (M/W)	− 0.34 (− 0.43;-0.25)	−0.34 (− 0.41;-0.26)	**64%** (39;80)	−0.46 (− 0.65;-0.31)	−0.40 (− 0.56;-0.27)	**66%** (34;82)	− 0.26 (− 0.39;-0.14)	− 0.21 (− 0.31;-0.10)	**76%** (17;86)	−0.51 (− 0.58;-0.43)	−0.50 (− 0.57;-0.44)	**90%** (72;95)
Education level (ref. current/no training)												
Technical college	−0.01 (− 0.11;0.09)	−0.07 (− 0.15;0.02)	0% (− 1;8)	− 0.03 (− 0.17;0.09)	−0.04 (− 0.17;0.08)	1% (− 1;8)	0.03 (− 0.10;0.15)	− 0.01 (− 0.13;0.10)	0% (− 2;16)	− 0.04 (− 0.15;0.07)	− 0.15 (− 0.25;-0.05)	2% (− 2;12)
University	− 0.01 (− 0.11;0.08)	0.06 (− 0.02;0.14)	0% (− 2;6)	− 0.10 (− 0.25;0.03)	−0.02 (− 0.16;0.11)	1% (− 3;10)	−0.02 (− 0.15;0.13)	0.02 (− 0.10;0.14)	0% (− 2;18)	0.03 (− 0.07;0.14)	0.15 (0.06;0.26)	2% (− 2;11)
Occupation (ref. no job/ retired)^e^												
Full time	0.02 (− 0.11;0.16)	− 0.06 (− 0.16;0.03)	0% (− 3;8)	−0.09 (− 0.30;0.10)	− 0.19 (− 0.38;-0.02)	6% (−3;28)	0.01 (− 0.17;0.22)	0.01 (− 0.13;0.15)	0% (− 4;26)	0.02 (− 0.12;0.15)	0.08 (− 0.02;0.18)	1% (− 2;8)
Part time/hourly	− 0.06 (− 0.16;0.03)	− 0.14 (− 0.24;-0.04)	5% (− 1;18)	− 0.00 (− 0.17;0.16)	−0.08 (− 0.23;0.07)	0% (− 1;12)	−0.01 (− 0.13;0.10)	−0.05 (− 0.17;0.06)	1% (− 2;20)	0.09 (− 0.01;0.19)	0.03 (− 0.07;0.12)	1% (− 1;7)
Physical activity (h/week)	0.04 (− 0.04;0.12)	0.04 (− 0.03;0.12)	1% (0;7)	0.14 (0.00;0.28)	0.15 (0.02;0.27)	7% (0;19)	0.05 (− 0.05;0.15)	0.02 (− 0.08;0.13)	2% (− 1;17)	0.07 (− 0.02;0.16)	−0.03 (− 0.13;0.05)	0% (− 2;3)
Smoking status (ref. never smoker)												
Current smoker	0.25 (0.08;0.42)	0.04 (−0.04;0.12)	5% (−4;19)	0.23 (−0.00;0.48)	0.05 (− 0.08;0.16)	4% (− 4;20)	0.20 (− 0.04;0.42)	0.09 (− 0.03;0.21)	**25%** (− 3;62)	0.05 (− 0.13;0.24)	−0.17 (− 0.27;-0.07)	0% (− 11;9)
Former smoker	0.18 (0.01;0.35)	0.06 (− 0.03;0.14)	6% (− 1;20)	0.12 (− 0.12;0.38)	0.03 (− 0.09;0.16)	1% (− 2;15)	0.04 (− 0.18;0.25)	−0.05 (− 0.17;0.07)	0% (− 12;22)	0.08 (− 0.10;0.27)	0.18 (0.08;0.28)	5% (−4;20)
R-squared (95%CI)	0.18 (0.13;0.26)			0.28 (0.18;0.54)			0.07 (0.05;0.19)			0.28 (0.23;0.39)		

^a^Pratt Index, in % contribution to the variance explained by the model (R^2^). Might not add up to 100% due to rounding errors from parameter estimates

^b^for dichotomous variables, the information shown is for the underlined category (reference category not underlined)

^c^all 95% confidence intervals (95%CI) – for beta-weights, correlations, r-squared, and Pratt Index – are bootstrap confidence intervals based on 1000 samples

^d^bold numbers indicate covariates accounting for > 10% of the explained variance

^e^full time: > 35 h/week; part time/hourly: < 35 h/week
