# Supplementary material for: Meal analysis for understanding eating behavior: meal- and participant-specific predictors for the variance in energy and macronutrient intake
Source: Nutr J. 2019 Mar 7;18:15. doi: 10.1186/s12937-019-0440-8 (PMC6407220; doi:10.1186/s12937-019-0440-8)
Supplement: Supplementary file 1 — Box 1. Additional statistical methods: ICC calculation. Box 2. Additional statistical methods: Selection and description of covariates. Box 3. Additional statistical methods: R2and Pratt Index. Box 4. Additional statistical methods: Main models. Box 5. Additional statistical methods: 95% bootstrap confidence intervals. Figure S1. Flow diagram of participants of the validation sub-study within the EPIC-Potsdam cohort. Table S1. Eating occasions with participant-identified labels used to record food intake in the 24hDR. Table S2. Number of 24hDR and meals, total and by participant. Table S3. Relative importance of predictors of macronutrient intake (g/meal), Pratt Index overview. Table S4. Relative importance of predictors of macronutrient intake (g/meal), Pratt Index overview; sensitivity analysis adjusting for energy misreporting. Table S5. Random intercept Multilevel Regression Analysis and Corresponding Pratt for energy intake (kcal/meal). Table S6. Random intercept Multilevel Regression Analysis and Corresponding Pratt for carbohydrate intake (g/meal). Table S7. Random intercept Multilevel Regression Analysis and Corresponding Pratt for protein intake (g/meal). Table S8. Random intercept Multilevel Regression Analysis and Corresponding Pratt for fat intake (g/meal). Table S9. Random intercept Multilevel Regression Analysis and Corresponding Pratt for energy intake (kcal/meal); sensitivity analysis adjusting for energy misreporting. Table S10. Random intercept Multilevel Regression Analysis and Corresponding Pratt for carbohydrate intake (g/meal); sensitivity analysis adjusting for energy misreporting. Table S11. Random intercept Multilevel Regression Analysis and Corresponding Pratt for protein intake (g/meal); sensitivity analysis adjusting for energy misreporting. Table S12. Random intercept Multilevel Regression Analysis and Corresponding Pratt for fat intake (g/meal); sensitivity analysis adjusting for energy misreporting (DOCX 414 kb) [file 12937_2019_440_MOESM1_ESM.docx]

Meal analysis for understanding eating behavior: Meal- and participant-specific predictors for the variance in energy and macronutrient intake

Carolina Schwedhelm^1,2^, Khalid Iqbal^1,2^, Lukas Schwingshackl^3^, George O. Agogo^4^, Heiner Boeing^1^, Sven Knüppel^1,5^

^1^ Department of Epidemiology, German Institute of Human Nutrition Potsdam-Rehbruecke (DIfE), Arthur-Scheunert-Allee 114-116, 14558 Nuthetal, Germany

^2^ NutriAct – Competence Cluster Nutrition Research Berlin-Potsdam

^3^ Institute for Evidence in Medicine, Faculty of Medicine and Medical Center - University of Freiburg, Freiburg, Germany

^4^ Department of Internal Medicine, Yale School of Medicine, New Haven, CT, USA

^5^ Department of Nutrition and Gerontology, German Institute of Human Nutrition Potsdam-Rehbruecke (DIfE), Arthur-Scheunert-Allee 114-116, 14558 Nuthetal, Germany

Table of Contents

[Box 1: Additional statistical methods: *ICC calculation* 2](#_Toc536101153)

[Box 2: Additional statistical methods: *Selection and description of covariates* 3](#_Toc536101154)

[Box 3: Additional statistical methods: *R^2^ and Pratt index* 4](#_Toc536101155)

[Box 4: Additional statistical methods: *Main models* 5](#_Toc536101156)

[Box 5: Additional statistical methods: *95% bootstrap confidence intervals* 6](#_Toc536101157)

[Figure 1: Flow diagram of participants of the validation sub-study within the EPIC Potsdam cohort 7](#_Toc536101158)

[Table 1: Eating occasions with participant-identified labels used to record food intake in the 24hDR 8](#_Toc536101159)

[Table 2: Number of 24hDR and meals (total and by participant) 9](#_Toc536101160)

[Table 3: Relative importance of predictors of macronutrient intake (g/meal), Pratt Index overview 10](#_Toc536101161)

[Table 4: Relative importance of predictors of macronutrient intake (g/meal), Pratt Index overview; sensitivity analysis adjusting for energy misreporting 11](#_Toc536101162)

[Table 5: Random intercept Multilevel Regression Analysis and Corresponding Pratt for energy intake (kcal/meal) 13](#_Toc536101163)

[Table 6: Random intercept Multilevel Regression Analysis and Corresponding Pratt for carbohydrate intake (g/meal) 18](#_Toc536101164)

[Table 7: Random intercept Multilevel Regression Analysis and Corresponding Pratt for protein intake (g/meal) 23](#_Toc536101165)

[Table 8: Random intercept Multilevel Regression Analysis and Corresponding Pratt for fat intake (g/meal) 27](#_Toc536101166)

[Table 9: Random intercept Multilevel Regression Analysis and Corresponding Pratt for energy intake (kcal/meal); sensitivity analysis adjusting for energy misreporting 32](#_Toc536101167)

[Table 10: Random intercept Multilevel Regression Analysis and Corresponding Pratt for carbohydrate intake (g/meal); sensitivity analysis adjusting for energy misreporting 37](#_Toc536101168)

[Table 11: Random intercept Multilevel Regression Analysis and Corresponding Pratt for protein intake (g/meal); sensitivity analysis adjusting for energy misreporting 42](#_Toc536101169)

[Table 12: Random intercept Multilevel Regression Analysis and Corresponding Pratt for fat intake (g/meal); sensitivity analysis adjusting for energy misreporting 47](#_Toc536101170)

ICCs were obtained by dividing the variance in the level of interest by the total variance^1,2^. For our three-level model, the following equations were used:

${ICC}_{ID}= \frac{\sigma_{ID}^{2}}{\sigma_{ID}^{2}+\sigma_{meal}^{2}+\sigma_{e}^{2}}$

Participant level

Meal-type level

Intake level

$${ICC}_{meal}= \frac{\sigma_{meal}^{2}}{\sigma_{ID}^{2}+\sigma_{meal}^{2}+\sigma_{e}^{2}}$$

$${ICC}_{e}= \frac{\sigma_{e}^{2}}{\sigma_{ID}^{2}+\sigma_{meal}^{2}+\sigma_{e}^{2}}$$

where $\sigma_{ID}^{2}$, $\sigma_{meal}^{2}$, and $\sigma_{e}^{2}$ are the variances at the third (participant), second (meal type), and first (intake) level, respectively.

References:

1 Hox JJ: *Multilevel analysis: Techniques and applications.* 2nd edn: Routledge; 2010.

2 Bell BA, Ene M, Smiley W, Schoeneberger JA: **A multilevel model primer using SAS PROC MIXED.** In *SAS Global Forum*2013

# Box 1: Additional statistical methods: *ICC calculation*

Based on literature and availability of the data, we included the following covariates: *sex*, *age*, *BMI*, self-reported *physical activity* (hours of physical activity per week in the last 12 months including: sports, gardening, physical work, housework, cycling), *education level*, *current occupation*, *smoking status*, *duration of prior interval* (hours passed since last intake), *place of meal* (home, restaurant, work, or other), whether it was a *special day* or not (religious holiday, celebration meal, travel, or holidays), *season* (winter: October-March; summer: April-September), and whether it was a *weekday* (considered as Monday-Thursday) or *weekend day* (considered as Friday-Sunday). Data were missing for 1 person for *education level*, 9 persons for *current occupation* and *smoking status*, and 4 persons for *physical activity*. Missing values were imputed using single imputation by regression^1^ according to BMI, age, and sex. Predicted values of multivariable linear regression were imputed for continuous variables. For categorical variables, multivariable logistic regression models were fit for every classification group and missing values were replaced by the category with the highest probability.

References:

1 Enders CK: *Applied missing data analysis.* Guilford Press; 2010.

# Box 2: Additional statistical methods: *Selection and description of covariates*

We used the methods described by Liu et al.^1^ for estimating R^2^ for each level of regression model with random intercepts and calculating the Pratt Index (PI). The R^2^ is a measure of the model fit, defined as the total standardized variance in a population explained by a regression model with normally distributed residuals. The PI represents the proportion of R^2^ explained by each explanatory variable, ordering predictors in terms of their importance in a multiple regression analysis.

In a SEM framework, the covariance matrix of the observed individual variables can be partitioned into different levels of the model, resulting in an R^2^ with additive property for random-intercept regression models. The PI is a measure based on the standardized regression coefficient, the simple correlation between the response variable and explanatory variable, and on R^2^ and is calculated by levels using the following equation:

$${PI}_{j} = \frac{\hat{b}_{j}* r_{j}}{R^{2}}$$

where *j* denotes the number of observations/intakes in level 1 (intake level / within model) or the number of participants in level 2 (participant level / between model); $\hat{b}_{j}$ is the *j*_th_ standardized regression coefficient (“beta”) and $r_{j}$ is the zero-order correlation between the response variable and the *j*_th_ explanatory variable, estimated from the maximum-likelihood covariance matrix^1^. PI generally ranges from zero to one. Small negative numbers (between -½**m* and zero, where *m* is the number of predictors) can be set to zero. Larger negative numbers indicate collinearity or suppression, where the negative may contribute to the prediction of the outcome variable through another predictor^2^.

References:

1 Liu Y, Zumbo BD, Wu AD: **Relative importance of predictors in multilevel modeling.** *J Mod Appl Stat Methods* 2014, **13:**2.

2 Sajobi TT, Lix LM, Clara I, Walker J, Graff LA, Rawsthorne P, Miller N, Rogala L, Carr R, Bernstein CN: **Measures of relative importance for health-related quality of life.** *Quality of Life Research* 2012, **21:**1-11

# Box 3: Additional statistical methods: *R^2^ and Pratt index*

The meal type-stratified two-level random intercept model can be described as:

Level 1 (intake level / within model):

$$Y_{ij}= \beta_{0j}+ \beta_{1}{weekend}_{ij}+ \beta_{2}{season}_{ij}+ \beta_{3}{specialday}_{ij}+ \beta_{4}{priorinterval}_{ij}+ \beta_{5}{placeofmeal}_{ij}+ e_{ij}$$

Level 2 (participant level / between model):

$$\beta_{0j}= \gamma_{00}+ \gamma_{01}{bmi}_{j}+ \gamma_{02}{age}_{j}+ \gamma_{03}{sex}_{j}+ \gamma_{04}p\_{activity}_{j}+ \gamma_{05}{education}_{j}+ \gamma_{06}{occupation}_{j}+ \gamma_{07}{smoking}_{j}+ u_{0j}$$

where *i* denotes the number of observations/intakes; *j* denotes the number of participants; *β_0j_* is the random intercept for the participants; the other *β*s are the fixed slopes of the level 1 predictors; *ϒ_00_* is the model grand mean; the other *ϒ*s are the slopes for the level 2 predictors; *e_ij_* is the level 1 residual; and *u_0j_* is the level 2 residual for the random intercept. Reference group for *place of meal* was home, for *education* was no vocational training/current training, for *occupation* was no job/retired, and for *smoking* was never smokers.

# Box 4: Additional statistical methods: *Main models*

Bootstrap confidence intervals were calculated for standardized regression coefficients, correlations, R^2^, and Pratt Index according to the following procedure^1^:

1. Create 1000 bootstrap samples by randomly selecting participants with replacement from the original sample with sample size that is the same as the original sample. This sampling scheme ensures that the total number of participants remains constant but total number of eating occasions may vary.
2. For each bootstrap sample calculate statistic of interest $\hat{\theta}^{*}$ resulting in the nonparametric empirical bootstrap distribution for$\theta$.
3. 95% bootstrap confidence intervals are derived by taking the 2.5% and 97.5% percentile from the distribution of$\hat{\theta}^{*}$.

Statistical parameter $\theta$ of interest are beta-weights, correlations, R^2^, and Pratt Indices.

We generated the bootstrap samples by selecting participants (level 2) with replacements rather than selecting intake occasions (level 1), as this method preserves the structure of the data and implementing a selection based on level 1 units would overestimate the level 2 variation. Bootstrap samples were automated using R package MPlusAutomation^2^.

References:

1 Efron B, Tibshirani RJ. **An introduction to the bootstrap**. *CRC Press* 1994.

2 Hallquist M, Wiley J: **MplusAutomation: Automating Mplus model estimation and interpr** **etation.** *R package version 06 Available online at: http://cran r-project org/web/packages/MplusAutomation/index html* 2011.

# Box 5: Additional statistical methods: *95% bootstrap confidence intervals*

Potentially eligible sample

23,881 active EPIC-Potsdam follow-up participants

1447 invitations sent out

(age and sex stratified random draw)

815 participated in the sub-study

(56% participation rate)

896 individuals

682 participants with data for physical activity

(for the sensitivity analysis)

896 individuals

Final study sample (n=814)

1 participant excluded:

n = 1 with demetia

# Figure 1: Flow diagram of participants of the validation sub-study within the EPIC Potsdam cohort

# Table 1: Eating occasions with participant-identified labels used to record food intake in the 24hDR

| Eating occasion No. | Eating occasion participant-identified label |  | Mean meal time (SD), hhmm |
| --- | --- | --- | --- |
| 1 | Before breakfast |  | 6:52 (1:10) |
| 2 | Breakfast |  | 8:02 (1:08) |
| 3 | During morning |  | 9:59 (0:50) |
| 4 | Before lunch |  | 11:01 (0:39) |
| 5 | Lunch |  | 12:37 (0:46) |
| 6 | After lunch |  | 13:50 (0:41) |
| 7 | During afternoon (afternoon snack) |  | 15:31 (0:43) |
| 8 | Before dinner |  | 16:58 (0:38) |
| 9 | Dinner |  | 18:45 (0:54) |
| 10 | After dinner |  | 20:18 (1:16) |
| 11 | During evening |  | 21:41 (0:53) |

# Table 2: Number of 24hDR and meals (total and by participant)

|  | Observations (n) |
| --- | --- |
| **Total number of participants** | 814 |
| **Total number of 24hDR** | 2430 |
| Participants with number of 24hDR: |  |
| 3 | 805 |
| 2 | 6 |
| 1 | 3 |
| 0 | 0 |
| **Total number of main meals (intakes) consumed** | 8861 |
| **Total number of breakfast meals** | 2407 |
| Participants with number of breakfast meals: |  |
| 3 | 786 |
| 2 | 21 |
| 1 | 7 |
| 0 | 0 |
| **Total number of lunch meals** | 2188 |
| Participants with number of lunch meals: |  |
| 3 | 610 |
| 2 | 162 |
| 1 | 34 |
| 0 | 8 |
| **Total number of afternoon snacks** | 1953 |
| Participants with number of afternoon snacks: |  |
| 3 | 461 |
| 2 | 244 |
| 1 | 82 |
| 0 | 27 |
| **Total number of dinner meals** | 2313 |
| Participants with number of dinner meals: |  |
| 3 | 703 |
| 2 | 93 |
| 1 | 18 |
| 0 | 0 |

# Table 3: Relative importance of predictors of macronutrient intake (g/meal), Pratt Index overview^1^

| Covariates^2^ | Carbohydrates | | | | Protein | | | | Fat | | | |
| --- | --- | --- | --- | --- | --- | --- | --- | --- | --- | --- | --- | --- |
|  | Breakfast  PI (95%CI) | Lunch  PI (95%CI) | Afternoon snack  PI (95%CI) | Dinner  PI (95%CI) | Breakfast  PI (95%CI) | Lunch  PI (95%CI) | Afternoon snack  PI (95%CI) | Dinner  PI (95%CI) | Breakfast  PI (95%CI) | Lunch  PI (95%CI) | Afternoon snack  PI (95%CI) | Dinner  PI (95%CI) |
|  | **Intake-level covariates** | | | | | | | | | | | |
| Week/weekend day (y/n) | **13%**↑^3^(0;39) | **12%**↑(0;54) | **11%**↑(2;26) | 0% (0;19) | **35%**↑(15;52) | **17%**↑(4;35) | 6% (0;17) | 1% (0;13) | **32%**↑(12;51) | **18%**↑(1;43) | 9% (2;21) | 6% (0;30) |
| Season (winter/ summer) | **10%**↓(0;33) | **22%**↓(0;62) | 2% (0;9) | 5% (0;29) | 1% (0;7) | 0% (0;5) | 1% (0;8) | 4% (0;19) | 1% (0;8) | 2% (0;18) | 0% (0;4) | **10%**↑(0;34) |
| Special day (y/n) | 0% (-1;15) | 9% (0;49) | 5% (0;16) | **29%**↑(1;60) | 4% (-1;17) | 1% (0;9) | **10%**↑(1;23) | **15%**↑(0;40) | 1% (-1;13) | 9% (0;30) | 9% (1;21) | 9%(0;37) |
| Prior interval (hours) | 1% (0;34) | 0% (0;36) | **30%**↑(15;47) | **50%**↑(5;76) | 4% (0;18) | 0% (0;6) | **34%**↑(17;52) | **13%**↑(0;33) | 0% (0;9) | 4% (0;19) | **25%**↑(11;39) | 8% (0;38) |
| Place of meal (ref: home) |  |  |  |  |  |  |  |  |  |  |  |  |
| work | **65%**↓(3;86) | **34%**↓(0;74) | **40%**↓(18;59) | **10%**↓(0;49) | **17%**↓(0;44) | **59%**↓(28;79) | **41%**↓(19;60) | **16%**↓(0;46) | **28%**↓(3;59) | **38%**↓(3;69) | **45%**↓(22;63) | **39%**↓(0;75) |
| restaurant | **13%**↑(0;49) | **12%**↓(0;54) | 1% (0;7) | 4% (-1;42) | **40%**↑(19;58) | 8% (0;28) | 2% (0;9) | **51%**↑(15;71) | **37%**↑(15;59) | 8% (-1;34) | 4% (0;12) | **27%**↑(4;58) |
| other | 0% (0;16) | 4% (-1;36) | **11%**↑(2;24) | 0% (0;28) | 1% (0;11) | **15%**↓(2;38) | 6% (0;18) | 0% (0;23) | 1% (0;11) | **22%**↓(1;51) | **10%**↑(2;22) | 4% (0;36) |
|  |  |  |  |  |  |  |  |  |  |  |  |  |
| R-squared  (95%CI) | 0.02 (0.01;0.07) | 0.01 (0.00;0.02) | 0.07 (0.05;0.10) | 0.01 (0.01;0.03) | 0.05 (0.03;0.08) | 0.04 (0.03;0.07) | 0.07 (0.05;0.11) | 0.02 (0.01;0.05) | 0.05 (0.03;0.08) | 0.02 (0.01;0.05) | 0.08 (0.06;0.12) | 0.01 (0.01;0.04) |
|  | **Participant-level covariates** | | | | | | | | | | | |
|  |  |  |  |  |  |  |  |  |  |  |  |  |
| BMI (kg/m^2^) | 8% (0;23) | 4% (0;19) | 1% (0;25) | 1% (0;11) | 0% (0;8) | 0% (0;11) | 2% (0;29) | **19%**↑(6;33) | 4% (0;17) | 0% (0;11) | 0% (0;20) | 5% (0;15) |
| Age (years) | **14%**↑(-3;34) | **12%**↑ (-3;35) | 0% (-11;23) | 0% (-3;7) | 9% (-6;32) | 7% (-8;33) | 6% (-11;47) | 2% (-1;12) | 7% (-2;24) | **36%**↑(0;66) | 0% (-4;20) | 4% (-1;15) |
| Sex (M/W) | **41%**↓(20;57) | **65%**↓(32;79) | **68%**↓(11;85) | **95%**↓(67;98) | **71%**↓(35;85) | **74%**↓(33;86) | **32%**↓(-1;59) | **68%**↓(44;79) | **86%**↓(54;94) | **54%**↓(18;71) | **63%**↓(9;77) | **80%**↓(53;90) |
| Education level (ref. no training/ current training) |  |  |  |  |  |  |  |  |  |  |  |  |
| technical college | 0% (-1;5) | 2% (-1;15) | 0% (-2;18) | 2% (-2;14) | 3% (-2;17) | 3% (0;17) | 0% (-2;27) | 1% (-3;12) | 1% (0;10) | 0% (-1;9) | 0% (-1;18) | 7% (0;23) |
| university | 2% (-1;10) | 0% (-2;8) | 0% (-2;22) | 2% (-3;15) | 3% (-2;17) | 2% (-2;17) | 0% (-2;26) | 9% (0;24) | 3% (-2;15) | 1% (-1;14) | 3% (-2;28) | 0% (-3;9) |
| Occupation (ref. no job/ retired)^4^ |  |  |  |  |  |  |  |  |  |  |  |  |
| full time | 3% (-3;18) | 0% (-5;22) | 3% (-3;36) | 0% (-2;11) | 2% (-2;20) | 9% (-4;36) | **53%**↑(-4;78) | 2% (-3;14) | 0% (-4;10) | 1% (-10;26) | 3% (-3;36) | 0% (-2;7) |
| part time/hourly | 8% (0;25) | 0% (-2;10) | 0% (-1;13) | 0% (-1;7) | 6% (-1;21) | 0% (-1;15) | 0% (-2;34) | 0% (-1;5) | 0% (-2;4) | 0% (-3;15) | 1% (-1;16) | 3% (-1;12) |
| Physical activity (h/week) | 1% (0;8) | 6% (0;20) | 2% (0;19) | 0% (-1;5) | 1% (0;10) | 2% (0;11) | 3% (-2;30) | 0% (-2;4) | 2% (0;13) | 7% (0;26) | 4% (-1;20) | 0% (-1;5) |
| Smoking status (ref. never smoker) |  |  |  |  |  |  |  |  |  |  |  |  |
| current smoker | **21%**↑ (3;42) | **13%**↑ (-1;37) | **33%**↑(-1;73) | 0% (-12;7) | 0% (-10;12) | 0% (-10;10) | 1% (-8;39) | 0% (-7;14) | 0% (-6;8) | 0% (-5;14) | 0% (-12;20) | 3% (-5;17) |
| former smoker | 4% (-5;20) | 0% (-5;13) | 0% (-14;22) | 8% (-3;26) | 9% (-2;32) | 6% (-3;27) | 7% (-7;47) | 0% (-7;13) | 1% (-3;14) | 2% (-4;21) | **29%**↓(-2;67) | 0% (-8;10) |
|  |  |  |  |  |  |  |  |  |  |  |  |  |
| R-squared  (95%CI) | 0.17 (0.13;0.26) | 0.25 (0.16;0.55) | 0.07 (0.05;0.19) | 0.20 (0.15;0.35) | 0.10 (0.07;0.17) | 0.21 (0.13;0.61) | 0.03 (0.02;0.14) | 0.25 (0.19;0.39) | 0.12 (0.09;0.20) | 0.22 (0.12;0.72) | 0.06 (0.05;0.18) | 0.22 (0.17;0.33) |

95%CI, 95% bootstrap confidence intervals based on 1000 bootstrap samples; PI, Pratt Index

^1^ Pratt Index and 95% confidence intervals (bootstrap), in % contribution to the variance explained by the model (R^2^). Might not add up to 100% due to rounding errors from parameter estimates

^2^ for dichotomous variables, the information shown is for the underlined category (reference category not underlined)

^3^ bold numbers indicate covariates accounting for > 10% of the explained variance and arrows indicate the direction of the association

^4^ full time: > 35h/week; part time/hourly: < 35 h/week.

# Table 4: Relative importance of predictors of macronutrient intake (g/meal), Pratt Index overview; sensitivity analysis adjusting for energy misreporting^1,2^

| Covariates^3^ | Carbohydrates | | | | Protein | | | | Fat | | | |
| --- | --- | --- | --- | --- | --- | --- | --- | --- | --- | --- | --- | --- |
|  | Breakfast  PI (95%CI) | Lunch  PI (95%CI) | Afternoon snack  PI (95%CI) | Dinner  PI (95%CI) | Breakfast  PI (95%CI) | Lunch  PI (95%CI) | Afternoon snack  PI (95%CI) | Dinner  PI (95%CI) | Breakfast  PI (95%CI) | Lunch  PI (95%CI) | Afternoon snack  PI (95%CI) | Dinner  PI (95%CI) |
|  | **Intake-level covariates** | | | | | | | | | | | |
| Week/weekend day (y/n) | **16%** ↑^4^(2;46) | 2% (0;34) | **10%**↑(1;25) | 1% (0;24) | **35%**↑(14;52) | **15%**↑(3;34) | 5% (0;15) | 1% (0;19) | **34%**↑(13;54) | **27%**↑(3;54) | 8% (1;21) | **11%**↓(0;36) |
| Season (winter/ summer) | **12%**↓(1;43) | **17%**↓(0;55) | 4% (0;13) | 7% (0;32) | 1% (0;7) | 0% (0;7) | 1% (0;6) | **10%**↑(0;34) | 2% (0;8) | 2% (0;18) | 0% (0;4) | **13%**↑(0;38) |
| Special day (y/n) | 0% (-1;14) | 9% (0;41) | 3% (-1;13) | **21%**↑(0;52) | 1% (-2;15) | 1% (0;10) | 7% (0;19) | **16%**↑(0;44) | 2% (-2;14) | 9% (0;32) | 5% (-1;16) | 7% (0;28) |
| Prior interval (hours) | 0% (0;15) | 0% (0;33) | **33%**↑(14;50) | **50%**↑(3;77) | 3% (0;19) | 4% (0;17) | **38%** ↑ (19;56) | **13%**↑(0;38) | 2% (0;13) | **13%**↑(0;39) | **30%**↑(14;47) | **10%**↑(0;38) |
| Place of meal (ref: home) |  |  |  |  |  |  |  |  |  |  |  |  |
| work | **61%**↓(5;84) | **43%**↓(0;74) | **40%**↓(15;62) | **13%**↓(0;52) | **17%**↓(0;47) | **69%**↓(32;85) | **40%**↓(19;59) | **18%**↓(0;51) | **27%**↓(2;56) | **36%**↓(1;70) | **42%**↓(19;62) | **32%**↓(0-;68) |
| restaurant | 7% (0;33) | **20%**↑(0;56) | 0% (0;6) | 0% (-1;41) | **41%**↑(18;62) | 6% (-1;26) | 3% (0;10) | **39%**↑(5;63) | **33%**↑(12;55) | 7% (-1;35) | 7% (1;19) | **19%**↑(2;47) |
| other | 4% (0;19) | 9% (-1;45) | **11%**↑(2;26) | 5% (0;41) | 1% (0;12) | 4% (0;23) | 7% (0;20) | 2% (0;32) | 0% (0;14) | 6% (0;31) | 8% (1;20) | 9% (0;38) |
|  |  |  |  |  |  |  |  |  |  |  |  |  |
| R-squared  (95%CI) | 0.04 (0.02;0.09) | 0.01 (0.00;0.03) | 0.07 (0.04;0.10) | 0.01 (0.01;0.03) | 0.05 (0.04;0.09) | 0.04 (0.02;0.07) | 0.08 (0.06;0.12) | 0.02 (0.01;0.05) | 0.06 (0.04;0.09) | 0.02 (0.01;0.04) | 0.09 (0.06;0.13) | 0.02 (0.01;0.05) |
|  | **Participant-level covariates** | | | | | | | | | | | |
|  |  |  |  |  |  |  |  |  |  |  |  |  |
| BMI (kg/m^2^) | 1% (0;9) | 0% (-1;8) | 0% (-2;12) | 0% (-1;6) | 3% (0;14) | 1% (-1;11) | 0% (-3;17) | **15%**↑(4;28) | 0% (-1;7) | 0% (-1;7) | 0% (-2;11) | 7% (0;15) |
| Age (years) | 9% (-4;24) | 5% (-3;22) | 0% (-8;10) | 0% (-5;9) | 0% (-9;14) | 0% (-11;16) | 6% (-4;28) | 2% (-2;12) | 1% (-4;12) | **11%**↑(-1;36) | 2% (-4;14) | 0% (-5;4) |
| Sex (M/W) | **28%**↓(12;43) | **38%**↓(14;53) | **26%**↓(5;43) | **46%**↓(24;62) | **53%**↓(20;70) | **30%**↓(8;47) | 9% (0;33) | **41%**↓(21;54) | **59%**↓(33;73) | **21%**↓(3;39) | **26%**↓(4;45) | **37%**↓(19;52) |
| Education level (ref. no training/ current training) |  |  |  |  |  |  |  |  |  |  |  |  |
| technical college | 0% (-2;5) | 1% (-1;12) | 1% (-2;12) | 0% (-2;6) | 1% (-2;11) | 2% (0;11) | 2% (-2;18) | 0% (-3;5) | 0% (-1;6) | 2% (0;10) | 1% (-1;10) | 1% (-1;9) |
| university | 2% (0;10) | 0% (-2;8) | 1% (-2;12) | 4% (-1;13) | 4% (-1;15) | 0% (-1;8) | 1% (-1;15) | 1% (1;23) | 2% (-1;12) | 1% (-1;10) | 1% (-1;12) | 1% (-1;8) |
| Occupation (ref. no job/ retired)^5^ |  |  |  |  |  |  |  |  |  |  |  |  |
| full time | 0% (-3;9) | 0% (-5;13) | 0% (-2;16) | 0% (-2;8) | 1% (-2;14) | 8% (-2;30) | **22%**↑(-1;49) | 0% (-2;9) | 0% (-3;8) | 7% (-1;30) | 1% (-1;17) | 1% (-1;7) |
| part time/hourly | 9% (0;27) | 0% (-2;7) | 0% (-1;7) | 0% (-1;6) | 6% (-1;22) | 0% (-1;9) | 7% (0;24) | 0% (-1;3) | 0% (-2;4) | 0% (-1;7) | 3% (0;12) | 2% (0;7) |
| Physical activity (h/week) | 2% (0;8) | 5% (0;15) | 0% (0;7) | 0% (-1;4) | 1% (0;9) | 3% (0;11) | 0% (0;24) | 0% (-1;3) | 2% (0;10) | 4% (0;16) | 1% (-1;10) | 0% (0;4) |
| Smoking status (ref. never smoker) |  |  |  |  |  |  |  |  |  |  |  |  |
| current smoker | **20%**↑(4;37) | **11%**↑(0;31) | **11%**↑(0;35) | 0% (-6;9) | 0% (-9;11) | 0% (-4;8) | 1% (-1;10) | 2% (-5;15) | 0% (-5;7) | 0% (-2;8) | 0% (-7;11) | 2% (-3;11) |
| former smoker | 2% (-6;16) | 0% (-6;7) | 0% (-1;13) | 3% (-3;14) | 1% (-1;27) | 1% (-2;14) | 3% (-5;20) | 0% (-1;11) | 1% (-2;12) | 0% (-2;9) | **12%**↓(-2;34) | 0% (-5;8) |
| Energy misreporting |  |  |  |  |  |  |  |  |  |  |  |  |
| EI/TEE < 0.81 | **13%**↓(1;30) | **32%**↓(10;53) | **56%**↓(12;71) | **31%**↓(11;46) | **15%**↓(2;32) | **40%**↓(13;60) | **34%**↓(3;58) | **17%**↓(5;31) | **14%**↓(1;29) | **42%**↓(15;61) | **29%**↓(5;47) | **33%**↓(16;48) |
| EI/TEE > 1.19 | **14%**↑(5;24) | **11%**↑(1;23) | 9% (-1;22) | **20%**↑(8;32) | **15%**↑(2;29) | **16%**↑(4;29) | **17%**↑(0;37) | **15%**↑(1;25) | **24%**↑(3;54) | **13%**↑(3;26) | **30%**↑(10;46) | **19%**↑(10;28) |
|  |  |  |  |  |  |  |  |  |  |  |  |  |
| R-squared  (95%CI) | 0.22 (0.18;0.33) | 0.38 (0.28;0.72) | 0.20 (0.14;0.43) | 0.31 (0.24;0.50) | 0.13 (0.10;0.22) | 0.36 (0.24;0.83) | 0.12 (0.09;0.29) | 0.35 (0.28;0.51) | 0.16 (0.12;0.24) | 0.36 (0.22;0.86) | 0.17 (0.12;0.35) | 0.38 (0.32;0.53) |

95%CI, 95% bootstrap confidence intervals based on 1000 bootstrap samples; PI, Pratt Index

^1^ n=682 participants with activity sensor data

^2^ Pratt Index and 95% confidence intervals (bootstrap), in % contribution to the variance explained by the model (R^2^). Might not add up to 100% due to rounding errors from parameter estimates

^3^ for dichotomous variables, the information shown is for the underlined category (reference category not underlined)

^4^ bold numbers indicate covariates accounting for > 10% of the explained variance and arrows indicate the direction of the association

^5^ full time: > 35h/week; part time/hourly: < 35 h/week.

# Table 5: Random intercept Multilevel Regression Analysis and Corresponding Pratt for **energy intake** (kcal/meal)

| **Predictor**^1^ | **Beta-weight (95%CI)**^2^ | **t-test** | **p-value** | **Correlation**  **(95%CI)** | **Pratt Index**^3^ **(95%CI)** |
| --- | --- | --- | --- | --- | --- |
| **BREAKFAST** | | | | | |
| Within level (intake level) | | | | | |
| Week/weekend day (y/n) | 0.097  (0.042;0.143) | 3.899 | 0.000 | 0.110  (0.057;0.157) | 0.243  (0.053;0.463) |
| Season (winter/ summer) | -0.036  (-0.077;0.006) | -1.652 | 0.098 | -0.037  (-0.078;0.004) | 0.030  (0.000;0.156) |
| Special day (y/n) | 0.004  (-0.058;0.058) | 0.118 | 0.906 | 0.033  (-0.029;0.087) | 0.003  (-0.010;0.104) |
| Prior interval (hours) | -0.024  (-0.079;0.035) | -0.847 | 0.397 | -0.010  (-0.066;0.049) | 0.005  (-0.002;0.123) |
| Place of meal (ref. home) |  |  |  |  |  |
| work | -0.137  (-0.241;-0.035) | -2.607 | 0.009 | -0.143  (-0.249;-0.037) | 0.445  (0.038;0.725) |
| restaurant | 0.106  (0.063;0.153) | 5.017 | 0.000 | 0.112  (0.070;0.154) | 0.270  (0.079;0.535) |
| other | 0.007  (-0.062;0.067) | 0.224 | 0.823 | 0.009  (-0.059;0.068) | 0.001  (0.000;0.128) |
| **R-squared (95%CI)** | **0.044 (0.025;0.088)** | |  |  | **SUM = 1.0** |
| Between level (participant level) | | | | | |
| BMI (kg/m^2^) | -0.084  (-0.179;0.003) | -1.869 | 0.062 | -0.065  (-0.162;0.019) | 0.031  (-0.001;0.139) |
| Age (years) | 0.145  (0.019;0.257) | 2.337 | 0.019 | 0.212  (0.125;0.294) | 0.172  (0.017;0.334) |
| Sex (M/W) | -0.342  (-0.430;-0.254) | -7.588 | 0.000 | -0.335  (-0.406;-0.261) | 0.640  (0.385;0.804) |
| Education level (ref. no training/ current training) |  |  |  |  |  |
| technical college | -0.010  (-0.109;0.085) | -0.218 | 0.828 | -0.065  (-0.154;0.018) | 0.004  (-0.012;0.080) |
| university | -0.014  (-0.111;0.084) | -0.284 | 0.776 | 0.063  (-0.019;0.143) | -0.005  (-0.020;0.064) |
| Occupation (ref. no job/ retired)^4^ |  |  |  |  |  |
| full time | 0.021  (-0.113;0.155) | 0.296 | 0.767 | -0.064  (-0.161;0.034) | -0.008  (-0.03;0.083) |
| part time/hourly | -0.059  (-0.156;0.031) | -1.175 | 0.240 | -0.140  (-0.243;-0.037) | 0.046  (-0.008;0.177) |
| Physical activity (h/week) | 0.043  (-0.037;0.117) | 1.141 | 0.254 | 0.040  (-0.032;0.116) | 0.010  (-0.001;0.067) |
| Smoking status (ref. never smoker) |  |  |  |  |  |
| current smoker | 0.251  (0.079;0.420) | 2.796 | 0.005 | 0.035  (-0.044;0.118) | 0.049  (-0.038;0.192) |
| former smoker | 0.177  (0.012;0.348) | 1.986 | 0.047 | 0.059  (-0.029;0.140) | 0.058  (-0.010;0.198) |
| **R-squared (95%CI)** | **0.179 (0.133;0.263)** | |  |  | **SUM = 1.0** |
| **LUNCH** | | | | | |
| Within level (intake level) | | | | | |
| Week/weekend day (y/n) | 0.043  (-0.005;0.091) | 1.858 | 0.063 | 0.055  (0.007;0.102) | 0.103  (-0.001;0.331) |
| Season (winter/ summer) | 0.008  (-0.038;0.054) | 0.355 | 0.723 | -0.005  (-0.049;0.042) | -0.002  (-0.003;0.112) |
| Special day (y/n) | 0.044  (0.000;0.093) | 1.851 | 0.064 | 0.050  (0.002;0.097) | 0.096  (0.000;0.337) |
| Prior interval (hours) | 0.044  (0.003;0.084) | 1.990 | 0.047 | 0.053  (0.012;0.092) | 0.101  (0.002;0.344) |
| Place of meal (ref. home) |  |  |  |  |  |
| work | -0.115  (-0.188;-0.035) | -2.808 | 0.005 | -0.120  (-0.190;-0.044) | 0.600  (0.096;0.850) |
| restaurant | 0.018  (-0.034;0.068) | 0.697 | 0.486 | 0.049  (0.002;0.093) | 0.038  (-0.012;0.286) |
| other | -0.049  (-0.101;0.003) | -1.849 | 0.064 | -0.030  (-0.081;0.020) | 0.064  (-0.004;0.313) |
| **R-squared (95%CI)** | **0.023 (0.012;0.049)** | |  |  | **SUM = 1.0** |
| Between level (participant level) | | | | | |
| BMI (kg/m^2^) | -0.069  (-0.213;0.057) | -1.033 | 0.302 | -0.042  (-0.187;0.08) | 0.011  (-0.002;0.117) |
| Age (years) | 0.117  (-0.070;0.293) | 1.236 | 0.217 | 0.284  (0.131;0.426) | 0.120  (-0.040;0.329) |
| Sex (M/W) | -0.461  (-0.654;-0.313) | -6.799 | 0.000 | -0.395  (-0.557;-0.270) | 0.660  (0.338;0.822) |
| Education level (ref. no training/ current training) |  |  |  |  |  |
| technical college | -0.034  (-0.170;0.092) | -0.498 | 0.618 | -0.043  (-0.174;0.084) | 0.005  (-0.005;0.083) |
| university | -0.100  (-0.248;0.032) | -1.465 | 0.143 | -0.021  (-0.156;0.108) | 0.008  (-0.014;0.103) |
| Occupation (ref. no job/ retired)^4^ |  |  |  |  |  |
| full time | -0.092  (-0.304;0.102) | -0.878 | 0.380 | -0.193  (-0.375;-0.020) | 0.064  (-0.030;0.277) |
| part time/hourly | -0.003  (-0.170;0.161) | -0.034 | 0.973 | -0.078  (-0.227;0.065) | 0.001  (-0.014;0.115) |
| Physical activity (h/week) | 0.137  (0.004;0.276) | 2.166 | 0.030 | 0.149  (0.022;0.269) | 0.074  (0.001;0.190) |
| Smoking status (ref. never smoker) |  |  |  |  |  |
| current smoker | 0.228  (-0.004;0.480) | 1.910 | 0.056 | 0.048  (-0.078;0.158) | 0.040  (-0.037;0.201) |
| former smoker | 0.115  (-0.124;0.380) | 0.921 | 0.357 | 0.030  (-0.088;0.157) | 0.013  (-0.020;0.154) |
| **R-squared (95%CI)** | **0.276 (0.184;0.539)** | |  |  | **SUM = 1.0** |
| **AFTERNOON SNACK** | | | | | |
| Within level (intake level) | | | | | |
| Week/weekend day (y/n) | 0.098  (0.048;0.146) | 3.970 | 0.000 | 0.128  (0.081;0.172) | 0.118  (0.036;0.222) |
| Season (winter/ summer) | 0.027  (-0.016;0.069) | 1.219 | 0.223 | 0.023  (-0.022;0.065) | 0.006  (0.000;0.041) |
| Special day (y/n) | 0.069  (0.023;0.116) | 2.792 | 0.005 | 0.114  (0.064;0.160) | 0.074  (0.014;0.162) |
| Prior interval (hours) | 0.152  (0.105;0.196) | 6.425 | 0.000 | 0.189  (0.142;0.234) | 0.271  (0.145;0.397) |
| Place of meal (ref. home) |  |  |  |  |  |
| work | -0.191  (-0.253;-0.132) | -5.917 | 0.000 | -0.238  (-0.297;-0.177) | 0.429  (0.242;0.596) |
| restaurant | 0.018  (-0.032;0.065) | 0.700 | 0.484 | 0.042  (-0.008;0.089) | 0.007  (-0.002;0.052) |
| other | 0.079  (0.029;0.127) | 3.157 | 0.002 | 0.131  (0.083;0.177) | 0.098  (0.025;0.196) |
| **R-squared (95%CI)** | **0.106 (0.081;0.141)** | |  |  | **SUM = 1.0** |
| Between level (participant level) | | | | | |
| BMI (kg/m^2^) | -0.034  (-0.173;0.081) | -0.554 | 0.579 | -0.042  (-0.177;0.072) | 0.020  (-0.001;0.235) |
| Age (years) | -0.013  (-0.177;0.183) | -0.144 | 0.885 | 0.039  (-0.088;0.173) | -0.007  (-0.069;0.242) |
| Sex (M/W) | -0.264  (-0.388;-0.136) | -4.249 | 0.000 | -0.206  (-0.314;-0.099) | 0.755  (0.171;0.858) |
| Education level (ref. no training/ current training) |  |  |  |  |  |
| technical college | 0.026  (-0.095;0.153) | 0.395 | 0.693 | -0.014  (-0.127;0.098) | -0.005  (-0.017;0.156) |
| university | -0.018  (-0.153;0.125) | -0.269 | 0.788 | 0.023  (-0.100;0.138) | -0.006  (-0.018;0.181) |
| Occupation (ref. no job/ retired)^4^ |  |  |  |  |  |
| full time | 0.012  (-0.168;0.224) | 0.125 | 0.901 | 0.009  (-0.132;0.149) | 0.002  (-0.038;0.260) |
| part time/hourly | -0.008  (-0.129;0.102) | -0.143 | 0.887 | -0.045  (-0.168;0.059) | 0.005  (-0.018;0.199) |
| Physical activity (h/week) | 0.045  (-0.052;0.149) | 0.869 | 0.385 | 0.024  (-0.083;0.130) | 0.015  (-0.006;0.165) |
| Smoking status (ref. never smoker) |  |  |  |  |  |
| current smoker | 0.195  (-0.038;0.418) | 1.763 | 0.078 | 0.091  (-0.028;0.212) | 0.246  (-0.027;0.616) |
| former smoker | 0.044  (-0.175;0.253) | 0.396 | 0.692 | -0.049  (-0.168;0.069) | -0.030  (-0.118;0.219) |
| **R-squared (95%CI)** | **0.072 (0.048;0.188)** | |  |  | **SUM = 1.0** |
| **DINNER** | | | | | |
| Within level (intake level) | | | | | |
| Week/weekend day (y/n) | -0.022  (-0.066;0.024) | -0.909 | 0.364 | -0.015  (-0.058;0.032) | 0.011  (-0.001;0.118) |
| Season (winter/ summer) | 0.031  (-0.012;0.074) | 1.401 | 0.161 | 0.034  (-0.010;0.077) | 0.035  (0.000;0.157) |
| Special day (y/n) | 0.056  (0.007;0.102) | 2.385 | 0.017 | 0.085  (0.041;0.129) | 0.159  (0.008;0.384) |
| Prior interval (hours) | 0.066  (0.023;0.107) | 3.006 | 0.003 | 0.079  (0.036;0.120) | 0.174  (0.0260;0.360) |
| Place of meal (ref. home) |  |  |  |  |  |
| work | -0.070  (-0.144;-0.004) | -1.845 | 0.065 | -0.075  (-0.148;-0.012) | 0.175  (0.003;0.477) |
| restaurant | 0.106  (0.062;0.150) | 4.933 | 0.000 | 0.122  (0.079;0.163) | 0.431  (0.170;0.635) |
| other | 0.014  (-0.048;0.072) | 0.432 | 0.666 | 0.022  (-0.038;0.078) | 0.010  (-0.001;0.165) |
| **R-squared (95%CI)** | **0.030 (0.020;0.053)** | |  |  | **SUM = 1.0** |
| Between level (participant level) | | | | | |
| BMI (kg/m^2^) | 0.077  (-0.021;0.172) | 1.560 | 0.119 | 0.085  (-0.014;0.175) | 0.023  (0.000;0.092) |
| Age (years) | -0.077  (-0.217;0.070) | -1.101 | 0.271 | -0.032  (-0.132;0.086) | 0.009  (-0.011;0.075) |
| Sex (M/W) | -0.507  (-0.584;-0.429) | -11.835 | 0.000 | -0.502  (-0.572;-0.435) | 0.903  (0.719;0.948) |
| Education level (ref. no training/ current training) |  |  |  |  |  |
| technical college | -0.040  (-0.150;0.072) | -0.737 | 0.461 | -0.150  (-0.250;-0.047) | 0.021  (-0.017;0.115) |
| university | 0.032  (-0.072;0.144) | 0.586 | 0.558 | 0.153  (0.063;0.257) | 0.017  (-0.020;0.113) |
| Occupation (ref. no job/ retired)^4^ |  |  |  |  |  |
| full time | 0.017  (-0.120;0.150) | 0.241 | 0.809 | 0.084  (-0.022;0.183) | 0.005  (-0.021;0.075) |
| part time/hourly | 0.094  (-0.010;0.191) | 1.866 | 0.062 | 0.029  (-0.072;0.120) | 0.010  (-0.008;0.068) |
| Physical activity (h/week) | 0.068  (-0.019;0.155) | 1.577 | 0.115 | -0.033  (-0.126;0.053) | -0.008  (-0.016;0.028) |
| Smoking status (ref. never smoker) |  |  |  |  |  |
| current smoker | 0.050  (-0.128;0.243) | 0.555 | 0.579 | -0.167  (-0.266;-0.065) | -0.030  (-0.106;0.089) |
| former smoker | 0.076  (-0.099;0.268) | 0.844 | 0.399 | 0.180  (0.079;0.278) | 0.049  (-0.041;0.201) |
| **R-squared (95%CI)** | **0.282 (0.232;0.386)** | |  |  | **SUM = 1.0** |

^1^ for dichotomous variables, the information shown is for the underlined category (reference category not underlined)

^2^ all 95% confidence intervals (95%CI) – for beta-weights, correlations, r-squared, and Pratt Index – are bootstrap confidence intervals based on 1000 bootstrap samples

^3^ might not add up to 100% due to rounding errors from parameter estimates

^4^ full time: > 35h/week; part time/hourly: < 35 h/week.

# Table 6: Random intercept Multilevel Regression Analysis and Corresponding Pratt for **carbohydrate intake** (g/meal)

| **Predictor**^1^ | **Beta-weight (95%CI)**^2^ | **t-test** | **p-value** | **Correlation**  **(95%CI)** | **Pratt Index**^2^ **(95%CI)** |
| --- | --- | --- | --- | --- | --- |
| **BREAKFAST** | | | | | |
| Within level (intake level) | | | | | |
| Week/weekend day (y/n) | 0.047  (-0.001;0.098) | 1.983 | 0.047 | 0.058  (0.007;0.111) | 0.130  (0.001;0.388) |
| Season (winter/ summer) | -0.046  (-0.089;0.001) | -2.000 | 0.046 | -0.045  (-0.086;-0.001) | 0.099  (0.001;0.332) |
| Special day (y/n) | -0.006  (-0.06;0.043) | -0.208 | 0.835 | 0.008  (-0.042;0.058) | -0.002  (-0.005;0.147) |
| Prior interval (hours) | -0.021  (-0.078;0.034) | -0.741 | 0.459 | -0.011  (-0.071;0.047) | 0.011  (-0.002;0.336) |
| Place of meal (ref. home) |  |  |  |  |  |
| work | -0.116  (-0.234;-0.017) | -2.112 | 0.035 | -0.118  (-0.235;-0.018) | 0.652  (0.032;0.864) |
| restaurant | 0.051  (0.006;0.100) | 2.197 | 0.028 | 0.053  (0.011;0.098) | 0.129  (0.004;0.492) |
| other | 0.006  (-0.047;0.057) | 0.222 | 0.824 | 0.006  (-0.047;0.057) | 0.002  (0.000;0.155) |
| **R-squared (95%CI)** | **0.021 (0.008;0.069)** | |  |  | **SUM = 1.0** |
| Between level (participant level) | | | | | |
| BMI (kg/m^2^) | -0.122  (-0.209;-0.023) | -2.593 | 0.010 | -0.109  (-0.203;-0.014) | 0.077  (0.002;0.225) |
| Age (years) | 0.107  (-0.033;0.235) | 1.624 | 0.104 | 0.224  (0.132;0.307) | 0.139  (-0.034;0.339) |
| Sex (M/W) | -0.273  (-0.356;-0.189) | -6.312 | 0.000 | -0.256  (-0.325;-0.182) | 0.406  (0.197;0.571) |
| Education level (ref. no training/ current training) |  |  |  |  |  |
| technical college | 0.030  (-0.063;0.124) | 0.631 | 0.528 | -0.036  (-0.124;0.049) | -0.006  (-0.013;0.046) |
| university | 0.043  (-0.058;0.135) | 0.902 | 0.367 | 0.084  (-0.005;0.166) | 0.021  (-0.009;0.098) |
| Occupation (ref. no job/ retired)^4^ |  |  |  |  |  |
| full time | -0.047  (-0.194;0.096) | -0.627 | 0.531 | -0.106  (-0.207;-0.02) | 0.029  (-0.026;0.183) |
| part time/hourly | -0.094  (-0.201;0.012) | -1.749 | 0.080 | -0.144  (-0.251;-0.041) | 0.079  (-0.002;0.249) |
| Physical activity (h/week) | 0.039  (-0.044;0.124) | 0.893 | 0.372 | 0.054  (-0.026;0.131) | 0.012  (-0.002;0.078) |
| Smoking status (ref. never smoker) |  |  |  |  |  |
| current smoker | 0.349  (0.182;0.526) | 3.924 | 0.000 | 0.102  (0.018;0.189) | 0.207  (0.028;0.424) |
| former smoker | 0.248  (0.065;0.422) | 2.761 | 0.006 | 0.025  (-0.069;0.113) | 0.036  (-0.049;0.204) |
| **R-squared (95%CI)** | **0.172 (0.126;0.263)** | |  |  | **SUM = 1.0** |
| **LUNCH** | | | | | |
| Within level (intake level) | | | | | |
| Week/weekend day (y/n) | 0.021  (-0.025;0.064) | 0.912 | 0.362 | 0.028  (-0.018;0.070) | 0.118  (-0.002;0.541) |
| Season (winter/ summer) | -0.032  (-0.075;0.012) | -1.449 | 0.147 | -0.034  (-0.076;0.010) | 0.218  (0.000;0.612) |
| Special day (y/n) | 0.022  (-0.021;0.065) | 1.009 | 0.313 | 0.021  (-0.020;0.061) | 0.092  (-0.004;0.487) |
| Prior interval (hours) | 0.001  (-0.047;0.046) | 0.057 | 0.954 | 0.004  (-0.045;0.050) | 0.001  (0.000;0.364) |
| Place of meal (ref. home) |  |  |  |  |  |
| work | -0.040  (-0.110;0.032) | -1.108 | 0.268 | -0.042  (-0.109;0.027) | 0.336  (0.000;0.742) |
| restaurant | -0.029  (-0.091;0.024) | -0.983 | 0.326 | -0.020  (-0.079;0.026) | 0.116  (-0.004;0.539) |
| other | 0.010  (-0.035;0.051) | 0.456 | 0.648 | 0.018  (-0.023;0.055) | 0.036  (-0.007;0.364) |
| **R-squared (95%CI)** | **0.005 (0.002;0.022)** | |  |  | **SUM = 0.92** |
| Between level (participant level) | | | | | |
| BMI (kg/m^2^) | -0.109  (-0.259;0.036) | -1.500 | 0.134 | -0.096  (-0.250;0.038) | 0.041  (0.000;0.194) |
| Age (years) | 0.131  (-0.081;0.347) | 1.289 | 0.197 | 0.227  (0.034;0.409) | 0.118  (-0.030;0.348) |
| Sex (M/W) | -0.436  (-0.629;-0.288) | -5.266 | 0.000 | -0.374  (-0.524;-0.236) | 0.645  (0.315;0.788) |
| Education level (ref. no training/ current training) |  |  |  |  |  |
| technical college | -0.056  (-0.236;0.120) | -0.653 | 0.514 | -0.094  (-0.250;0.053) | 0.021  (-0.009;0.153) |
| university | -0.053  (-0.226;0.108) | -0.670 | 0.503 | 0.050  (-0.090;0.191) | -0.010  (-0.023;0.082) |
| Occupation (ref. no job/ retired)^4^ |  |  |  |  |  |
| full time | -0.005  (-0.259;0.246) | -0.037 | 0.971 | -0.116  (-0.327;0.098) | 0.002  (-0.052;0.215) |
| part time/hourly | 0.030  (-0.141;0.211) | 0.356 | 0.722 | -0.061  (-0.209;0.093) | -0.007  (-0.019;0.095) |
| Physical activity (h/week) | 0.124  (-0.004;0.270) | 1.740 | 0.082 | 0.116  (-0.011;0.238) | 0.057  (-0.001;0.199) |
| Smoking status (ref. never smoker) |  |  |  |  |  |
| current smoker | 0.313  (0.045;0.592) | 2.249 | 0.025 | 0.105  (-0.023;0.235) | 0.130  (-0.011;0.370) |
| former smoker | 0.150  (-0.117;0.442) | 1.087 | 0.277 | -0.009  (-0.145;0.124) | -0.005  (-0.055;0.132) |
| **R-squared (95%CI)** | **0.253 (0.161;0.551)** | |  |  | **SUM = 1.0** |
| **AFTERNOON SNACK** | | | | | |
| Within level (intake level) | | | | | |
| Week/weekend day (y/n) | 0.074  (0.027;0.122) | 3.060 | 0.002 | 0.095  (0.051;0.144) | 0.108  (0.020;0.257) |
| Season (winter/ summer) | 0.039  (-0.007;0.083) | 1.711 | 0.087 | 0.036  (-0.01;0.080) | 0.022  (0.000;0.094) |
| Special day (y/n) | 0.044  (-0.005;0.092) | 1.839 | 0.066 | 0.080  (0.035;0.124) | 0.054  (-0.002;0.160) |
| Prior interval (hours) | 0.127  (0.084;0.176) | 5.444 | 0.000 | 0.155  (0.11;0.203) | 0.303  (0.151;0.470) |
| Place of meal (ref. home) |  |  |  |  |  |
| work | -0.145  (-0.207;-0.080) | -4.556 | 0.000 | -0.181  (-0.241;-0.120) | 0.404  (0.175;0.590) |
| restaurant | 0.013  (-0.036;0.060) | 0.544 | 0.587 | 0.030  (-0.015;0.074) | 0.006  (-0.002;0.067) |
| other | 0.066  (0.020;0.110) | 2.884 | 0.004 | 0.106  (0.060;0.149) | 0.108  (0.020;0.244) |
| **R-squared (95%CI)** | **0.065 (0.047;0.096)** | |  |  | **SUM = 1.0** |
| Between level (participant level) | | | | | |
| BMI (kg/m^2^) | -0.019  (-0.163;0.108) | -0.285 | 0.775 | -0.029  (-0.171;0.100) | 0.008  (-0.001;0.248) |
| Age (years) | -0.040  (-0.213;0.156) | -0.430 | 0.667 | 0.053  (-0.069;0.192) | -0.032  (-0.108;0.234) |
| Sex (M/W) | -0.253  (-0.386;-0.121) | -3.969 | 0.000 | -0.181  (-0.309;-0.058) | 0.683  (0.106;0.849) |
| Education level (ref. no training/ current training) |  |  |  |  |  |
| technical college | 0.035  (-0.098;0.170) | 0.521 | 0.603 | 0.006  (-0.108;0.117) | 0.003  (-0.017;0.182) |
| university | -0.021  (-0.168;0.124) | -0.304 | 0.761 | 0.004  (-0.121;0.133) | -0.001  (-0.015;0.215) |
| Occupation (ref. no job/ retired)^4^ |  |  |  |  |  |
| full time | -0.052  (-0.24;0.157) | -0.512 | 0.609 | -0.041  (-0.175;0.107) | 0.032  (-0.034;0.359) |
| part time/hourly | 0.004  (-0.120;0.110) | 0.080 | 0.936 | -0.015  (-0.126;0.073) | -0.001  (-0.012;0.133) |
| Physical activity (h/week) | 0.038  (-0.076;0.135) | 0.709 | 0.478 | 0.031  (-0.08;0.141) | 0.018  (-0.003;0.187) |
| Smoking status (ref. never smoker) |  |  |  |  |  |
| current smoker | 0.212  (0.020;0.436) | 1.975 | 0.048 | 0.104  (-0.011;0.232) | 0.329  (-0.013;0.725) |
| former smoker | 0.055  (-0.140;0.255) | 0.532 | 0.595 | -0.056  (-0.185;0.067) | -0.046  (-0.138;0.215) |
| **R-squared (95%CI)** | **0.067 (0.047;0.191)** | |  |  | **SUM = 1.0** |
| **DINNER** | | | | | |
| Within level (intake level) | | | | | |
| Week/weekend day (y/n) | -0.005  (-0.051;0.041) | -0.207 | 0.836 | -0.004  (-0.048;0.042) | 0.002  (0.000;0.193) |
| Season (winter/ summer) | 0.022  (-0.025;0.062) | 1.003 | 0.316 | 0.024  (-0.021;0.064) | 0.053  (0.000;0.288) |
| Special day (y/n) | 0.052  (0.006;0.096) | 2.297 | 0.022 | 0.055  (0.011;0.099) | 0.286  (0.010;0.599) |
| Prior interval (hours) | 0.068  (0.021;0.116) | 2.733 | 0.006 | 0.073  (0.026;0.121) | 0.496  (0.054;0.756) |
| Place of meal (ref. home) |  |  |  |  |  |
| work | -0.030  (-0.095;0.020) | -1.036 | 0.300 | -0.033  (-0.096;0.016) | 0.099  (0.000;0.490) |
| restaurant | 0.014  (-0.054;0.078) | 0.380 | 0.704 | 0.030  (-0.040;0.093) | 0.042  (-0.008;0.419) |
| other | -0.012  (-0.071;0.044) | -0.428 | 0.668 | -0.001  (-0.057;0.053) | 0.001  (-0.004;0.275) |
| **R-squared (95%CI)** | **0.010 (0.006;0.028)** | |  |  | **SUM = 1.0** |
| Between level (participant level) | | | | | |
| BMI (kg/m^2^) | -0.050  (-0.166;0.065) | -0.850 | 0.396 | -0.041  (-0.155;0.072) | 0.010  (-0.001;0.109) |
| Age (years) | -0.038  (-0.193;0.135) | -0.458 | 0.647 | 0.021  (-0.084;0.128) | -0.004  (-0.032;0.070) |
| Sex (M/W) | -0.442  (-0.561;-0.335) | -7.832 | 0.000 | -0.436  (-0.543;-0.339) | 0.949  (0.667;0.978) |
| Education level (ref. no training/ current training) |  |  |  |  |  |
| technical college | -0.027  (-0.158;0.09) | -0.420 | 0.675 | -0.118  (-0.233;-0.015) | 0.016  (-0.02;0.142) |
| university | 0.022  (-0.097;0.144) | 0.360 | 0.719 | 0.135  (0.024;0.233) | 0.015  (-0.027;0.150) |
| Occupation (ref. no job/ retired)^4^ |  |  |  |  |  |
| full time | -0.011  (-0.181;0.168) | -0.119 | 0.906 | 0.041  (-0.082;0.161) | -0.002  (-0.022;0.106) |
| part time/hourly | 0.043  (-0.085;0.160) | 0.724 | 0.469 | -0.004  (-0.114;0.097) | -0.001  (-0.009;0.065) |
| Physical activity (h/week) | 0.018  (-0.083;0.115) | 0.366 | 0.714 | -0.055  (-0.151;0.039) | -0.005  (-0.014;0.052) |
| Smoking status (ref. never smoker) |  |  |  |  |  |
| current smoker | 0.119  (-0.088;0.333) | 1.111 | 0.266 | -0.100  (-0.207;0.013) | -0.059  (-0.115;0.066) |
| former smoker | 0.119  (-0.084;0.319) | 1.143 | 0.253 | 0.138  (0.02;0.235) | 0.081  (-0.029;0.256) |
| **R-squared (95%CI)** | **0.203 (0.15;0.345)** | |  |  | **SUM = 1.0** |

^1^ for dichotomous variables, the information shown is for the underlined category (reference category not underlined)

^2^ all 95% confidence intervals (95%CI) – for beta-weights, correlations, r-squared, and Pratt Index – are bootstrap confidence intervals based on 1000 bootstrap samples

^3^ might not add up to 100% due to rounding errors from parameter estimates

^4^ full time: > 35h/week; part time/hourly: < 35 h/week

# Table 7: Random intercept Multilevel Regression Analysis and Corresponding Pratt for **protein intake** (g/meal)

| **Predictor**^1^ | **Beta-weight (95%CI)**^2^ | **t-test** | **p-value** | **Correlation**  **(95%CI)** | **Pratt Index**^2^ **(95%CI)** |
| --- | --- | --- | --- | --- | --- |
| **BREAKFAST** | | | | | |
| Within level (intake level) | | | | | |
| Week/weekend day (y/n) | 0.126  (0.082;0.168) | 5.592 | 0.000 | 0.133  (0.089;0.175) | 0.349  (0.147;0.517) |
| Season (winter/ summer) | 0.024  (-0.019;0.064) | 1.135 | 0.256 | 0.020  (-0.022;0.061) | 0.010  (0.000;0.071) |
| Special day (y/n) | 0.026  (-0.034;0.079) | 0.888 | 0.374 | 0.064  (0.006;0.116) | 0.035  (-0.009;0.169) |
| Prior interval (hours) | -0.048  (-0.100;0.004) | -1.721 | 0.085 | -0.037  (-0.092;0.018) | 0.037  (-0.001;0.184) |
| Place of meal (ref. home) |  |  |  |  |  |
| work | -0.086  (-0.173;0.006) | -1.891 | 0.059 | -0.094  (-0.182;-0.001) | 0.168  (0.002;0.442) |
| restaurant | 0.133  (0.092;0.172) | 7.019 | 0.000 | 0.145  (0.105;0.182) | 0.402  (0.192;0.579) |
| other | 0.017  (-0.048;0.075) | 0.547 | 0.584 | 0.025  (-0.036;0.083) | 0.009  (-0.001;0.112) |
| **R-squared (95%CI)** | **0.048 (0.034;0.080)** | |  |  | **SUM = 1.0** |
| Between level (participant level) | | | | | |
| BMI (kg/m^2^) | -0.009  (-0.094;0.078) | -0.214 | 0.831 | 0.007  (-0.072;0.091) | -0.001  (-0.002;0.077) |
| Age (years) | 0.061  (-0.068;0.190) | 0.933 | 0.351 | 0.144  (0.058;0.232) | 0.086  (-0.057;0.320) |
| Sex (M/W) | -0.263  (-0.348;-0.170) | -5.655 | 0.000 | -0.277  (-0.351;-0.193) | 0.714  (0.347;0.850) |
| Education level (ref. no training/ current training) |  |  |  |  |  |
| technical college | -0.031  (-0.124;0.056) | -0.666 | 0.506 | -0.091  (-0.172;-0.009) | 0.028  (-0.019;0.165) |
| university | 0.034  (-0.063;0.126) | 0.695 | 0.487 | 0.093  (0.012;0.174) | 0.031  (-0.018;0.173) |
| Occupation (ref. no job/ retired)^4^ |  |  |  |  |  |
| full time | -0.040  (-0.190;0.104) | -0.549 | 0.583 | -0.054  (-0.151;0.040) | 0.021  (-0.019;0.201) |
| part time/hourly | -0.059  (-0.147;0.036) | -1.223 | 0.221 | -0.104  (-0.190;-0.019) | 0.060  (-0.012;0.208) |
| Physical activity (h/week) | 0.031  (-0.057;0.115) | 0.754 | 0.451 | 0.022  (-0.063;0.106) | 0.007  (-0.002;0.100) |
| Smoking status (ref. never smoker) |  |  |  |  |  |
| current smoker | 0.123  (-0.042;0.278) | 1.515 | 0.130 | -0.027  (-0.113;0.061) | -0.033  (-0.098;0.119) |
| former smoker | 0.110  (-0.048;0.272) | 1.365 | 0.172 | 0.083  (-0.003;0.168) | 0.090  (-0.016;0.316) |
| **R-squared (95%CI)** | **0.102 (0.072;0.173)** | |  |  | **SUM = 1.0** |
| **LUNCH** | | | | | |
| Within level (intake level) | | | | | |
| Week/weekend day (y/n) | 0.077  (0.033;0.120) | 3.326 | 0.001 | 0.090  (0.048;0.136) | 0.165  (0.039;0.351) |
| Season (winter/ summer) | 0.011  (-0.032;0.051) | 0.512 | 0.609 | -0.008  (-0.049;0.031) | -0.002  (-0.004;0.046) |
| Special day (y/n) | 0.016  (-0.035;0.060) | 0.642 | 0.521 | 0.021  (-0.028;0.068) | 0.008  (-0.001;0.094) |
| Prior interval (hours) | 0.003  (-0.040;0.047) | 0.163 | 0.870 | 0.014  (-0.031;0.057) | 0.001  (-0.001;0.064) |
| Place of meal (ref. home) |  |  |  |  |  |
| work | -0.155  (-0.221;-0.086) | -4.647 | 0.000 | -0.160  (-0.226;-0.096) | 0.590  (0.277;0.792) |
| restaurant | 0.044  (-0.003;0.097) | 1.729 | 0.084 | 0.077  (0.03;0.125) | 0.081  (-0.002;0.276) |
| other | -0.087  (-0.14;-0.031) | -3.028 | 0.002 | -0.071  (-0.126;-0.019) | 0.147  (0.016;0.375) |
| **R-squared (95%CI)** | **0.042 (0.025;0.071)** | |  |  | **SUM = 1.0** |
| Between level (participant level) | | | | | |
| BMI (kg/m^2^) | -0.031  (-0.19;0.111) | -0.417 | 0.677 | -0.002  (-0.163;0.145) | 0.000  (-0.002;0.105) |
| Age (years) | 0.073  (-0.144;0.302) | 0.627 | 0.531 | 0.208  (0.050;0.390) | 0.072  (-0.082;0.330) |
| Sex (M/W) | -0.415  (-0.656;-0.259) | -4.295 | 0.000 | -0.378  (-0.578;-0.237) | 0.740  (0.330;0.855) |
| Education level (ref. no training/ current training) |  |  |  |  |  |
| technical college | -0.087  (-0.260;0.062) | -1.145 | 0.252 | -0.070  (-0.226;0.067) | 0.029  (-0.003;0.169) |
| university | -0.134  (-0.306;0.013) | -1.662 | 0.096 | -0.032  (-0.174;0.105) | 0.020  (-0.021;0.172) |
| Occupation (ref. no job/ retired)^4^ |  |  |  |  |  |
| full time | -0.112  (-0.379;0.173) | -0.871 | 0.384 | -0.167  (-0.358;0.023) | 0.088  (-0.042;0.363) |
| part time/hourly | 0.058  (-0.128;0.275) | 0.565 | 0.572 | 0.000  (-0.169;0.174) | 0.000  (-0.013;0.150) |
| Physical activity (h/week) | 0.067  (-0.055;0.191) | 1.100 | 0.271 | 0.066  (-0.058;0.188) | 0.021  (-0.002;0.112) |
| Smoking status (ref. never smoker) |  |  |  |  |  |
| current smoker | 0.117  (-0.142;0.383) | 0.894 | 0.371 | -0.058  (-0.195;0.085) | -0.032  (-0.097;0.104) |
| former smoker | 0.107  (-0.182;0.375) | 0.805 | 0.421 | 0.108  (-0.034;0.248) | 0.055  (-0.029;0.272) |
| **R-squared (95%CI)** | **0.212 (0.125;0.608)** | |  |  | **SUM = 1.0** |
| **AFTERNOON SNACK** | | | | | |
| Within level (intake level) | | | | | |
| Week/weekend day (y/n) | 0.056  (0.007;0.101) | 2.249 | 0.024 | 0.077  (0.030;0.120) | 0.058  (0.003;0.165) |
| Season (winter/ summer) | 0.033  (-0.014;0.080) | 1.376 | 0.169 | 0.029  (-0.021;0.077) | 0.013  (0.000;0.0750) |
| Special day (y/n) | 0.071  (0.020;0.121) | 2.632 | 0.008 | 0.104  (0.054;0.155) | 0.100  (0.014;0.226) |
| Prior interval (hours) | 0.146  (0.095;0.200) | 5.452 | 0.000 | 0.174  (0.120;0.226) | 0.343  (0.172;0.515) |
| Place of meal (ref. home) |  |  |  |  |  |
| work | -0.159  (-0.227;-0.096) | -5.098 | 0.000 | -0.192  (-0.257;-0.130) | 0.413  (0.185;0.598) |
| restaurant | 0.027  (-0.019;0.072) | 1.159 | 0.246 | 0.047  (0.000;0.090) | 0.017  (-0.002;0.085) |
| other | 0.048  (-0.01;0.103) | 1.556 | 0.120 | 0.092  (0.031;0.151) | 0.060  (-0.004;0.180) |
| **R-squared (95%CI)** | **0.074 (0.050;0.113)** | |  |  | **SUM = 1.0** |
| Between level (participant level) | | | | | |
| BMI (kg/m^2^) | 0.031  (-0.095;0.138) | 0.550 | 0.582 | 0.021  (-0.109;0.127) | 0.020  (-0.003;0.290) |
| Age (years) | -0.022  (-0.207;0.147) | -0.247 | 0.805 | -0.089  (-0.222;0.042) | 0.059  (-0.114;0.471) |
| Sex (M/W) | -0.118  (-0.247;-0.006) | -1.859 | 0.063 | -0.088  (-0.203;0.021) | 0.315  (-0.009;0.589) |
| Education level (ref. no training/ current training) |  |  |  |  |  |
| technical college | 0.008  (-0.112;0.143) | 0.123 | 0.902 | -0.017  (-0.122;0.108) | -0.004  (-0.018;0.272) |
| university | -0.018  (-0.157;0.109) | -0.271 | 0.786 | 0.009  (-0.113;0.119) | -0.005  (-0.02;0.256) |
| Occupation (ref. no job/ retired)^4^ |  |  |  |  |  |
| full time | 0.134  (-0.051;0.322) | 1.467 | 0.142 | 0.130  (-0.005;0.258) | 0.528  (-0.036;0.783) |
| part time/hourly | 0.031  (-0.102;0.178) | 0.430 | 0.667 | -0.010  (-0.148;0.129) | -0.009  (-0.02;0.336) |
| Physical activity (h/week) | 0.060  (-0.054;0.160) | 1.123 | 0.261 | 0.015  (-0.103;0.124) | 0.027  (-0.015;0.297) |
| Smoking status (ref. never smoker) |  |  |  |  |  |
| current smoker | 0.018  (-0.177;0.238) | 0.170 | 0.865 | 0.021  (-0.089;0.156) | 0.011  (-0.081;0.393) |
| former smoker | -0.060  (-0.264;0.133) | -0.588 | 0.556 | -0.039  (-0.164;0.070) | 0.071  (-0.072;0.465) |
| **R-squared (95%CI)** | **0.033 (0.023;0.138)** | |  |  | **SUM = 1.0** |
| **DINNER** | | | | | |
| Within level (intake level) | | | | | |
| Week/weekend day (y/n) | -0.018  (-0.062;0.027) | -0.748 | 0.455 | -0.012  (-0.056;0.034) | 0.009  (-0.001;0.131) |
| Season (winter/ summer) | 0.030  (-0.011;0.073) | 1.380 | 0.167 | 0.030  (-0.012;0.074) | 0.039  (0.000;0.192) |
| Special day (y/n) | 0.049  (-0.002;0.098) | 1.935 | 0.053 | 0.072  (0.018;0.122) | 0.153  (-0.001;0.395) |
| Prior interval (hours) | 0.048  (0.001;0.093) | 2.103 | 0.035 | 0.061  (0.013;0.103) | 0.127  (0.002;0.329) |
| Place of meal (ref. home) |  |  |  |  |  |
| work | -0.059  (-0.124;-0.004) | -2.032 | 0.042 | -0.063  (-0.128;-0.007) | 0.162  (0.002;0.457) |
| restaurant | 0.102  (0.048;0.148) | 4.037 | 0.000 | 0.116  (0.060;0.161) | 0.514  (0.150;0.709) |
| other | -0.010  (-0.080;0.055) | -0.292 | 0.770 | -0.003  (-0.074;0.062) | 0.001  (-0.001;0.231) |
| **R-squared (95%CI)** | **0.023 (0.014;0.046)** | |  |  | **SUM = 1.0** |
| Between level (participant level) | | | | | |
| BMI (kg/m^2^) | 0.220  (0.116;0.323) | 4.377 | 0.000 | 0.215  (0.119;0.317) | 0.187  (0.056;0.326) |
| Age (years) | -0.087  (-0.240;0.065) | -1.181 | 0.238 | -0.065  (-0.174;0.050) | 0.022  (-0.010;0.124) |
| Sex (M/W) | -0.409  (-0.511;-0.315) | -8.074 | 0.000 | -0.420  (-0.511;-0.334) | 0.679  (0.444;0.786) |
| Education level (ref. no training/ current training) |  |  |  |  |  |
| technical college | -0.018  (-0.143;0.089) | -0.308 | 0.758 | -0.158  (-0.277;-0.058) | 0.011  (-0.029;0.121) |
| university | 0.115  (-0.006;0.252) | 1.798 | 0.072 | 0.193  (0.090;0.298) | 0.088  (-0.002;0.239) |
| Occupation (ref. no job/ retired)^4^ |  |  |  |  |  |
| full time | 0.040  (-0.127;0.192) | 0.509 | 0.610 | 0.120  (-0.013;0.235) | 0.019  (-0.028;0.135) |
| part time/hourly | 0.057  (-0.053;0.160) | 1.059 | 0.290 | -0.007  (-0.105;0.091) | -0.002  (-0.011;0.049) |
| Physical activity (h/week) | 0.070  (-0.026;0.158) | 1.527 | 0.127 | -0.025  (-0.121;0.068) | -0.007  (-0.015;0.038) |
| Smoking status (ref. never smoker) |  |  |  |  |  |
| current smoker | 0.000  (-0.179;0.181) | 0.001 | 0.999 | -0.147  (-0.249;-0.041) | 0.000  (-0.074;0.136) |
| former smoker | 0.000  (-0.177;0.169) | -0.003 | 0.998 | 0.141  (0.037;0.241) | 0.000  (-0.070;0.132) |
| **R-squared (95%CI)** | **0.253 (0.194;0.388)** | |  |  | **SUM = 1.0** |

^1^ for dichotomous variables, the information shown is for the underlined category (reference category not underlined)

^2^ all 95% confidence intervals (95%CI) – for beta-weights, correlations, r-squared, and Pratt Index – are bootstrap confidence intervals based on 1000 bootstrap samples

^3^ might not add up to 100% due to rounding errors from parameter estimates

^4^ full time: > 35h/week; part time/hourly: < 35 h/week

# Table 8: Random intercept Multilevel Regression Analysis and Corresponding Pratt for **fat intake** (g/meal)

| **Predictor**^1^ | **Beta-weight (95%CI)**^2^ | **t-test** | **p-value** | **Correlation**  **(95%CI)** | **Pratt Index**^2^ **(95%CI)** |
| --- | --- | --- | --- | --- | --- |
| **BREAKFAST** | | | | | |
| Within level (intake level) | | | | | |
| Week/weekend day (y/n) | 0.116  (0.069;0.159) | 5.238 | 0.000 | 0.127  (0.081;0.171) | 0.320  (0.121;0.509) |
| Season (winter/ summer) | -0.019  (-0.063;0.022) | -0.893 | 0.372 | -0.021  (-0.068;0.019) | 0.009  (0.000;0.082) |
| Special day (y/n) | 0.008  (-0.051;0.066) | 0.276 | 0.782 | 0.045  (-0.013;0.101) | 0.008  (-0.012;0.125) |
| Prior interval (hours) | -0.003  (-0.059;0.053) | -0.117 | 0.907 | 0.008  (-0.05;0.065) | -0.001  (-0.001;0.093) |
| Place of meal (ref. home) |  |  |  |  |  |
| work | -0.108  (-0.200;-0.025) | -2.435 | 0.015 | -0.117  (-0.212;-0.034) | 0.275  (0.026;0.586) |
| restaurant | 0.127  (0.083;0.176) | 5.777 | 0.000 | 0.135  (0.091;0.180) | 0.373  (0.148;0.590) |
| other | 0.017  (-0.053;0.078) | 0.514 | 0.608 | 0.019  (-0.050;0.079) | 0.007  (0.000;0.113) |
| **R-squared (95%CI)** | **0.046 (0.029;0.082)** | |  |  | **SUM = 1.0** |
| Between level (participant level) | | | | | |
| BMI (kg/m^2^) | -0.075  (-0.164;0.018) | -1.645 | 0.100 | -0.056  (-0.148;0.035) | 0.035  (-0.001;0.167) |
| Age (years) | 0.081  (-0.046;0.197) | 1.374 | 0.170 | 0.108  (0.025;0.190) | 0.073  (-0.018;0.237) |
| Sex (M/W) | -0.337  (-0.424;-0.249) | -7.742 | 0.000 | -0.305  (-0.386;-0.228) | 0.857  (0.540;0.940) |
| Education level (ref. no training/ current training) |  |  |  |  |  |
| technical college | -0.036  (-0.128;0.055) | -0.773 | 0.439 | -0.035  (-0.114;0.040) | 0.011  (-0.004;0.102) |
| university | -0.115  (-0.208;-0.024) | -2.410 | 0.016 | -0.027  (-0.112;0.057) | 0.026  (-0.021;0.149) |
| Occupation (ref. no job/ retired)^4^ |  |  |  |  |  |
| full time | 0.035  (-0.107;0.163) | 0.517 | 0.605 | -0.032  (-0.125;0.062) | -0.009  (-0.037;0.095) |
| part time/hourly | 0.046  (-0.034;0.127) | 1.191 | 0.234 | -0.019  (-0.084;0.047) | -0.007  (-0.020;0.041) |
| Physical activity (h/week) | 0.064  (-0.019;0.145) | 1.499 | 0.134 | 0.040  (-0.047;0.121) | 0.021  (-0.003;0.129) |
| Smoking status (ref. never smoker) |  |  |  |  |  |
| current smoker | 0.058  (-0.103;0.223) | 0.733 | 0.464 | -0.038  (-0.123;0.038) | -0.018  (-0.061;0.082) |
| former smoker | 0.024  (-0.128;0.180) | 0.309 | 0.758 | 0.052  (-0.022;0.137) | 0.010  (-0.030;0.137) |
| **R-squared (95%CI)** | **0.120 (0.088;0.198)** | |  |  | **SUM = 1.0** |
| **LUNCH** | | | | | |
| Within level (intake level) | | | | | |
| Week/weekend day (y/n) | 0.059  (0.013;0.103) | 2.665 | 0.008 | 0.067  (0.020;0.110) | 0.180  (0.012;0.426) |
| Season (winter/ summer) | 0.028  (-0.016;0.074) | 1.249 | 0.212 | 0.012  (-0.033;0.058) | 0.015  (-0.003;0.183) |
| Special day (y/n) | 0.045  (0.001;0.089) | 1.979 | 0.048 | 0.046  (0.002;0.087) | 0.094  (0.000;0.304) |
| Prior interval (hours) | 0.025  (-0.021;0.069) | 1.133 | 0.257 | 0.032  (-0.01;0.076) | 0.036  (-0.001;0.189) |
| Place of meal (ref. home) |  |  |  |  |  |
| work | -0.090  (-0.159;-0.021) | -2.572 | 0.010 | -0.093  (-0.16;-0.024) | 0.380  (0.028;0.691) |
| restaurant | 0.030  (-0.017;0.077) | 1.303 | 0.193 | 0.060  (0.022;0.102) | 0.082  (-0.011;0.335) |
| other | -0.078  (-0.139;-0.022) | -2.701 | 0.007 | -0.061  (-0.120;-0.005) | 0.216  (0.006;0.510) |
| **R-squared (95%CI)** | **0.022 (0.012;0.046)** | |  |  | **SUM = 1.0** |
| Between level (participant level) | | | | | |
| BMI (kg/m^2^) | -0.019  (-0.177;0.148) | -0.254 | 0.800 | 0.009  (-0.160;0.180) | -0.001  (-0.002;0.107) |
| Age (years) | 0.244  (0.000;0.563) | 1.960 | 0.050 | 0.320  (0.142;0.559) | 0.355  (0.000;0.661) |
| Sex (M/W) | -0.355  (-0.628;-0.190) | -3.338 | 0.001 | -0.334  (-0.599;-0.175) | 0.539  (0.180;0.713) |
| Education level (ref. no training/ current training) |  |  |  |  |  |
| technical college | -0.029  (-0.207;0.144) | -0.356 | 0.722 | -0.026  (-0.189;0.120) | 0.003  (-0.005;0.094) |
| university | -0.086  (-0.265;0.070) | -1.065 | 0.287 | -0.031  (-0.176;0.121) | 0.012  (-0.013;0.143) |
| Occupation (ref. no job/ retired)^4^ |  |  |  |  |  |
| full time | -0.007  (-0.272;0.294) | -0.054 | 0.957 | -0.189  (-0.401;0.012) | 0.006  (-0.098;0.263) |
| part time/hourly | 0.018  (-0.177;0.238) | 0.197 | 0.844 | -0.085  (-0.278;0.069) | -0.007  (-0.029;0.154) |
| Physical activity (h/week) | 0.115  (-0.026;0.294) | 1.499 | 0.134 | 0.138  (0.010;0.288) | 0.072  (-0.001;0.257) |
| Smoking status (ref. never smoker) |  |  |  |  |  |
| current smoker | 0.096  (-0.169;0.377) | 0.724 | 0.469 | -0.015  (-0.184;0.126) | -0.007  (-0.054;0.138) |
| former smoker | 0.059  (-0.206;0.356) | 0.438 | 0.661 | 0.066  (-0.085;0.247) | 0.018  (-0.035;0.208) |
| **R-squared (95%CI)** | **0.220 (0.118;0.716)** | |  |  | **SUM = 1.0** |
| **AFTERNOON SNACK** | | | | | |
| Within level (intake level) | | | | | |
| Week/weekend day (y/n) | 0.073  (0.029;0.120) | 2.879 | 0.004 | 0.102  (0.057;0.149) | 0.090  (0.020;0.206) |
| Season (winter/ summer) | -0.013  (-0.056;0.034) | -0.548 | 0.584 | -0.014  (-0.061;0.035) | 0.002  (0.000;0.040) |
| Special day (y/n) | 0.066  (0.014;0.117) | 2.468 | 0.014 | 0.107  (0.056;0.157) | 0.085  (0.011;0.207) |
| Prior interval (hours) | 0.131  (0.082;0.182) | 5.245 | 0.000 | 0.158  (0.107;0.209) | 0.249  (0.113;0.386) |
| Place of meal (ref. home) |  |  |  |  |  |
| work | -0.175  (-0.244;-0.108) | -5.298 | 0.000 | -0.212  (-0.279;-0.147) | 0.447  (0.219;0.627) |
| restaurant | 0.045  (-0.004;0.091) | 1.887 | 0.059 | 0.065  (0.014;0.111) | 0.035  (0.000;0.118) |
| other | 0.073  (0.025;0.120) | 2.890 | 0.004 | 0.111  (0.064;0.160) | 0.098  (0.020;0.222) |
| **R-squared (95%CI)** | **0.083 (0.062;0.123)** | |  |  | **SUM = 1.0** |
| Between level (participant level) | | | | | |
| BMI (kg/m^2^) | -0.010  (-0.135;0.106) | -0.159 | 0.874 | -0.022  (-0.144;0.097) | 0.003  (-0.002;0.198) |
| Age (years) | -0.008  (-0.169;0.168) | -0.095 | 0.924 | -0.016  (-0.136;0.104) | 0.002  (-0.043;0.203) |
| Sex (M/W) | -0.234  (-0.357;-0.112) | -3.620 | 0.000 | -0.169  (-0.279;-0.053) | 0.628  (0.094;0.774) |
| Education level (ref. no training/ current training) |  |  |  |  |  |
| technical college | -0.003  (-0.125;0.133) | -0.043 | 0.966 | -0.004  (-0.114;0.106) | 0.000  (-0.007;0.177) |
| university | -0.069  (-0.195;0.059) | -1.073 | 0.283 | -0.028  (-0.136;0.083) | 0.031  (-0.015;0.282) |
| Occupation (ref. no job/ retired)^4^ |  |  |  |  |  |
| full time | 0.052  (-0.124;0.251) | 0.551 | 0.581 | 0.041  (-0.085;0.185) | 0.034  (-0.032;0.355) |
| part time/hourly | 0.051  (-0.061;0.154) | 0.963 | 0.336 | 0.015  (-0.094;0.106) | 0.012  (-0.014;0.160) |
| Physical activity (h/week) | 0.070  (-0.03;0.162) | 1.394 | 0.163 | 0.037  (-0.062;0.125) | 0.041  (-0.007;0.200) |
| Smoking status (ref. never smoker) |  |  |  |  |  |
| current smoker | -0.074  (-0.246;0.108) | -0.859 | 0.391 | 0.034  (-0.078;0.158) | -0.040  (-0.12;0.201) |
| former smoker | -0.206  (-0.389;-0.036) | -2.410 | 0.016 | -0.089  (-0.218;0.022) | 0.291  (-0.021;0.667) |
| **R-squared (95%CI)** | **0.063 (0.045;0.177)** | |  |  | **SUM = 1.0** |
| **DINNER** | | | | | |
| Within level (intake level) | | | | | |
| Week/weekend day (y/n) | -0.030  (-0.076;0.016) | -1.290 | 0.197 | -0.027  (-0.072;0.018) | 0.058  (0.000;0.304) |
| Season (winter/ summer) | 0.038  (-0.005;0.080) | 1.718 | 0.086 | 0.038  (-0.005;0.080) | 0.103  (0.000;0.340) |
| Special day (y/n) | 0.031  (-0.017;0.077) | 1.347 | 0.178 | 0.040  (0.001;0.082) | 0.089  (-0.004;0.372) |
| Prior interval (hours) | 0.028  (-0.019;0.072) | 1.175 | 0.240 | 0.038  (-0.008;0.081) | 0.076  (-0.002;0.384) |
| Place of meal (ref. home) |  |  |  |  |  |
| work | -0.074  (-0.163;0.003) | -1.660 | 0.097 | -0.074  (-0.162;0.004) | 0.391  (0.001;0.750) |
| restaurant | 0.058  (0.020;0.096) | 2.925 | 0.003 | 0.066  (0.028;0.103) | 0.273  (0.035;0.582) |
| other | -0.027  (-0.092;0.034) | -0.885 | 0.376 | -0.020  (-0.085;0.040) | 0.039  (-0.001;0.360) |
| **R-squared (95%CI)** | **0.014 (0.008;0.039)** | |  |  | **SUM = 1.0** |
| Between level (participant level) | | | | | |
| BMI (kg/m^2^) | 0.105  (0.005;0.204) | 2.226 | 0.026 | 0.113  (0.015;0.199) | 0.054  (0.000;0.154) |
| Age (years) | 0.081  (-0.05;0.218) | 1.183 | 0.237 | 0.102  (-0.003;0.205) | 0.038  (-0.011;0.150) |
| Sex (M/W) | -0.415  (-0.511;-0.319) | -8.240 | 0.000 | -0.422  (-0.509;-0.337) | 0.796  (0.534;0.895) |
| Education level (ref. no training/ current training) |  |  |  |  |  |
| technical college | -0.099  (-0.218;0.012) | -1.660 | 0.097 | -0.159  (-0.275;-0.050) | 0.072  (-0.003;0.228) |
| university | -0.003  (-0.118;0.117) | -0.050 | 0.960 | 0.109  (0.008;0.213) | -0.001  (-0.027;0.091) |
| Occupation (ref. no job/ retired)^4^ |  |  |  |  |  |
| full time | 0.005  (-0.135;0.150) | 0.070 | 0.944 | -0.042  (-0.151;0.072) | -0.001  (-0.019;0.072) |
| part time/hourly | 0.137  (0.040;0.236) | 2.717 | 0.007 | 0.053  (-0.041;0.14) | 0.033  (-0.008;0.124) |
| Physical activity (h/week) | 0.060  (-0.033;0.142) | 1.401 | 0.161 | 0.010  (-0.082;0.095) | 0.003  (-0.007;0.054) |
| Smoking status (ref. never smoker) |  |  |  |  |  |
| current smoker | -0.052  (-0.22;0.120) | -0.598 | 0.550 | -0.134  (-0.231;-0.033) | 0.032  (-0.049;0.166) |
| former smoker | -0.046  (-0.239;0.129) | -0.522 | 0.602 | 0.120  (0.022;0.219) | -0.025  (-0.082;0.095) |
| **R-squared (95%CI)** | **0.220 (0.171;0.331)** | |  |  | **SUM = 1.0** |

^1^ for dichotomous variables, the information shown is for the underlined category (reference category not underlined)

^2^ all 95% confidence intervals (95%CI) – for beta-weights, correlations, r-squared, and Pratt Index – are bootstrap confidence intervals based on 1000 bootstrap samples

^3^ might not add up to 100% due to rounding errors from parameter estimates

^4^ full time: > 35h/week; part time/hourly: < 35 h/week

# Table 9: Random intercept Multilevel Regression Analysis and Corresponding Pratt for **energy intake** (kcal/meal); sensitivity analysis adjusting for energy misreporting^1^

| **Predictor**^2^ | **Beta-weight (95%CI)**^3^ | **t-test** | | **p-value** | **Correlation**  **(95%CI)** | **Pratt Index**^4^ **(95%CI)** |
| --- | --- | --- | --- | --- | --- | --- |
| **BREAKFAST** | | | | | | |
| Within level (intake level) | | | | | | |
| Week/weekend day (y/n) | 0.108  (0.057;0.166) | 3.867 | | 0.000 | 0.123  (0.071;0.179) | 0.271  (0.085;0.492) |
| Season (winter/ summer) | -0.038  (-0.086;0.010) | -1.572 | | 0.116 | -0.040  (-0.087;0.009) | 0.031  (0.000;0.174) |
| Special day (y/n) | 0.008  (-0.056;0.072) | 0.246 | | 0.806 | 0.051  (-0.009;0.114) | 0.008  (-0.015;0.142) |
| Prior interval (hours) | -0.024  (-0.083;0.037) | -0.787 | | 0.431 | -0.012  (-0.076;0.054) | 0.006  (-0.001;0.142) |
| Place of meal (ref. home) |  |  | |  |  |  |
| work | -0.138  (-0.268;-0.019) | -2.339 | | 0.019 | -0.146  (-0.279;-0.023) | 0.411  (0.014;0.721) |
| restaurant | 0.104  (0.057;0.154) | 4.454 | | 0.000 | 0.113  (0.067;0.163) | 0.240  (0.056;0.497) |
| other | 0.043  (-0.004;0.091) | 1.799 | | 0.072 | 0.044  (-0.003;0.089) | 0.039  (0.000;0.168) |
| **R-squared (95%CI)** | **0.049 (0.028;0.105)** | | |  |  | **SUM = 1.0** |
| Between level (participant level) | | | | | | |
| BMI (kg/m^2^) | 0.000  (-0.099;0.099) | 0.009 | | 0.992 | -0.033  (-0.132;0.067) | 0.000  (-0.005;0.047) |
| Age (years) | 0.082  (-0.054;0.205) | 1.252 | | 0.211 | 0.206  (0.116;0.293) | 0.068  (-0.032;0.190) |
| Sex (M/W) | -0.309  (-0.406;-0.215) | -6.372 | | 0.000 | -0.353  (-0.433;-0.279) | 0.436  (0.231;0.585) |
| Education level (ref. no training/ current training) |  |  | |  |  |  |
| technical college | -0.011  (-0.099;0.081) | -0.220 | | 0.826 | -0.070  (-0.154;0.019) | 0.003  (-0.010;0.055) |
| university | -0.011  (-0.108;0.081) | -0.223 | | 0.824 | 0.057  (-0.030;0.143) | -0.003  (-0.012;0.039) |
| Occupation (ref. no job/ retired)^5^ |  |  | |  |  |  |
| full time | 0.052  (-0.111;0.195) | 0.677 | | 0.499 | -0.045  (-0.149;0.059) | -0.009  (-0.025;0.058) |
| part time/hourly | -0.079  (-0.187;0.042) | -1.395 | | 0.163 | -0.169  (-0.282;-0.046) | 0.053  (-0.008;0.174) |
| Physical activity (h/week) | 0.052  (-0.03;0.127) | 1.315 | | 0.189 | 0.051  (-0.029;0.124) | 0.011  (-0.001;0.058) |
| Smoking status (ref. never smoker) |  |  | |  |  |  |
| current smoker | 0.268  (0.063;0.452) | 2.687 | | 0.007 | 0.040  (-0.051;0.125) | 0.043  (-0.035;0.168) |
| former smoker | 0.204  (0.008;0.396) | 2.071 | | 0.038 | 0.059  (-0.032;0.152) | 0.048  (-0.010;0.179) |
| Energy misreporting |  |  | |  |  |  |
| EI/TEE < 0.81 | -0.154  (-0.251;-0.065) | -3.088 | | 0.002 | -0.263  (-0.347;-0.181) | 0.162  (0.048;0.305) |
| EI/TEE > 1.19 | 0.176  (0.109;0.246) | 5.712 | | 0.000 | 0.267  (0.197;0.330) | 0.188  (0.083;0.303) |
| **R-squared (95%CI)** | **0.250 (0.201;0.355)** | | |  |  | **SUM = 1.0** |
| **LUNCH** | | | | | | |
| Within level (intake level) | | | | | | |
| Week/weekend day (y/n) | 0.035  (-0.014;0.083) | 1.389 | | 0.165 | 0.049  (0.000;0.097) | 0.059  (-0.001;0.253) |
| Season (winter/ summer) | 0.012  (-0.038;0.061) | 0.489 | | 0.625 | 0.000  (-0.050;0.050) | 0.000  (-0.002;0.103) |
| Special day (y/n) | 0.040  (-0.011;0.089) | 1.570 | | 0.116 | 0.051  (-0.002;0.099) | 0.070  (0.000;0.288) |
| Prior interval (hours) | 0.058  (0.014;0.103) | 2.489 | | 0.013 | 0.069  (0.025;0.112) | 0.138  (0.013;0.405) |
| Place of meal (ref. home) |  |  | |  |  |  |
| work | -0.144  (-0.220;-0.061) | -3.290 | | 0.001 | -0.151  (-0.225;-0.073) | 0.750  (0.232;0.897) |
| restaurant | -0.005  (-0.064;0.048) | -0.171 | | 0.864 | 0.027  (-0.026;0.077) | -0.005  (-0.012;0.141) |
| other | -0.016  (-0.067;0.036) | -0.577 | | 0.564 | 0.008  (-0.040;0.058) | -0.004  (-0.007;0.123) |
| **R-squared (95%CI)** | **0.029 (0.013;0.063)** | | |  |  | **SUM = 1.0** |
| Between level (participant level) | | | | | | |
| BMI (kg/m^2^) | 0.077  (-0.059;0.229) | 1.116 | | 0.265 | -0.011  (-0.142;0.121) | -0.002  (-0.010;0.055) |
| Age (years) | 0.017  (-0.186;0.194) | 0.175 | | 0.861 | 0.257  (0.098;0.404) | 0.011  (-0.064;0.156) |
| Sex (M/W) | -0.377  (-0.567;-0.230) | -5.255 | | 0.000 | -0.382  (-0.556;-0.250) | 0.347  (0.142;0.506) |
| Education level (ref. no training/ current training) |  |  | |  |  |  |
| technical college | -0.027  (-0.156;0.113) | -0.411 | | 0.681 | -0.054  (-0.196;0.086) | 0.004  (-0.005;0.054) |
| university | -0.084  (-0.227;0.057) | -1.155 | | 0.248 | -0.015  (-0.149;0.130) | 0.003  (-0.009;0.061) |
| Occupation (ref. no job/ retired)^5^ |  |  | |  |  |  |
| full time | -0.033  (-0.233;0.177) | -0.322 | | 0.747 | -0.166  (-0.359;0.013) | 0.013  (-0.032;0.138) |
| part time/hourly | 0.017  (-0.15;0.175) | 0.217 | | 0.828 | -0.061  (-0.225;0.095) | -0.002  (-0.011;0.069) |
| Physical activity (h/week) | 0.145  (0.013;0.280) | 2.261 | | 0.024 | 0.162  (0.041;0.290) | 0.057  (0.002;0.137) |
| Smoking status (ref. never smoker) |  |  | |  |  |  |
| current smoker | 0.186  (-0.064;0.449) | 1.494 | | 0.135 | 0.076  (-0.057;0.202) | 0.034  (-0.014;0.153) |
| former smoker | 0.067  (-0.187;0.351) | 0.520 | | 0.603 | -0.014  (-0.143;0.130) | -0.002  (-0.021;0.068) |
| Energy misreporting |  |  | |  |  |  |
| EI/TEE < 0.81 | -0.372  (-0.539;-0.227) | -5.107 | | 0.000 | -0.462  (-0.603;-0.343) | 0.414  (0.182;0.576) |
| EI/TEE > 1.19 | 0.159  (0.058;0.278) | 3.332 | | 0.001 | 0.311  (0.212;0.440) | 0.119  (0.03;0.216) |
| **R-squared (95%CI)** | **0.415 (0.310;0.784)** | | |  |  | **SUM = 1.0** |
| **AFTERNOON SNACK** | | | | | | |
| Within level (intake level) | | | | | | |
| Week/weekend day (y/n) | 0.100  (0.049;0.152) | 3.803 | | 0.000 | 0.134  (0.084;0.184) | 0.114  (0.033;0.225) |
| Season (winter/ summer) | 0.035  (-0.014;0.084) | 1.432 | | 0.152 | 0.029  (-0.022;0.077) | 0.009  (0.000;0.051) |
| Special day (y/n) | 0.053  (-0.003;0.102) | 1.929 | | 0.054 | 0.101  (0.043;0.150) | 0.045  (-0.001;0.123) |
| Prior interval (hours) | 0.167  (0.116;0.214) | 6.549 | | 0.000 | 0.206  (0.153;0.254) | 0.292  (0.158;0.432) |
| Place of meal (ref. home) |  |  | |  |  |  |
| work | -0.207  (-0.275;-0.136) | -6.037 | | 0.000 | -0.256  (-0.322;-0.186) | 0.449  (0.244;0.614) |
| restaurant | 0.019  (-0.041;0.072) | 0.643 | | 0.520 | 0.044  (-0.018;0.094) | 0.007  (-0.002;0.057) |
| other | 0.079  (0.028;0.134) | 2.968 | | 0.003 | 0.129  (0.081;0.183) | 0.086  (0.020;0.204) |
| **R-squared (95%CI)** | **0.118 (0.087;0.162)** | | |  |  | **SUM = 1.0** |
| Between level (participant level) | | | | | | |
| BMI (kg/m^2^) | 0.086  (-0.037;0.221) | 1.356 | | 0.175 | -0.039  (-0.173;0.089) | -0.015  (-0.024;0.075) |
| Age (years) | -0.100  (-0.284;0.092) | -1.100 | | 0.271 | 0.020  (-0.112;0.163) | -0.009  (-0.056;0.100) |
| Sex (M/W) | -0.232  (-0.357;-0.106) | -3.663 | | 0.000 | -0.227  (-0.351;-0.117) | 0.228  (0.048;0.389) |
| Education level (ref. no training/ current training) |  |  | |  |  |  |
| technical college | 0.078  (-0.062;0.208) | 1.166 | | 0.244 | -0.010  (-0.142;0.112) | -0.003  (-0.017;0.080) |
| university | 0.042  (-0.095;0.179) | 0.597 | | 0.551 | 0.075  (-0.045;0.204) | 0.014  (-0.008;0.110) |
| Occupation (ref. no job/ retired)^5^ |  |  | |  |  |  |
| full time | 0.047  (-0.163;0.263) | 0.459 | | 0.646 | 0.023  (-0.138;0.169) | 0.005  (-0.016;0.128) |
| part time/hourly | 0.033  (-0.082;0.147) | 0.592 | | 0.554 | -0.004  (-0.122;0.104) | -0.001  (-0.009;0.055) |
| Physical activity (h/week) | 0.016  (-0.091;0.122) | 0.312 | | 0.755 | -0.005  (-0.116;0.103) | 0.000  (-0.003;0.048) |
| Smoking status (ref. never smoker) |  |  | |  |  |  |
| current smoker | 0.182  (-0.030;0.398) | 1.621 | | 0.105 | 0.115  (0.001;0.241) | 0.091  (-0.005;0.292) |
| former smoker | 0.020  (-0.209;0.236) | 0.178 | | 0.859 | -0.076  (-0.204;0.041) | -0.007  (-0.060;0.119) |
| Energy misreporting |  |  | |  |  |  |
| EI/TEE < 0.81 | -0.370  (-0.520;-0.249) | -5.603 | | 0.000 | -0.383  (-0.530;-0.263) | 0.613  (0.299;0.740) |
| EI/TEE > 1.19 | 0.101  (-0.030;0.234) | 1.550 | | 0.121 | 0.188  (0.049;0.323) | 0.082  (-0.006;0.237) |
| **R-squared (95%CI)** | **0.231 (0.163;0.444)** | | |  |  | **SUM = 1.0** |
| **DINNER** | | | | | | |
| Within level (intake level) | | | | | | |
| Week/weekend day (y/n) | -0.035  (-0.087;0.012) | | -1.359 | 0.174 | -0.025  (-0.074;0.024) | 0.027  (-0.001;0.168) |
| Season (winter/ summer) | 0.039  (-0.007;0.088) | | 1.573 | 0.116 | 0.042  (-0.005;0.092) | 0.050  (0.000;0.194) |
| Special day (y/n) | 0.061  (0.010;0.109) | | 2.417 | 0.016 | 0.082  (0.036;0.132) | 0.152  (0.010;0.361) |
| Prior interval (hours) | 0.074  (0.027;0.119) | | 3.086 | 0.002 | 0.088  (0.041;0.132) | 0.197  (0.031;0.398) |
| Place of meal (ref. home) |  | |  |  |  |  |
| work | -0.081  (-0.169;-0.007) | | -1.962 | 0.050 | -0.085  (-0.172;-0.010) | 0.209  (0.003;0.550) |
| restaurant | 0.101  (0.055;0.149) | | 4.221 | 0.000 | 0.116  (0.069;0.163) | 0.355  (0.115;0.573) |
| other | -0.008  (-0.082;0.063) | | -0.227 | 0.821 | 0.003  (-0.069;0.072) | -0.001  (-0.001;0.172) |
| **R-squared (95%CI)** | **0.033 (0.021;0.062)** | | |  |  | **SUM = 1.0** |
| Between level (participant level) | | | | | | |
| BMI (kg/m^2^) | 0.181  (0.065;0.294) | 3.183 | | 0.001 | 0.092  (-0.018;0.204) | 0.041  (-0.003;0.124) |
| Age (years) | -0.216  (-0.355;-0.067) | -3.096 | | 0.002 | -0.045  (-0.149;0.061) | 0.024  (-0.021;0.102) |
| Sex (M/W) | -0.410  (-0.496;-0.326) | -8.825 | | 0.000 | -0.482  (-0.565;-0.399) | 0.482  (0.320;0.590) |
| Education level (ref. no training/ current training) |  |  | |  |  |  |
| technical college | 0.033  (-0.073;0.138) | 0.597 | | 0.551 | -0.100  (-0.202;-0.001) | -0.008  (-0.022;0.030) |
| university | 0.075  (-0.039;0.192) | 1.284 | | 0.199 | 0.152  (0.049;0.258) | 0.028  (-0.007;0.105) |
| Occupation (ref. no job/ retired)^5^ |  |  | |  |  |  |
| full time | -0.003  (-0.147;0.141) | -0.044 | | 0.965 | 0.074  (-0.041;0.181) | -0.001  (-0.017;0.044) |
| part time/hourly | 0.052  (-0.054;0.153) | 0.981 | | 0.326 | 0.020  (-0.089;0.123) | 0.003  (-0.004;0.040) |
| Physical activity (h/week) | 0.045  (-0.043;0.127) | 1.030 | | 0.303 | -0.037  (-0.128;0.055) | -0.004  (-0.010;0.018) |
| Smoking status (ref. never smoker) |  |  | |  |  |  |
| current smoker | 0.027  (-0.166;0.230) | 0.273 | | 0.785 | -0.169  (-0.267;-0.067) | -0.011  (-0.07;0.087) |
| former smoker | 0.092  (-0.103;0.281) | 0.953 | | 0.340 | 0.180  (0.074;0.275) | 0.040  (-0.027;0.154) |
| Energy misreporting |  |  | |  |  |  |
| EI/TEE < 0.81 | -0.302  (-0.420;-0.190) | -5.103 | | 0.000 | -0.310  (-0.413;-0.209) | 0.228  (0.102;0.353) |
| EI/TEE > 1.19 | 0.236  (0.159;0.313) | 6.623 | | 0.000 | 0.309  (0.234;0.383) | 0.178  (0.092;0.267) |
| **R-squared (95%CI)** | **0.410 (0.340;0.556)** | | |  |  | **SUM = 1.0** |

EI: energy intake; TEE: total energy expenditure

^1^ n=682 participants with activity sensor data

^2^ for dichotomous variables, the information shown is for the underlined category (reference category not underlined)

^3^ all 95% confidence intervals (95%CI) – for beta-weights, correlations, r-squared, and Pratt Index – are bootstrap confidence intervals based on 1000 bootstrap samples

^4^ might not add up to 100% due to rounding errors from parameter estimates

^5^ full time: > 35h/week; part time/hourly: < 35 h/week.

# Table 10: Random intercept Multilevel Regression Analysis and Corresponding Pratt for **carbohydrate intake** (g/meal); sensitivity analysis adjusting for energy misreporting^1^

| **Predictor**^2^ | **Beta-weight (95%CI)**^3^ | **t-test** | **p-value** | **Correlation**  **(95%CI)** | **Pratt Index**^4^ **(95%CI)** |
| --- | --- | --- | --- | --- | --- |
| **BREAKFAST** | | | | | |
| Within level (intake level) | | | | | |
| Week/weekend day (y/n) | 0.068  (0.016;0.119) | 2.567 | 0.010 | 0.081  (0.028;0.129) | 0.157  (0.016;0.464) |
| Season (winter/ summer) | -0.066  (-0.110;-0.015) | -2.809 | 0.005 | -0.066  (-0.109;-0.016) | 0.124  (0.006;0.427) |
| Special day (y/n) | -0.007  (-0.073;0.049) | -0.223 | 0.824 | 0.019  (-0.044;0.075) | -0.004  (-0.010;0.138) |
| Prior interval (hours) | -0.010  (-0.071;0.049) | -0.323 | 0.747 | 0.001  (-0.063;0.063) | 0.000  (-0.001;0.154) |
| Place of meal (ref. home) |  |  |  |  |  |
| work | -0.144  (-0.257;-0.026) | -2.394 | 0.017 | -0.148  (-0.263;-0.030) | 0.609  (0.045;0.842) |
| restaurant | 0.048  (-0.004;0.104) | 1.786 | 0.074 | 0.051  (0.003;0.102) | 0.070  (0.000;0.326) |
| other | 0.037  (-0.003;0.079) | 1.719 | 0.086 | 0.036  (-0.005;0.076) | 0.038  (0.000;0.194) |
| **R-squared (95%CI)** | **0.035 (0.016;0.087)** | |  |  | **SUM = 1.0** |
| Between level (participant level) | | | | | |
| BMI (kg/m^2^) | -0.037  (-0.138;0.056) | -0.740 | 0.459 | -0.066  (-0.162;0.020) | 0.011  (-0.004;0.087) |
| Age (years) | 0.087  (-0.052;0.222) | 1.255 | 0.209 | 0.226  (0.133;0.320) | 0.088  (-0.036;0.241) |
| Sex (M/W) | -0.236  (-0.327;-0.141) | -5.024 | 0.000 | -0.268  (-0.338;-0.193) | 0.284  (0.116;0.432) |
| Education level (ref. no training/ current training) |  |  |  |  |  |
| technical college | 0.024  (-0.079;0.119) | 0.485 | 0.628 | -0.053  (-0.152;0.033) | -0.006  (-0.015;0.045) |
| university | 0.059  (-0.034;0.161) | 1.217 | 0.224 | 0.092  (0.005;0.173) | 0.024  (-0.004;0.100) |
| Occupation (ref. no job/ retired)^5^ |  |  |  |  |  |
| full time | 0.013  (-0.143;0.154) | 0.176 | 0.860 | -0.073  (-0.174;0.025) | -0.004  (-0.029;0.089) |
| part time/hourly | -0.112  (-0.239;0.005) | -1.888 | 0.059 | -0.186  (-0.314;-0.065) | 0.093  (-0.002;0.273) |
| Physical activity (h/week) | 0.053  (-0.038;0.139) | 1.156 | 0.248 | 0.068  (-0.015;0.155) | 0.016  (-0.001;0.084) |
| Smoking status (ref. never smoker) |  |  |  |  |  |
| current smoker | 0.374  (0.19;0.563) | 3.854 | 0.000 | 0.118  (0.031;0.206) | 0.198  (0.035;0.367) |
| former smoker | 0.270  (0.071;0.457) | 2.761 | 0.006 | 0.018  (-0.076;0.104) | 0.022  (-0.060;0.159) |
| Energy misreporting |  |  |  |  |  |
| EI/TEE < 0.81 | -0.121  (-0.234;-0.009) | -2.195 | 0.028 | -0.241  (-0.335;-0.145) | 0.131  (0.006;0.300) |
| EI/TEE > 1.19 | 0.144  (0.074;0.207) | 4.376 | 0.000 | 0.220  (0.151;0.289) | 0.142  (0.051;0.241) |
| **R-squared (95%CI)** | **0.223 (0.178;0.329)** | |  |  | **SUM = 1.0** |
| **LUNCH** | | | | | |
| Within level (intake level) | | | | | |
| Week/weekend day (y/n) | 0.010  (-0.038;0.052) | 0.387 | 0.699 | 0.019  (-0.028;0.062) | 0.021  (-0.003;0.343) |
| Season (winter/ summer) | -0.039  (-0.086;0.006) | -1.608 | 0.108 | -0.039  (-0.085;0.007) | 0.169  (0.000;0.551) |
| Special day (y/n) | 0.029  (-0.017;0.074) | 1.263 | 0.207 | 0.028  (-0.014;0.071) | 0.090  (-0.002;0.412) |
| Prior interval (hours) | 0.002  (-0.05;0.050) | 0.068 | 0.946 | 0.006  (-0.046;0.057) | 0.001  (-0.001;0.329) |
| Place of meal (ref. home) |  |  |  |  |  |
| work | -0.062  (-0.138;0.012) | -1.557 | 0.119 | -0.062  (-0.135;0.010) | 0.427  (0.002;0.735) |
| restaurant | -0.049  (-0.111;0.012) | -1.500 | 0.134 | -0.037  (-0.098;0.021) | 0.201  (-0.002;0.560) |
| other | 0.023  (-0.027;0.072) | 0.965 | 0.334 | 0.036  (-0.011;0.081) | 0.092  (-0.007;0.453) |
| **R-squared (95%CI)** | **0.009 (0.004;0.031)** | |  |  | **SUM = 1.0** |
| Between level (participant level) | | | | | |
| BMI (kg/m^2^) | -0.003  (-0.156;0.143) | -0.035 | 0.972 | -0.091  (-0.24;0.039) | 0.001  (-0.010;0.078) |
| Age (years) | 0.096  (-0.125;0.326) | 0.890 | 0.374 | 0.200  (0.018;0.388) | 0.050  (-0.031;0.215) |
| Sex (M/W) | -0.379  (-0.557;-0.215) | -4.357 | 0.000 | -0.384  (-0.530;-0.241) | 0.381  (0.138;0.530) |
| Education level (ref. no training/ current training) |  |  |  |  |  |
| technical college | -0.046  (-0.238;0.130) | -0.515 | 0.607 | -0.118  (-0.267;0.040) | 0.014  (-0.012;0.122) |
| university | -0.013  (-0.189;0.161) | -0.152 | 0.880 | 0.090  (-0.063;0.249) | -0.003  (-0.017;0.080) |
| Occupation (ref. no job/ retired)^5^ |  |  |  |  |  |
| full time | 0.117  (-0.154;0.376) | 0.857 | 0.391 | -0.057  (-0.276;0.150) | -0.017  (-0.049;0.129) |
| part time/hourly | 0.079  (-0.095;0.262) | 0.924 | 0.356 | -0.041  (-0.20;0.121) | -0.008  (-0.019;0.065) |
| Physical activity (h/week) | 0.139  (-0.008;0.281) | 1.919 | 0.055 | 0.123  (-0.022;0.264) | 0.045  (0.000;0.154) |
| Smoking status (ref. never smoker) |  |  |  |  |  |
| current smoker | 0.309  (0.022;0.596) | 2.068 | 0.039 | 0.134  (-0.016;0.289) | 0.108  (-0.004;0.311) |
| former smoker | 0.132  (-0.138;0.420) | 0.898 | 0.369 | -0.041  (-0.192;0.106) | -0.014  (-0.061;0.073) |
| Energy misreporting |  |  |  |  |  |
| EI/TEE < 0.81 | -0.298  (-0.481;-0.139) | -3.619 | 0.000 | -0.415  (-0.577;-0.278) | 0.324  (0.095;0.528) |
| EI/TEE > 1.19 | 0.154  (0.021;0.290) | 2.401 | 0.016 | 0.281  (0.16;0.409) | 0.113  (0.009;0.232) |
| **R-squared (95%CI)** | **0.382 (0.276;0.715)** | |  |  | **SUM = 1.0** |
| **AFTERNOON SNACK** | | | | | |
| Within level (intake level) | | | | | |
| Week/weekend day (y/n) | 0.071  (0.020;0.118) | 2.693 | 0.007 | 0.092  (0.041;0.139) | 0.100  (0.012;0.252) |
| Season (winter/ summer) | 0.050  (0.000;0.098) | 2.009 | 0.045 | 0.046  (-0.005;0.094) | 0.035  (0.000;0.128) |
| Special day (y/n) | 0.027  (-0.025;0.079) | 1.028 | 0.304 | 0.064  (0.007;0.114) | 0.027  (-0.005;0.131) |
| Prior interval (hours) | 0.132  (0.082;0.184) | 5.188 | 0.000 | 0.160  (0.106;0.211) | 0.325  (0.139;0.503) |
| Place of meal (ref. home) |  |  |  |  |  |
| work | -0.144  (-0.214;-0.079) | -4.198 | 0.000 | -0.180  (-0.247;-0.116) | 0.399  (0.150;0.618) |
| restaurant | 0.009  (-0.043;0.058) | 0.335 | 0.738 | 0.024  (-0.027;0.073) | 0.003  (-0.002;0.060) |
| other | 0.069  (0.021;0.115) | 2.765 | 0.006 | 0.105  (0.057;0.151) | 0.111  (0.020;0.257) |
| **R-squared (95%CI)** | **0.065 (0.044;0.102)** | |  |  | **SUM = 1.0** |
| Between level (participant level) | | | | | |
| BMI (kg/m^2^) | 0.098  (-0.034;0.237) | 1.456 | 0.145 | -0.014  (-0.165;0.123) | -0.007  (-0.023;0.122) |
| Age (years) | -0.111  (-0.302;0.116) | -1.112 | 0.266 | 0.041  (-0.092;0.184) | -0.023  (-0.080;0.102) |
| Sex (M/W) | -0.241  (-0.386;-0.108) | -3.635 | 0.000 | -0.218  (-0.352;-0.092) | 0.261  (0.048;0.432) |
| Education level (ref. no training/ current training) |  |  |  |  |  |
| technical college | 0.096  (-0.053;0.240) | 1.373 | 0.170 | 0.020  (-0.107;0.137) | 0.010  (-0.016;0.121) |
| university | 0.037  (-0.106;0.182) | 0.489 | 0.625 | 0.047  (-0.088;0.187) | 0.009  (-0.006;0.115) |
| Occupation (ref. no job/ retired)^5^ |  |  |  |  |  |
| full time | -0.009  (-0.207;0.225) | -0.085 | 0.932 | -0.028  (-0.186;0.123) | 0.001  (-0.020;0.163) |
| part time/hourly | 0.044  (-0.078;0.165) | 0.789 | 0.430 | 0.017  (-0.096;0.127) | 0.004  (-0.007;0.074) |
| Physical activity (h/week) | 0.034  (-0.088;0.136) | 0.631 | 0.528 | 0.023  (-0.101;0.135) | 0.004  (-0.002;0.072) |
| Smoking status (ref. never smoker) |  |  |  |  |  |
| current smoker | 0.186  (-0.021;0.431) | 1.667 | 0.096 | 0.114  (-0.009;0.273) | 0.105  (-0.004;0.354) |
| former smoker | 0.023  (-0.185;0.246) | 0.214 | 0.831 | -0.074  (-0.221;0.046) | -0.008  (-0.061;0.125) |
| Energy misreporting |  |  |  |  |  |
| EI/TEE < 0.81 | -0.326  (-0.499;-0.189) | -4.316 | 0.000 | -0.343  (-0.515;-0.203) | 0.556  (0.213;0.708) |
| EI/TEE > 1.19 | 0.096  (-0.022;0.214) | 1.532 | 0.125 | 0.180  (0.050;0.311) | 0.086  (-0.005;0.219) |
| **R-squared (95%CI)** | **0.201 (0.137;0.434)** | |  |  | **SUM = 1.0** |
| **DINNER** | | | | | |
| Within level (intake level) | | | | | |
| Week/weekend day (y/n) | -0.010  (-0.063;0.041) | -0.389 | 0.697 | -0.009  (-0.063;0.040) | 0.008  (0.000;0.240) |
| Season (winter/ summer) | 0.026  (-0.022;0.071) | 1.084 | 0.278 | 0.028  (-0.017;0.073) | 0.066  (0.000;0.318) |
| Special day (y/n) | 0.049  (0.002;0.096) | 2.032 | 0.042 | 0.046  (-0.003;0.093) | 0.205  (0.001;0.520) |
| Prior interval (hours) | 0.072  (0.017;0.128) | 2.602 | 0.009 | 0.076  (0.018;0.132) | 0.497  (0.028;0.770) |
| Place of meal (ref. home) |  |  |  |  |  |
| work | -0.037  (-0.114;0.018) | -1.190 | 0.234 | -0.039  (-0.116;0.013) | 0.131  (0.000;0.524) |
| restaurant | 0.001  (-0.085;0.074) | 0.019 | 0.985 | 0.016  (-0.07;0.089) | 0.001  (-0.007;0.411) |
| other | -0.031  (-0.101;0.028) | -0.981 | 0.326 | -0.019  (-0.083;0.036) | 0.054  (-0.004;0.413) |
| **R-squared (95%CI)** | **0.011 (0.007;0.032)** | |  |  | **SUM = 1.0** |
| Between level (participant level) | | | | | |
| BMI (kg/m^2^) | 0.059  (-0.077;0.186) | 0.879 | 0.379 | -0.025  (-0.158;0.095) | -0.005  (-0.011;0.055) |
| Age (years) | -0.167  (-0.336;0.013) | -1.905 | 0.057 | 0.006  (-0.119;0.124) | -0.003  (-0.049;0.093) |
| Sex (M/W) | -0.343  (-0.479;-0.230) | -5.511 | 0.000 | -0.412  (-0.532;-0.314) | 0.456  (0.240;0.617) |
| Education level (ref. no training/ current training) |  |  |  |  |  |
| technical college | 0.027  (-0.102;0.166) | 0.402 | 0.687 | -0.091  (-0.211;0.025) | -0.008  (-0.022;0.055) |
| university | 0.076  (-0.051;0.198) | 1.189 | 0.234 | 0.146  (0.033;0.249) | 0.036  (-0.007;0.131) |
| Occupation (ref. no job/ retired)^5^ |  |  |  |  |  |
| full time | -0.027  (-0.223;0.167) | -0.278 | 0.781 | 0.044  (-0.102;0.166) | -0.004  (-0.019;0.075) |
| part time/hourly | -0.014  (-0.142;0.105) | -0.217 | 0.828 | -0.031  (-0.149;0.074) | 0.001  (-0.005;0.055) |
| Physical activity (h/week) | 0.004  (-0.096;0.106) | 0.076 | 0.939 | -0.053  (-0.153;0.042) | -0.001  (-0.008;0.038) |
| Smoking status (ref. never smoker) |  |  |  |  |  |
| current smoker | 0.042  (-0.179;0.269) | 0.363 | 0.716 | -0.103  (-0.220;0.009) | -0.014  (-0.062;0.091) |
| former smoker | 0.072  (-0.154;0.299) | 0.637 | 0.524 | 0.118  (0.011;0.226) | 0.027  (-0.034;0.136) |
| Energy misreporting |  |  |  |  |  |
| EI/TEE < 0.81 | -0.290  (-0.414;-0.165) | -4.457 | 0.000 | -0.331  (-0.448;-0.219) | 0.310  (0.113;0.461) |
| EI/TEE > 1.19 | 0.211  (0.130;0.309) | 4.911 | 0.000 | 0.298  (0.212;0.395) | 0.203  (0.083;0.319) |
| **R-squared (95%CI)** | **0.310 (0.242;0.501)** | |  |  | **SUM = 1.0** |

EI: energy intake; TEE: total energy expenditure

^1^ n=682 participants with activity sensor data

^2^ for dichotomous variables, the information shown is for the underlined category (reference category not underlined)

^3^ all 95% confidence intervals (95%CI) – for beta-weights, correlations, r-squared, and Pratt Index – are bootstrap confidence intervals based on 1000 bootstrap samples

^4^ might not add up to 100% due to rounding errors from parameter estimates

^5^ full time: > 35h/week; part time/hourly: < 35 h/week.

# Table 11: Random intercept Multilevel Regression Analysis and Corresponding Pratt for **protein intake** (g/meal); sensitivity analysis adjusting for energy misreporting^1^

| **Predictor**^2^ | **Beta-weight (95%CI)**^3^ | **t-test** | **p-value** | **Correlation**  **(95%CI)** | **Pratt Index**^4^ **(95%CI)** |
| --- | --- | --- | --- | --- | --- |
| **BREAKFAST** | | | | | |
| Within level (intake level) | | | | | |
| Week/weekend day (y/n) | 0.130  (0.077;0.179) | 5.233 | 0.000 | 0.138  (0.088;0.185) | 0.345  (0.136;0.520) |
| Season (winter/ summer) | 0.024  (-0.022;0.067) | 1.057 | 0.290 | 0.020  (-0.025;0.062) | 0.009  (0.000;0.070) |
| Special day (y/n) | 0.012  (-0.051;0.070) | 0.387 | 0.698 | 0.062  (0.000;0.119) | 0.014  (-0.016;0.149) |
| Prior interval (hours) | -0.046  (-0.107;0.011) | -1.475 | 0.140 | -0.036  (-0.099;0.024) | 0.032  (-0.001;0.188) |
| Place of meal (ref. home) |  |  |  |  |  |
| work | -0.089  (-0.183;0.000) | -1.862 | 0.063 | -0.098  (-0.194;-0.010) | 0.168  (0.002;0.470) |
| restaurant | 0.140  (0.091;0.186) | 6.386 | 0.000 | 0.152  (0.110;0.195) | 0.409  (0.184;0.622) |
| other | 0.026  (-0.036;0.083) | 0.829 | 0.407 | 0.028  (-0.032;0.085) | 0.014  (0.000;0.120) |
| **R-squared (95%CI)** | **0.052 (0.035;0.087)** | |  |  | **SUM = 1.0** |
| Between level (participant level) | | | | | |
| BMI (kg/m^2^) | 0.071  (-0.017;0.169) | 1.487 | 0.137 | 0.048  (-0.037;0.137) | 0.027  (-0.002;0.143) |
| Age (years) | -0.018  (-0.162;0.127) | -0.246 | 0.806 | 0.110  (0.012;0.201) | -0.016  (-0.089;0.137) |
| Sex (M/W) | -0.239  (-0.336;-0.139) | -4.655 | 0.000 | -0.281  (-0.360;-0.196) | 0.529  (0.198;0.701) |
| Education level (ref. no training/ current training) |  |  |  |  |  |
| technical college | -0.009  (-0.106;0.091) | -0.168 | 0.867 | -0.080  (-0.163;0.007) | 0.006  (-0.021;0.105) |
| university | 0.049  (-0.052;0.148) | 0.932 | 0.351 | 0.093  (0.005;0.177) | 0.036  (-0.011;0.152) |
| Occupation (ref. no job/ retired)^5^ |  |  |  |  |  |
| full time | -0.027  (-0.187;0.130) | -0.341 | 0.733 | -0.028  (-0.125;0.074) | 0.006  (-0.016;0.138) |
| part time/hourly | -0.070  (-0.183;0.048) | -1.326 | 0.185 | -0.110  (-0.206;-0.007) | 0.061  (-0.010;0.223) |
| Physical activity (h/week) | 0.031  (-0.067;0.121) | 0.700 | 0.484 | 0.019  (-0.078;0.099) | 0.005  (-0.003;0.087) |
| Smoking status (ref. never smoker) |  |  |  |  |  |
| current smoker | 0.138  (-0.026;0.312) | 1.536 | 0.124 | -0.019  (-0.113;0.069) | -0.021  (-0.087;0.107) |
| former smoker | 0.012  (-0.049;0.302) | 1.345 | 0.179 | 0.076  (-0.010;0.171) | 0.007  (-0.011;0.272) |
| Energy misreporting |  |  |  |  |  |
| EI/TEE < 0.81 | -0.114  (-0.210;-0.024) | -2.319 | 0.020 | -0.165  (-0.259;-0.079) | 0.148  (0.015;0.323) |
| EI/TEE > 1.19 | 0.114  (0.034;0.194) | 2.853 | 0.004 | 0.166  (0.089;0.246) | 0.149  (0.020;0.288) |
| **R-squared (95%CI)** | **0.127 (0.099;0.215)** | |  |  | **SUM = 0.94** |
| **LUNCH** | | | | | |
| Within level (intake level) | | | | | |
| Week/weekend day (y/n) | 0.072  (0.027;0.120) | 2.894 | 0.004 | 0.085  (0.040;0.135) | 0.153  (0.029;0.344) |
| Season (winter/ summer) | 0.011  (-0.037;0.059) | 0.464 | 0.643 | -0.008  (-0.058;0.039) | -0.002  (-0.004;0.073) |
| Special day (y/n) | 0.008  (-0.041;0.057) | 0.336 | 0.737 | 0.025  (-0.026;0.071) | 0.005  (-0.004;0.095) |
| Prior interval (hours) | 0.035  (-0.008;0.076) | 1.544 | 0.122 | 0.047  (0.004;0.089) | 0.041  (-0.001;0.168) |
| Place of meal (ref. home) |  |  |  |  |  |
| work | -0.162  (-0.230;-0.091) | -4.552 | 0.000 | -0.170  (-0.234;-0.102) | 0.689  (0.323;0.852) |
| restaurant | 0.037  (-0.017;0.092) | 1.327 | 0.185 | 0.068  (0.018;0.119) | 0.063  (-0.007;0.261) |
| other | -0.049  (-0.111;0.008) | -1.643 | 0.100 | -0.034  (-0.095;0.022) | 0.042  (-0.001;0.232) |
| **R-squared (95%CI)** | **0.040 (0.023;0.072)** | |  |  | **SUM = 1.0** |
| Between level (participant level) | | | | | |
| BMI (kg/m^2^) | 0.114  (-0.052;0.277) | 1.364 | 0.172 | 0.034  (-0.140;0.190) | 0.011  (-0.009;0.106) |
| Age (years) | -0.032  (-0.276;0.192) | -0.289 | 0.773 | 0.233  (0.056;0.426) | -0.021  (-0.105;0.155) |
| Sex (M/W) | -0.310  (-0.547;-0.133) | -3.108 | 0.002 | -0.346  (-0.562;-0.185) | 0.296  (0.084;0.470) |
| Education level (ref. no training/ current training) |  |  |  |  |  |
| technical college | -0.092  (-0.266;0.066) | -1.152 | 0.250 | -0.091  (-0.249;0.059) | 0.023  (-0.003;0.113) |
| university | -0.085  (-0.279;0.065) | -1.003 | 0.316 | -0.012  (-0.167;0.139) | 0.003  (-0.013;0.076) |
| Occupation (ref. no job/ retired)^5^ |  |  |  |  |  |
| full time | -0.140  (-0.378;0.107) | -1.083 | 0.279 | -0.218  (-0.414;-0.032) | 0.084  (-0.023;0.301) |
| part time/hourly | 0.062  (-0.125;0.244) | 0.695 | 0.487 | 0.023  (-0.163;0.191) | 0.004  (-0.006;0.092) |
| Physical activity (h/week) | 0.096  (-0.028;0.235) | 1.511 | 0.131 | 0.117  (-0.012;0.250) | 0.031  (-0.001;0.106) |
| Smoking status (ref. never smoker) |  |  |  |  |  |
| current smoker | 0.064  (-0.224;0.372) | 0.450 | 0.653 | -0.027  (-0.177;0.121) | -0.005  (-0.035;0.083) |
| former smoker | 0.056  (-0.231;0.352) | 0.391 | 0.696 | 0.062  (-0.081;0.222) | 0.010  (-0.024;0.142) |
| Energy misreporting |  |  |  |  |  |
| EI/TEE < 0.81 | -0.346  (-0.555;-0.167) | -3.606 | 0.000 | -0.416  (-0.629;-0.264) | 0.398  (0.127;0.601) |
| EI/TEE > 1.19 | 0.183  (0.076;0.332) | 3.082 | 0.002 | 0.313  (0.205;0.478) | 0.158  (0.040;0.285) |
| **R-squared (95%CI)** | **0.362 (0.243;0.826)** | |  |  | **SUM = 1.0** |
| **AFTERNOON SNACK** | | | | | |
| Within level (intake level) | | | | | |
| Week/weekend day (y/n) | 0.050  (-0.004;0.105) | 1.900 | 0.057 | 0.075  (0.023;0.128) | 0.046  (-0.001;0.152) |
| Season (winter/ summer) | 0.025  (-0.026;0.072) | 0.971 | 0.331 | 0.022  (-0.028;0.073) | 0.007  (0.000;0.058) |
| Special day (y/n) | 0.060  (0.001;0.114) | 2.047 | 0.041 | 0.099  (0.040;0.152) | 0.072  (0.001;0.194) |
| Prior interval (hours) | 0.163  (0.106;0.218) | 5.929 | 0.000 | 0.191  (0.134;0.248) | 0.380  (0.187;0.559) |
| Place of meal (ref. home) |  |  |  |  |  |
| work | -0.163  (-0.228;-0.100) | -4.825 | 0.000 | -0.199  (-0.263;-0.137) | 0.396  (0.188;0.588) |
| restaurant | 0.038  (-0.008;0.085) | 1.544 | 0.123 | 0.057  (0.010;0.102) | 0.026  (-0.001;0.100) |
| other | 0.058  (-0.005;0.117) | 1.841 | 0.066 | 0.097  (0.036;0.159) | 0.069  (-0.002;0.203) |
| **R-squared (95%CI)** | **0.082 (0.058;0.120)** | |  |  | **SUM = 1.0** |
| Between level (participant level) | | | | | |
| BMI (kg/m^2^) | 0.103  (-0.028;0.237) | 1.512 | 0.130 | 0.002  (-0.118;0.134) | 0.002  (-0.025;0.174) |
| Age (years) | -0.061  (-0.236;0.128) | -0.655 | 0.512 | -0.113  (-0.248;0.021) | 0.058  (-0.044;0.279) |
| Sex (M/W) | -0.101  (-0.241;0.038) | -1.418 | 0.156 | -0.103  (-0.235;0.022) | 0.088  (-0.003;0.327) |
| Education level (ref. no training/ current training) |  |  |  |  |  |
| technical college | 0.078  (-0.065;0.217) | 1.102 | 0.270 | 0.022  (-0.100;0.147) | 0.015  (-0.015;0.181) |
| university | 0.022  (-0.139;0.172) | 0.287 | 0.774 | 0.033  (-0.098;0.162) | 0.006  (-0.009;0.151) |
| Occupation (ref. no job/ retired)^5^ |  |  |  |  |  |
| full time | 0.187  (-0.021;0.396) | 1.763 | 0.078 | 0.139  (-0.011;0.303) | 0.220  (-0.005;0.489) |
| part time/hourly | 0.116  (0.005;0.238) | 1.924 | 0.054 | 0.070  (-0.030;0.177) | 0.069  (-0.004;0.241) |
| Physical activity (h/week) | 0.029  (-0.090;0.142) | 0.479 | 0.632 | -0.020  (-0.139;0.096) | -0.005  (-0.011;0.096) |
| Smoking status (ref. never smoker) |  |  |  |  |  |
| current smoker | 0.028  (-0.211;0.249) | 0.239 | 0.811 | 0.033  (-0.093;0.174) | 0.008  (-0.047;0.203) |
| former smoker | -0.060  (-0.287;0.176) | -0.521 | 0.603 | -0.055  (-0.201;0.075) | 0.028  (-0.041;0.249) |
| Energy misreporting |  |  |  |  |  |
| EI/TEE < 0.81 | -0.213  (-0.362;-0.070) | -2.916 | 0.004 | -0.188  (-0.333;-0.060) | 0.339  (0.030;0.575) |
| EI/TEE > 1.19 | 0.133  (0.006;0.257) | 2.057 | 0.040 | 0.152  (0.026;0.281) | 0.171  (0.002;0.374) |
| **R-squared (95%CI)** | **0.118 (0.085;0.293)** | |  |  | **SUM = 1.0** |
| **DINNER** | | | | | |
| Within level (intake level) | | | | | |
| Week/weekend day (y/n) | -0.018  (-0.070;0.032) | -0.694 | 0.488 | -0.011  (-0.063;0.039) | 0.009  (-0.001;0.189) |
| Season (winter/ summer) | 0.046  (0.001;0.094) | 1.892 | 0.059 | 0.045  (0.002;0.094) | 0.099  (0.001;0.337) |
| Special day (y/n) | 0.051  (-0.005;0.105) | 1.826 | 0.068 | 0.065  (0.008;0.119) | 0.158  (-0.001;0.435) |
| Prior interval (hours) | 0.046  (-0.004;0.096) | 1.868 | 0.062 | 0.059  (0.009;0.107) | 0.129  (0.001;0.383) |
| Place of meal (ref. home) |  |  |  |  |  |
| work | -0.060  (-0.130;-0.002) | -1.917 | 0.055 | -0.063  (-0.132;-0.004) | 0.180  (0.002;0.512) |
| restaurant | 0.084  (0.026;0.132) | 2.928 | 0.003 | 0.097  (0.042;0.146) | 0.388  (0.054;0.630) |
| other | -0.025  (-0.105;0.043) | -0.657 | 0.511 | -0.015  (-0.095;0.053) | 0.018  (-0.002;0.316) |
| **R-squared (95%CI)** | **0.021 (0.012;0.047)** | |  |  | **SUM = 1.0** |
| Between level (participant level) | | | | | |
| BMI (kg/m^2^) | 0.274  (0.167;0.386) | 4.973 | 0.000 | 0.190  (0.084;0.300) | 0.151  (0.043;0.275) |
| Age (years) | -0.176  (-0.342;-0.021) | -2.212 | 0.027 | -0.045  (-0.163;0.074) | 0.023  (-0.023;0.122) |
| Sex (M/W) | -0.334  (-0.445;-0.217) | -5.978 | 0.000 | -0.420  (-0.521;-0.321) | 0.407  (0.206;0.540) |
| Education level (ref. no training/ current training) |  |  |  |  |  |
| technical college | 0.039  (-0.089;0.167) | 0.629 | 0.529 | -0.120  (-0.249;-0.005) | -0.014  (-0.032;0.051) |
| university | 0.161  (0.024;0.295) | 2.333 | 0.020 | 0.205  (0.088;0.329) | 0.096  (0.007;0.234) |
| Occupation (ref. no job/ retired)^5^ |  |  |  |  |  |
| full time | 0.007  (-0.164;0.185) | 0.089 | 0.929 | 0.083  (-0.05;0.214) | 0.002  (-0.024;0.091) |
| part time/hourly | 0.028  (-0.086;0.133) | 0.504 | 0.614 | -0.005  (-0.119;0.093) | 0.000  (-0.005;0.034) |
| Physical activity (h/week) | 0.042  (-0.055;0.131) | 0.914 | 0.361 | -0.034  (-0.143;0.058) | -0.004  (-0.010;0.025) |
| Smoking status (ref. never smoker) |  |  |  |  |  |
| current smoker | -0.047  (-0.234;0.136) | -0.505 | 0.613 | -0.161  (-0.273;-0.056) | 0.022  (-0.046;0.148) |
| former smoker | 0.000  (-0.195;0.180) | 0.005 | 0.996 | 0.151  (0.043;0.255) | 0.000  (-0.060;0.107) |
| Energy misreporting |  |  |  |  |  |
| EI/TEE < 0.81 | -0.250  (-0.366;-0.137) | -4.181 | 0.000 | -0.232  (-0.342;-0.129) | 0.168  (0.049;0.310) |
| EI/TEE > 1.19 | 0.208  (0.125;0.300) | 4.768 | 0.000 | 0.248  (0.165;0.336) | 0.150  (0.060;0.246) |
| **R-squared (95%CI)** | **0.345 (0.280;0.508)** | |  |  | **SUM = 1.0** |

EI: energy intake; TEE: total energy expenditure

^1^ n=682 participants with activity sensor data

^2^ for dichotomous variables, the information shown is for the underlined category (reference category not underlined)

^3^ all 95% confidence intervals (95%CI) – for beta-weights, correlations, r-squared, and Pratt Index – are bootstrap confidence intervals based on 1000 bootstrap samples

^4^ might not add up to 100% due to rounding errors from parameter estimates

^5^ full time: > 35h/week; part time/hourly: < 35 h/week.

# Table 12: Random intercept Multilevel Regression Analysis and Corresponding Pratt for **fat intake** (g/meal); sensitivity analysis adjusting for energy misreporting^1^

| **Predictor**^2^ | **Beta-weight (95%CI)**^3^ | **t-test** | **p-value** | **Correlation**  **(95%CI)** | **Pratt Index**^4^ **(95%CI)** |
| --- | --- | --- | --- | --- | --- |
| **BREAKFAST** | | | | | |
| Within level (intake level) | | | | | |
| Week/weekend day (y/n) | 0.131  (0.080;0.176) | 5.458 | 0.000 | 0.147  (0.096;0.189) | 0.344  (0.129;0.543 |
| Season (winter/ summer) | -0.027  (-0.069;0.016) | -1.229 | 0.219 | -0.032  (-0.075;0.013) | 0.015  (0.000;0.081) |
| Special day (y/n) | 0.017  (-0.053;0.080) | 0.478 | 0.632 | 0.066  (0.001;0.124) | 0.020  (-0.017;0.142) |
| Prior interval (hours) | 0.026  (-0.038;0.083) | 0.868 | 0.385 | 0.038  (-0.026;0.094) | 0.018  (-0.001;0.130) |
| Place of meal (ref. home) |  |  |  |  |  |
| work | -0.116  (-0.207;-0.026) | -2.465 | 0.014 | -0.128  (-0.221;-0.037) | 0.265  (0.021;0.558) |
| restaurant | 0.130  (0.077;0.188) | 4.829 | 0.000 | 0.142  (0.092;0.197) | 0.330  (0.115;0.552) |
| other | 0.015  (-0.066;0.095) | 0.372 | 0.710 | 0.015  (-0.066;0.090) | 0.004  (0.000;0.143) |
| **R-squared (95%CI)** | **0.056 (0.037;0.094)** | |  |  | **SUM = 1.0** |
| Between level (participant level) | | | | | |
| BMI (kg/m^2^) | -0.010  (-0.105;0.084) | -0.217 | 0.828 | -0.042  (-0.134;0.058) | 0.003  (-0.005;0.074) |
| Age (years) | 0.015  (-0.125;0.144) | 0.233 | 0.816 | 0.085  (-0.007;0.176) | 0.008  (-0.043;0.120) |
| Sex (M/W) | -0.303  (-0.392;-0.211) | -6.464 | 0.000 | -0.306  (-0.394;-0.225) | 0.591  (0.328;0.726) |
| Education level (ref. no training/ current training) |  |  |  |  |  |
| technical college | -0.015  (-0.111;0.080) | -0.315 | 0.753 | -0.026  (-0.105;0.059) | 0.002  (-0.005;0.056) |
| university | -0.094  (-0.194;0.004) | -1.817 | 0.069 | -0.025  (-0.116;0.061) | 0.015  (-0.012;0.120) |
| Occupation (ref. no job/ retired)^5^ |  |  |  |  |  |
| full time | 0.058  (-0.086;0.206) | 0.814 | 0.416 | -0.016  (-0.108;0.079) | -0.006  (-0.029;0.079) |
| part time/hourly | 0.048  (-0.045;0.134) | 1.083 | 0.279 | -0.016  (-0.089;0.049) | -0.005  (-0.015;0.035) |
| Physical activity (h/week) | 0.064  (-0.023;0.145) | 1.448 | 0.147 | 0.042  (-0.054;0.132) | 0.017  (-0.003;0.102) |
| Smoking status (ref. never smoker) |  |  |  |  |  |
| current smoker | 0.082  (-0.083;0.258) | 0.937 | 0.349 | -0.025  (-0.110;0.058) | -0.013  (-0.052;0.071) |
| former smoker | 0.044  (-0.122;0.217) | 0.503 | 0.615 | 0.040  (-0.051;0.130) | 0.011  (-0.021;0.121) |
| Energy misreporting |  |  |  |  |  |
| EI/TEE < 0.81 | -0.114  (-0.213;-0.025) | -2.308 | 0.021 | -0.187  (-0.272;-0.105) | 0.136  (0.014;0.294) |
| EI/TEE > 1.19 | 0.160  (0.083;0.224) | 4.499 | 0.000 | 0.238  (0.164;0.299) | 0.243  (0.083;0.359) |
| **R-squared (95%CI)** | **0.157 (0.122;0.242)** | |  |  | **SUM = 1.0** |
| **LUNCH** | | | | | |
| Within level (intake level) | | | | | |
| Week/weekend day (y/n) | 0.068  (0.021;0.116) | 2.893 | 0.004 | 0.075  (0.029;0.123) | 0.268  (0.032;0.536) |
| Season (winter/ summer) | 0.028  (-0.024;0.073) | 1.109 | 0.267 | 0.012  (-0.039;0.056) | 0.018  (-0.003;0.181) |
| Special day (y/n) | 0.036  (-0.009;0.083) | 1.535 | 0.125 | 0.046  (0.001;0.093) | 0.087  (-0.001;0.320) |
| Prior interval (hours) | 0.045  (0.001;0.094) | 1.944 | 0.052 | 0.053  (0.009;0.101) | 0.126  (0.002;0.389) |
| Place of meal (ref. home) |  |  |  |  |  |
| work | -0.079  (-0.155;-0.004) | -2.070 | 0.038 | -0.087  (-0.161;-0.017) | 0.362  (0.008;0.699) |
| restaurant | 0.027  (-0.028;0.080) | 1.023 | 0.306 | 0.051  (0.005;0.098) | 0.072  (-0.010;0.350) |
| other | -0.043  (-0.099;0.015) | -1.457 | 0.145 | -0.028  (-0.082;0.027) | 0.063  (-0.003;0.309) |
| **R-squared (95%CI)** | **0.019 (0.010;0.042)** | |  |  | **SUM = 1.0** |
| Between level (participant level) | | | | | |
| BMI (kg/m^2^) | 0.066  (-0.105;0.224) | 0.858 | 0.391 | -0.009  (-0.183;0.135) | -0.002  (-0.009;0.071) |
| Age (years) | 0.116  (-0.105;0.358) | 1.044 | 0.296 | 0.338  (0.153;0.549) | 0.110  (-0.055;0.356) |
| Sex (M/W) | -0.250  (-0.478;-0.064) | -2.505 | 0.012 | -0.296  (-0.529;-0.119) | 0.208  (0.028;0.385) |
| Education level (ref. no training/ current training) |  |  |  |  |  |
| technical college | -0.080  (-0.246;0.081) | -1.011 | 0.312 | -0.065  (-0.243;0.082) | 0.015  (-0.004;0.097) |
| university | -0.085  (-0.247;0.079) | -1.070 | 0.285 | -0.033  (-0.177;0.119) | 0.008  (-0.009;0.100) |
| Occupation (ref. no job/ retired)^5^ |  |  |  |  |  |
| full time | -0.090  (-0.339;0.164) | -0.714 | 0.475 | -0.262  (-0.484;-0.065) | 0.066  (-0.055;0.302) |
| part time/hourly | 0.028  (-0.136;0.191) | 0.354 | 0.724 | -0.038  (-0.220;0.116) | -0.003  (-0.011;0.065) |
| Physical activity (h/week) | 0.098  (-0.044;0.261) | 1.323 | 0.186 | 0.148  (0.012;0.309) | 0.041  (-0.003;0.162) |
| Smoking status (ref. never smoker) |  |  |  |  |  |
| current smoker | 0.026  (-0.229;0.297) | 0.205 | 0.838 | 0.012  (-0.137;0.153) | 0.001  (-0.022;0.077) |
| former smoker | -0.001  (-0.251;0.267) | -0.010 | 0.992 | 0.019  (-0.130;0.168) | 0.000  (-0.021;0.092) |
| Energy misreporting |  |  |  |  |  |
| EI/TEE < 0.81 | -0.340  (-0.554;-0.178) | -3.720 | 0.000 | -0.437  (-0.652;-0.289) | 0.417  (0.148;0.606) |
| EI/TEE > 1.19 | 0.156  (0.050;0.294) | 2.669 | 0.008 | 0.302  (0.190;0.461) | 0.132  (0.028;0.255) |
| **R-squared (95%CI)** | **0.356 (0.223;0.862)** | |  |  | **SUM = 1.0** |
| **AFTERNOON SNACK** | | | | | |
| Within level (intake level) | | | | | |
| Week/weekend day (y/n) | 0.068  (0.015;0.123) | 2.480 | 0.013 | 0.098  (0.044;0.150) | 0.077  (0.007;0.205) |
| Season (winter/ summer) | -0.009  (-0.056;0.041) | -0.351 | 0.725 | -0.012  (-0.063;0.041) | 0.001  (0.000;0.040) |
| Special day (y/n) | 0.043  (-0.019;0.099) | 1.438 | 0.151 | 0.091  (0.030;0.147) | 0.045  (-0.006;0.159) |
| Prior interval (hours) | 0.150  (0.093;0.202) | 5.669 | 0.000 | 0.176  (0.120;0.230) | 0.303  (0.143;0.468) |
| Place of meal (ref. home) |  |  |  |  |  |
| work | -0.173  (-0.243;-0.101) | -4.781 | 0.000 | -0.212  (-0.278;-0.141) | 0.422  (0.192;0.615) |
| restaurant | 0.070  (0.018;0.121) | 2.739 | 0.006 | 0.089  (0.039;0.137) | 0.072  (0.008;0.188) |
| other | 0.071  (0.014;0.124) | 2.603 | 0.009 | 0.103  (0.046;0.155) | 0.084  (0.007;0.202) |
| **R-squared (95%CI)** | **0.087 (0.062;0.126)** | |  |  | **SUM = 1.0** |
| Between level (participant level) | | | | | |
| BMI (kg/m^2^) | 0.066  (-0.079;0.198) | 0.966 | 0.334 | -0.030  (-0.178;0.098) | -0.012  (-0.020;0.114) |
| Age (years) | -0.100  (-0.275;0.069) | -1.179 | 0.238 | -0.025  (-0.145;0.093) | 0.015  (-0.036;0.135) |
| Sex (M/W) | -0.219  (-0.357;-0.088) | -3.283 | 0.001 | -0.195  (-0.328;-0.075) | 0.256  (0.042;0.448) |
| Education level (ref. no training/ current training) |  |  |  |  |  |
| technical college | 0.042  (-0.090;0.178) | 0.660 | 0.509 | 0.028  (-0.085;0.140) | 0.007  (-0.006;0.103) |
| university | -0.041  (-0.174;0.104) | -0.587 | 0.557 | -0.029  (-0.137;0.103) | 0.007  (-0.005;0.115) |
| Occupation (ref. no job/ retired)^5^ |  |  |  |  |  |
| full time | 0.039  (-0.141;0.235) | 0.391 | 0.696 | 0.021  (-0.107;0.166) | 0.005  (-0.014;0.165) |
| part time/hourly | 0.076  (-0.021;0.182) | 1.491 | 0.136 | 0.055  (-0.038;0.136) | 0.025  (-0.003;0.115) |
| Physical activity (h/week) | 0.058  (-0.052;0.166) | 1.093 | 0.275 | 0.029  (-0.083;0.126) | 0.010  (-0.005;0.098) |
| Smoking status (ref. never smoker) |  |  |  |  |  |
| current smoker | -0.074  (-0.268;0.119) | -0.809 | 0.418 | 0.039  (-0.091;0.163) | -0.017  (-0.068;0.113) |
| former smoker | -0.200  (-0.387;-0.018) | -2.237 | 0.025 | -0.099  (-0.225;0.025) | 0.119  (-0.015;0.340) |
| Energy misreporting |  |  |  |  |  |
| EI/TEE < 0.81 | -0.206  (-0.356;-0.080) | -2.968 | 0.003 | -0.231  (-0.368;-0.110) | 0.285  (0.053;0.474) |
| EI/TEE > 1.19 | 0.192  (0.100;0.295) | 4.241 | 0.000 | 0.258  (0.175;0.354) | 0.297  (0.099;0.459) |
| **R-squared (95%CI)** | **0.167 (0.116;0.346)** | |  |  | **SUM = 1.0** |
| **DINNER** | | | | | |
| Within level (intake level) | | | | | |
| Week/weekend day (y/n) | -0.049  (-0.097;0.002) | -1.937 | 0.053 | -0.045  (-0.094;0.004) | 0.105  (0.000;0.355) |
| Season (winter/ summer) | 0.052  (-0.001;0.096) | 2.151 | 0.031 | 0.052  (0.001;0.096) | 0.129  (0.001;0.375) |
| Special day (y/n) | 0.036  (-0.015;0.083) | 1.498 | 0.134 | 0.040  (-0.008;0.083) | 0.069  (0.000;0.284) |
| Prior interval (hours) | 0.039  (-0.007;0.085) | 1.593 | 0.111 | 0.051  (0.002;0.096) | 0.095  (-0.001;0.380) |
| Place of meal (ref. home) |  |  |  |  |  |
| work | -0.082  (-0.177;0.004) | -1.754 | 0.079 | -0.081  (-0.179;0.006) | 0.316  (0.001;0.680) |
| restaurant | 0.060  (0.016;0.100) | 2.758 | 0.006 | 0.068  (0.024;0.107) | 0.194  (0.020;0.470) |
| other | -0.047  (-0.116;0.013) | -1.441 | 0.150 | -0.038  (-0.104;0.024) | 0.085  (0.000;0.381) |
| **R-squared (95%CI)** | **0.021 (0.010;0.051)** | |  |  | **SUM = 1.0** |
| Between level (participant level) | | | | | |
| BMI (kg/m^2^) | 0.217  (0.113;0.322) | 4.273 | 0.000 | 0.117  (0.009;0.213) | 0.067  (0.003;0.151) |
| Age (years) | -0.057  (-0.203;0.085) | -0.779 | 0.436 | 0.112  (-0.005;0.225) | -0.017  (-0.046;0.037) |
| Sex (M/W) | -0.335  (-0.444;-0.222) | -5.886 | 0.000 | -0.417  (-0.518;-0.314) | 0.368  (0.189;0.516) |
| Education level (ref. no training/ current training) |  |  |  |  |  |
| technical college | -0.042  (-0.172;0.082) | -0.675 | 0.500 | -0.122  (-0.244;-0.010) | 0.013  (-0.010;0.090) |
| university | 0.028  (-0.091;0.162) | 0.434 | 0.664 | 0.103  (-0.005;0.217) | 0.008  (-0.011;0.078) |
| Occupation (ref. no job/ retired)^5^ |  |  |  |  |  |
| full time | -0.033  (-0.177;0.129) | -0.422 | 0.673 | -0.081  (-0.200;0.044) | 0.007  (-0.013;0.074) |
| part time/hourly | 0.110  (0.001;0.220) | 2.023 | 0.043 | 0.057  (-0.045;0.157) | 0.017  (-0.003;0.074) |
| Physical activity (h/week) | 0.043  (-0.040;0.138) | 0.973 | 0.331 | 0.017  (-0.076;0.120) | 0.002  (-0.003;0.037) |
| Smoking status (ref. never smoker) |  |  |  |  |  |
| current smoker | -0.055  (-0.259;0.160) | -0.554 | 0.580 | -0.121  (-0.229;-0.013) | 0.018  (-0.034;0.113) |
| former smoker | -0.024  (-0.223;0.183) | -0.247 | 0.805 | 0.111  (0.000;0.219) | -0.007  (-0.046;0.081) |
| Energy misreporting |  |  |  |  |  |
| EI/TEE < 0.81 | -0.341  (-0.468;-0.223) | -5.677 | 0.000 | -0.372  (-0.483;-0.265) | 0.334  (0.156;0.483) |
| EI/TEE > 1.19 | 0.224  (0.146;0.311) | 5.807 | 0.000 | 0.322  (0.251;0.398) | 0.190  (0.095;0.279) |
| **R-squared (95%CI)** | **0.380 (0.316;0.533)** | |  |  | **SUM = 1.0** |

EI: energy intake; TEE: total energy expenditure

^1^ n=682 participants with activity sensor data

^2^ for dichotomous variables, the information shown is for the underlined category (reference category not underlined)

^3^ all 95% confidence intervals (95%CI) – for beta-weights, correlations, r-squared, and Pratt Index – are bootstrap confidence intervals based on 1000 bootstrap samples

^4^ might not add up to 100% due to rounding errors from parameter estimates

^5^ full time: > 35h/week; part time/hourly: < 35 h/week.
